# Restricting efferocytosis pathway to CD91 via phosphatidylserine-targeting chimeric protein augments antitumor immune responses

**DOI:** 10.1016/j.ymthe.2026.05.013

**Published:** 2026-05-26

**Authors:** Yu Mizote, Hiroki Nishita, Kazuki Hashiba, Chisa Okuma, Sachiko Akane, Kenjiro Minomi, Hideaki Tahara

**Affiliations:** 1 Department of Cancer Drug Discovery and Development, Research Center, Osaka International Cancer Institute, 3-1-69 Otemae, Chuo-ku, Osaka 541-8567, Japan; 2 Department of Cancer Immunotherapy, Research Center, Osaka International Cancer Institute, 3-1-69 Otemae, Chuo-ku, Osaka 541-8567, Japan; 3 Nitto joint Research Department for Nucleic Acid Medicine, Research Center, Osaka International Cancer Institute, 3-1-69 Otemae, Chuo-ku, Osaka 541-8567, Japan; 4 Nucleic Acid Medicine Business Division, Corporate Technology Sector, Nitto Denko Corporation, 1-1-2 Shimohozumi, Ibaraki, Osaka 567-8680, Japan; 5 Center for Clinical Research, Osaka International Cancer Institute, 3-1-69 Otemae, Chuo-ku, Osaka 541-8567, Japan

**Keywords:** phosphatidylserine, efferocytosis, CD91, MFG-E8, RAP, chimeric protein, LNP-mRNA, cancer immunotherapy, adaptive immune response

## Abstract

Phosphatidylserine is a representative eat-me signal on the outer membrane of apoptotic cells promoting phagocytosis/efferocytosis. It is recognized by various molecules, such as MFG-E8, which produce an immune-tolerizing environment. In order to develop immunological treatments targeting this mechanism, we designed a novel chimeric protein that consists of a phosphatidylserine-recognition domain from MFG-E8 and receptor-associated protein (RAP), which is a ligand of CD91 known as an immunostimulatory phagocytic receptor. This chimeric protein, PStRAP, can theoretically link phosphatidylserine-exposed apoptotic cells to phagocytes not through authentic immunosuppression but through an immunostimulative route via CD91. The inoculation of dying tumor cells expressing PStRAP, which served as an immunogen, induced potent adaptive immune responses against parental tumor cells in murine tumor models. The presence of PStRAP increased the phagocytosis of apoptotic cells *in vitro* and cross-priming to cytotoxic T-lymphocytes *in vivo* compared with the absence of it. Furthermore, PStRAP protein was supplied systemically by intravenous injection of PStRAP mRNA contained in lipid nanoparticles and along with cytotoxic drug to treat tumor-bearing mice. PStRAP significantly enhanced antitumor effects of cytotoxic drug alone. These results suggest that PStRAP can induce CD91-mediated phagocytosis of the apoptotic tumor cells and initiate meaningful adaptive immune responses against the tumor cells.

## Graphical abstract

**Figure:**
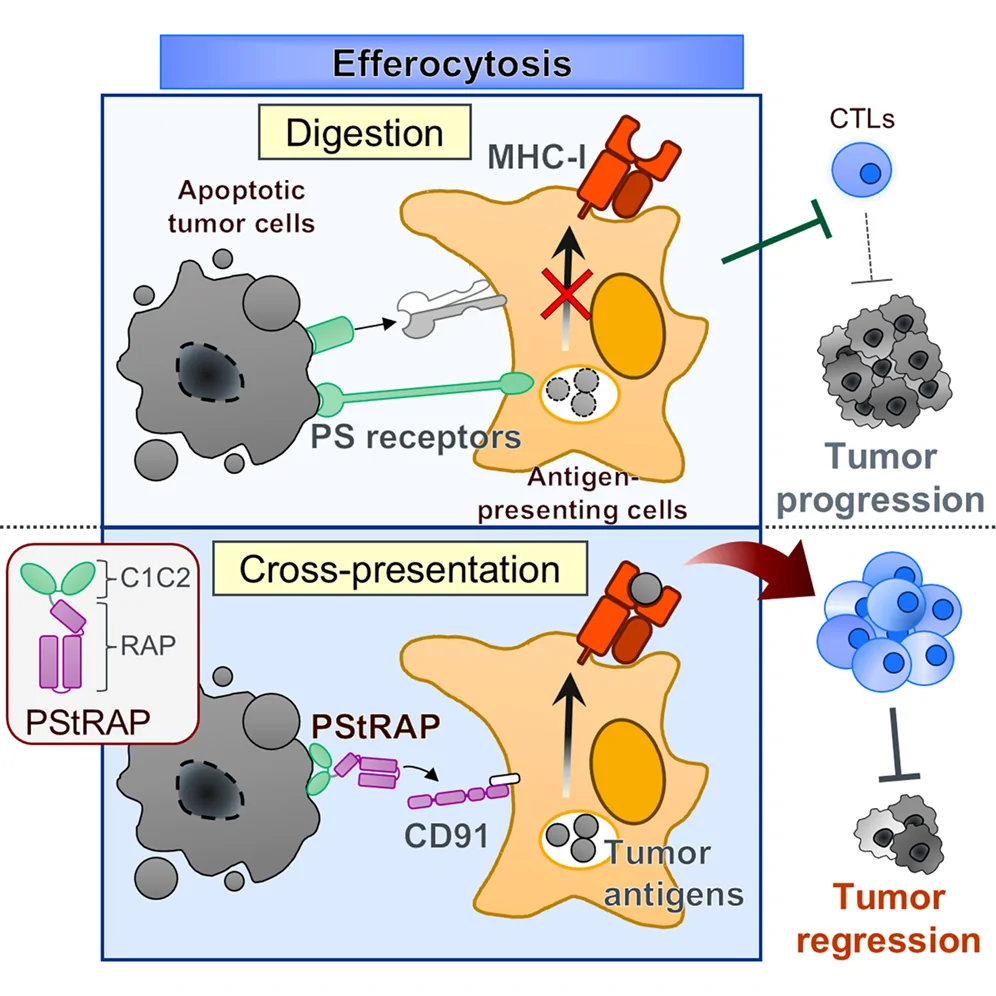

## Teaser

Mizote and colleagues developed PStRAP, a chimeric protein that bridges phosphatidylserine and CD91 to redirect efferocytosis. PStRAP enhances phagocytosis, promotes T-cell activation, and converts an immunosuppressive process into an antitumor immune response, resulting in increased the efficacy of cytotoxic treatments.

## Introduction

Tumor cells escape from immune responses misappropriating various systems equipped for normal cells to be protected from autoimmune responses and maintain homeostasis.^1^ One of the pivotal mechanisms used for avoiding autoimmune responses is rapidly disposing apoptotic cells by phagocytosis, which is termed efferocytosis.^2^ Exposure of phosphatidylserine (PS) to the outer membrane leaflet of apoptotic cells is known not only to be an evolutionarily conserved pathway to prompt efferocytosis^3^ but also can be considered to be an immune checkpoint.^4^ Milk fat globule-epidermal growth factor-factor 8 (MFG-E8) is one of the PS-related molecules that is involved in efferocytosis.^5^ MFG-E8 is a secreted molecule that bridges PS exposed on apoptotic cells and α_v_β_3/5_ integrin on phagocytes. It prompts the rapid engulfment of the apoptotic cells by phagocytes, thereby inducing immune suppression in both normal and cancer cells.^6^^,^^7^

We have been trying to develop immunotherapy against cancer focusing on the PS/MFG-E8 pathway. In our previous study, immunological evasion of the tumor cells can be prevented via blocking the binding of MFG-E8 to integrins using an antibody targeting the region with RGD motif of the MFG-E8.^8^ Our findings suggested that treatment using anti-MFG-E8 Ab with the fragment crystallizable (Fc) region can switch the pathway of efferocytosis from an immunosuppressive one through PS/MFG-E8/integrins to the immunostimulatory one through PS/MFG-E8/antibody/Fcγ receptors. Based on this proofed concept of the treatment, we initiated the study to use MFG-E8 itself combined with receptor-associated protein (RAP) instead of using anti-MFG-E8 Ab to access directly to PS. RAP is also known as LRPAP1, which is a chaperone protein in the endoplasmic reticulum (ER) and firmly binds to CD91.^9^ CD91 is also known as low-density lipoprotein receptor-related protein 1 (LRP1) that acts as an endocytic receptor for various ligands including heat shock proteins (HSPs), such as gp96, hsp70, and calreticulin (CRT). When tumor cells undergo apoptosis with CRT externalized, only the apoptotic tumor cells with such condition are engulfed via CD91. As a result of these events, tumor antigens are presented on dendritic cells (DCs) and induce adaptive immune responses.^10^^,^^11^^,^^12^^,^^13^ We have chosen CD91 pathway instead of Fcγ receptors in this study, since some Fcγ receptors, such as FcγRIIB, do not necessarily induce activation of immune responses.^14^

We have shown that switching the pathway of efferocytosis using a chimeric protein, consisting of PS-binding domain of MFG-E8 and RAP, can induce potent antitumor immune responses when combined with various types of cancer treatment that cause apoptosis of the tumor cells.

## Results

### Design and function of PStRAP

**Figure 1:**
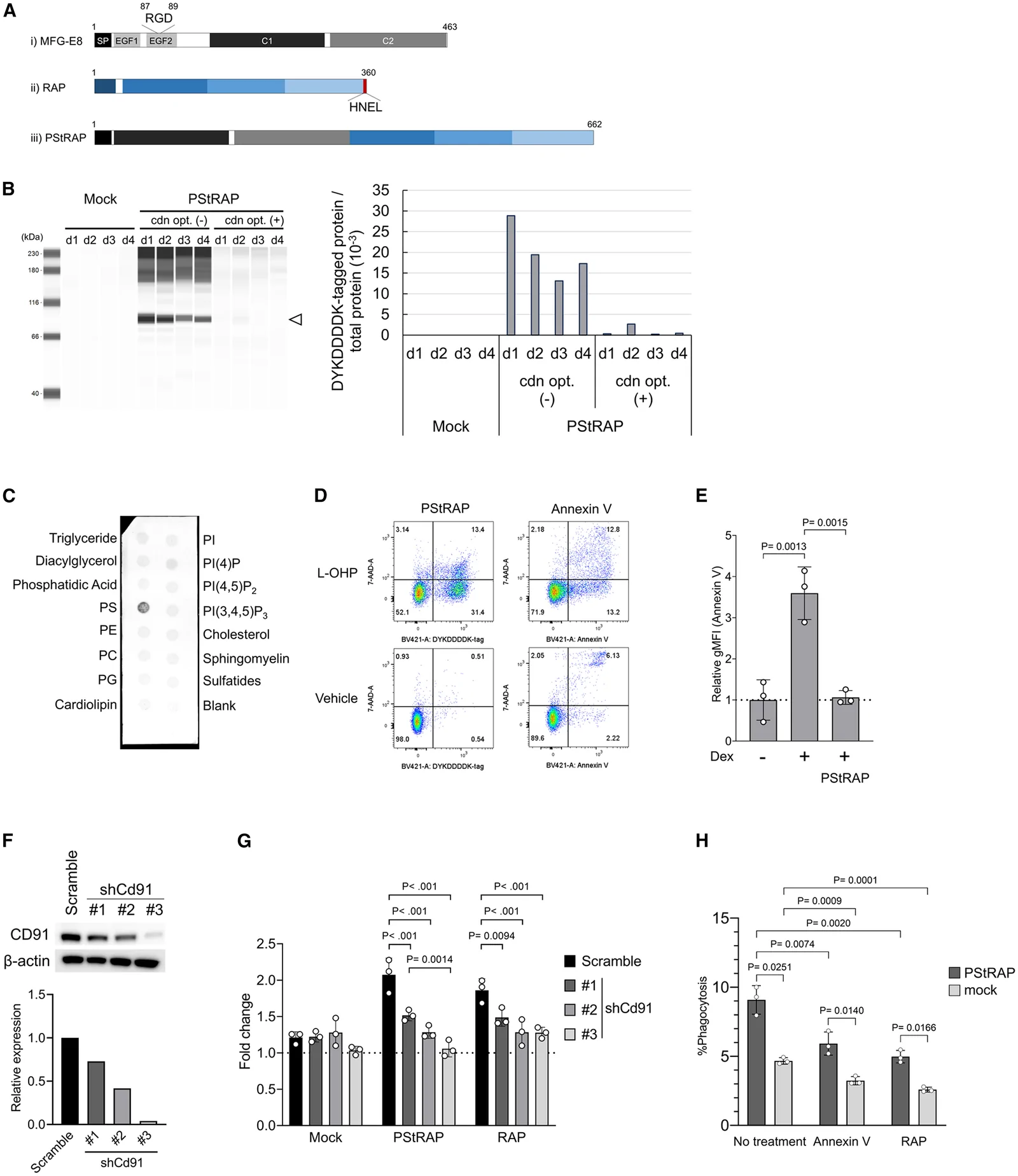
Structure and function of PStRAP (A) The structures of MFG-E8 (i), RAP (ii), and PStRAP (iii) are depicted. RAP has tetrapeptide sequence HNEL at the C terminus for retaining in the ER, and PStRAP replaces this sequence to DYKDDDDK tag. Full-length sequence of PStRAP is shown in Figure S1A. SP, signal peptide. (B) PStRAP was detected using the capillary electrophoresis immunoblotting with anti-DYKDDDDK tag antibody from the culture supernatants of murine primary hepatocytes after mRNA transfection. Gel-like image view of anti-DYKDDDDK tag antibody responses (left). Arrowhead indicates PStRAP with a predicted size of 76.4 kDa. The amounts of DYKDDDDK-tagged protein normalized to the total protein content (right). d, day; cdn opt, codon optimization. (C) Membrane lipid array was performed on the culture supernatants from the murine primary hepatocytes one day after mRNA transfection. PI, phosphatidylinositol; PE, phosphatidylethanolamine; PC, phosphatidylcholine; PG, phosphatidylglycerol. (D) Oxaliplatin (L-OHP; 500 μM) or vehicle-pretreated MCA205 cells were stained with PStRAP and BV421-conjugated anti-DYKDDDDK tag antibody or BV421-conjugated annexin V and counter stained with 7-AAD. (E) Thymocytes pretreated with dexamethasone (Dex; 10 μM) were stained with BV421-conjugated annexin V in the presence of PStRAP (*n* = 3/group). Relative geometric mean fluorescent intensity (gMFI) based on the Dex untreated group is shown. The gating strategy, individual plots, and gMFI values are shown in Figure S2. Error bars show mean (SD). Significance differences were determined using one-way ANOVA followed by post hoc Tukey’s test. (F) CD91 expression level in RAW264.7 cell sublines transfected with shRNA was detected by immunoblotting (top). Pictures of the uncropped membrane are shown in Figure S3. The relative optical density was calculated using the optical density of the scramble shRNA transfectant as a reference (bottom). (G) The luciferase secreted upon NF-κB activation was measured in the culture supernatants from the RAW264.7 cell sublines 1 hour after stimulation with PStRAP or recombinant RAP protein (*n* = 3/group). The fold change was calculated using the luciferase level in the culture supernatants without stimulation as a reference. Error bars show mean (SD). Significance differences were determined using two-way ANOVA followed by post hoc Tukey’s test. (H) RAW264.7 was cultured with CypHer5E-labeled thymocytes that had been pretreated with Dex (10 μM) for 4 h, in conditioning medium containing PStRAP- or mock-supernatant for 2 h. For the competitive assay, either annexin V or RAP protein was added to the medium during co-culturing. The CypHer5E-positive RAW264.7 cells were detected using flow cytometer, and the phagocytosis rate was calculated (*n* = 3/group). The gating strategy and individual plots are shown in Figure S4. Error bars show mean (SD). Significance differences were determined using an unpaired Welch’s *t* test.

We designed a chimeric protein possessing MFG-E8-derived PS-binding and RAP-derived CD91-binding domain, termed PStRAP (PS-targeted RAP) (Figures 1A and S1A). The MFG-E8-derived PS-binding domain contains not only C2 segment, which binds to PS in a calcium ion-independent manner,^15^^,^^16^ but also C1 segment. Recognizing apoptotic cells requires both the C1 and C2 segments of F5/8-type C domain of MFG-E8 in mouse.^5^ The RAP-derived CD91-binding domain contains three segments (D1, D2, and D3) of RAP but not the tetrapeptide sequence HNEL at C terminus required to retain within the ER (Figure 1A(ii)).^17^ The D3 domain primarily binds to CD91 and mediates endocytosis. However, the D1 and D2 domains also bind to CD91.^18^ Plasmids were generated to express and utilize the chimeric protein. The protein was expressed by gene transfer of the plasmid or mRNAs that were synthesized by *in vitro* transcription (IVT) (Figure 1B).

The PStRAP protein was secreted from murine primary hepatocytes through transfecting the synthesized PStRAP mRNA. The PStRAP expression was detected in cell culture supernatant using a DYKDDDDK tag added to C terminus as a marker. We produced template DNA for IVT with the codons theoretically optimized (21.2% base substitution) to increase the protein expression level and to suppress dsRNA production. However, the expression level of the modified template DNA was substantially lower than that of the original sequence (Figures 1B and S1A). Therefore, we used template DNA with the original sequence in this study.

Membrane lipid array was performed on the culture supernatants obtained from murine primary hepatocytes one day after mRNA transfection to examine the specificity of the chimeric protein. The secreted PStRAP had bound to PS but not to other membrane lipids (Figure 1C). PStRAP was able to bind to the apoptotic MCA205 (fibrosarcoma) cells treated with oxaliplatin (L-OHP; 500 μM), but not to the viable cells (Figure 1D). Furthermore, PStRAP showed competitive inhibition against annexin V binding to PS of apoptotic thymocytes (Figures 1E and S2). These results indicate that PStRAP expressed in our system was capable of binding to PS on the apoptotic cells with certain specificity.

We also examined the function of PStRAP using murine macrophage cell line, RAW264.7, reported to express CD91, which activates NF-κB upon binding to several HSPs, such as gp96, hsp70, and CRT.^19^ We thoroughly examined the function of PStRAP by producing RAW264.7 cells with different levels of CD91 knockdown using three types of shRNA (Figures 1F and S3). Similar to other HSPs, we confirmed that NF-κB was activated with PStRAP and RAP at the levels associated with the levels of CD91 expression (Figure 1G). Furthermore, efferocytosis efficiency was significantly increased with the presence of PStRAP compared with its absence, and the effect was attenuated by the addition of either annexin V or RAP (Figures 1H and S4). These results suggested that PStRAP can serve as a mediator for efferocytosis through recognizing PS and CD91.

### PStRAP induced adaptive antitumor immune responses

**Figure 2:**
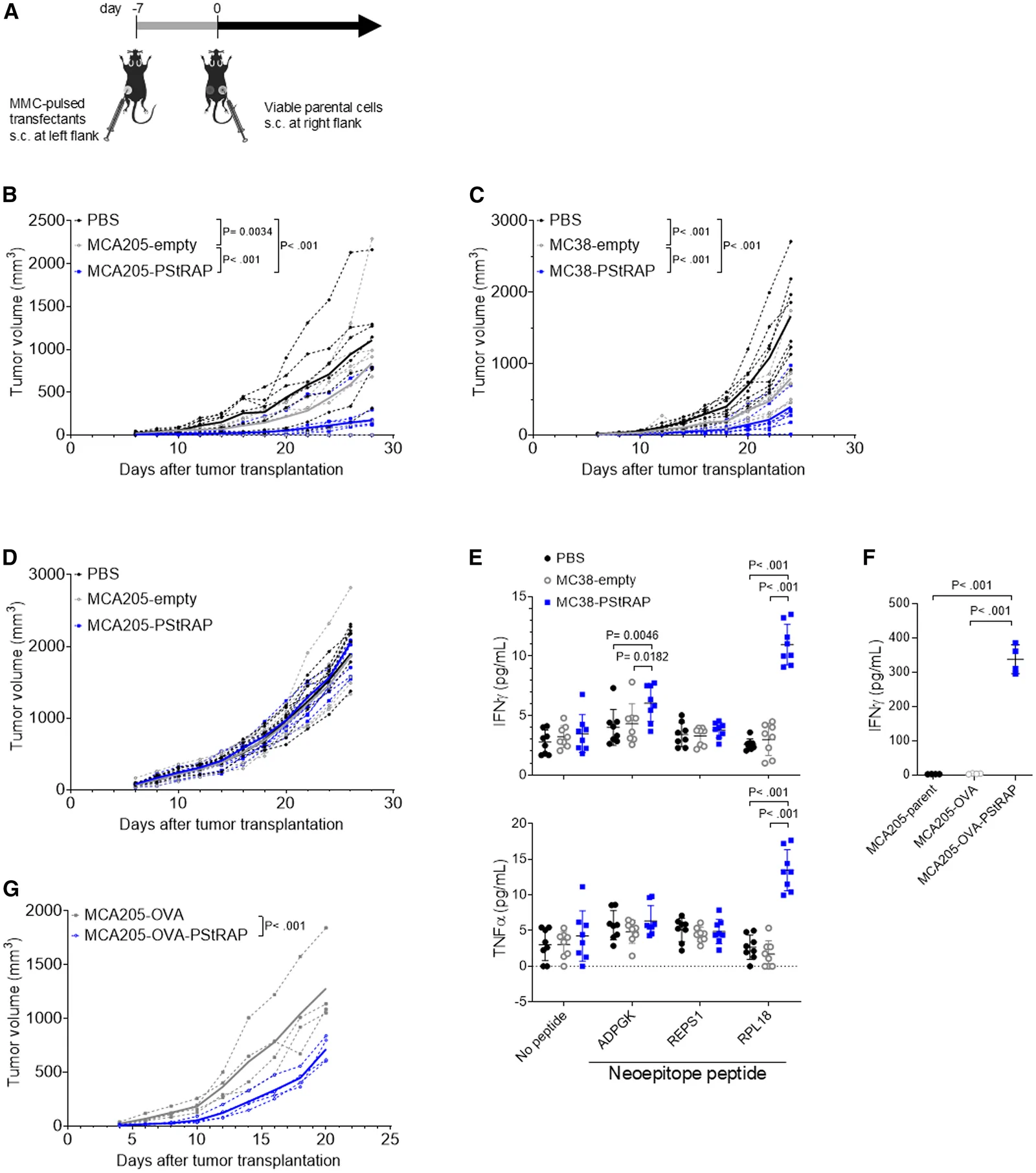
PStRAP enhanced cross-presentation and induced adaptive antitumor immune responses (A–D) Each MMC-pulsed transfectant (5 × 10^5^ cells) or PBS was subcutaneously inoculated into the left flank 7 days before subcutaneous transplantation of viable MCA205 (5 × 10^5^ cells) (B and D) or MC38 (3 × 10^5^ cells) (C) cells at the right flank of C57BL/6 mice (B and C) or nude mice (D), and then the tumor volumes were measured every other day (*n* = 8/group). Significance differences were determined using two-way ANOVA followed by post hoc Tukey’s test. (E) The splenocytes were harvested from MC38-transplanted mice on day 26, as shown in (C). The splenocytes cultured with 10 μM of indicated peptide or no peptide for three days, the culture supernatants were collected, and the amounts of IFNγ and TNFα were measured using ELISA. Error bars show mean (SD). Significance differences were determined using two-way ANOVA followed by post hoc Tukey’s test. (F) APCs were prepared from tumor-draining subiliac lymph node of mice (*n* = 4/group) one day after MMC-pulsed parental MCA205, MCA205-OVA, or MCA205-OVA-PStRAP cells (2 × 10^6^ cells) subcutaneously inoculated at the right flank. CTLs that were purified from splenocytes of OT-I mice and the APCs were cocultured for three days followed by collection of the culture supernatants and measuring the amounts of IFNγ using ELISA. Error bars show mean (SD). Significance differences were determined using one-way ANOVA followed by post hoc Tukey’s test. (G) MCA205-OVA or MCA205-OVA-PStRAP (5 × 10^5^ cells) were subcutaneously inoculated into the left flank of the OT-I mice. Viable MCA205 (5 × 10^5^ cells) cells were subcutaneously transplanted into the right flank seven days later, and then tumor volumes were measured every other day (*n* = 4/group). Significance differences were determined using two-way ANOVA.

We evaluated whether PStRAP induces adaptive antitumor immune responses *in vivo* as designed by immunizing tumor cell lines stably expressing PStRAP that was transfected with PStRAP DNA without the DYKDDDDK tag using a retrovirus-expressing vector. Mitomycin C (MMC)-pulsed transfectants were subcutaneously inoculated as an immunogen at the flank of mice. Under this treatment condition, although tumor cells are viable at the time of inoculation (3 h), more than 70% undergo apoptosis by 24 h, and nearly all cells were dead by 48 h (Figure S5A). The relative expression level of 111 soluble proteins secreted into the culture supernatant was determined. Although 13 secreted factors were detected in viable cells, there was no change related to PStRAP expression. The MMC pulse caused some factors to either remain the same or decrease 24 h later; however, none of the factors increased as a result of the treatment. Similar profiles of soluble factors were shown between PStRAP-expressing and non-expressing MMC-pulsed cells (Figure S5B). Viable parental cells were subcutaneously transplanted at the opposite flank 7 days later, and then the resulting antitumor immune responses were evaluated (Figure 2A). Consistent with the results of our previous study,^7^ the tumor growth in the mice inoculated with 5 × 10^5^ cells of MMC-pulsed tumor cells were significantly suppressed compared with that in the mice inoculated with vehicle. The effect of tumor growth suppression was significantly enhanced by the inoculation of same number of MCA205 cells expressing PStRAP (Figure 2B). The significant enhancement of the growth suppression was also observed in a different tumor model using another syngeneic tumor cell line model (colon adenocarcinoma MC38) as well (Figure 2C).

Tumor growth suppression was not observed in the nude mice deficient in adaptive immunity, indicating that the tumor growth suppression was associated with the induction of adaptive antitumor immune responses due to the expression of PStRAP from dying tumor cells (Figure 2D). This interpretation was further supported by the observation that the splenocytes harvested from the mice inoculated with MC38-PStRAP secreted interferon gamma (IFNγ) and tumor necrosis factor alpha (TNFα) *in vitro* upon restimulation with peptides of mutation-derived neoepitopes (Figure 2E). These peptides are recognized by antigen-specific cytotoxic T-lymphocytes (CTLs) in the response to the MC38.^20^^,^^21^ We further analyzed the effects of PStRAP on the cross-presentation efficiency by antigen-presenting cells (APCs) using ovalbumin (OVA)-expressing MCA205 and OT-I mice, which have a T-cell receptor that specifically recognizes the OVA_257-264_ peptide bound to H-2K^b^.^22^ Mice were immunized with MCA205 transfectants pulsed with MMC, and the APCs were harvested from the tumor-draining lymph nodes one day later. The APCs were cocultured with CD8^+^ cells from OT-I mice for three days. The amounts of IFNγ in the culture supernatants were detected only when PStRAP-expressing cells were inoculated (Figure 2F).

We also confirmed that PStRAP can induce adaptive immune responses through cell death caused by the endogenous immune response rather than by the drug. Instead of MMC-pulsed MCA205 cells, viable OVA-expressing MCA205 transfectants were subcutaneously inoculated at the flank of OT-I mice. Viable parental cells were subcutaneously transplanted at the opposite flank 7 days later. As OT-I mice harbor potent OVA-specific CTLs, the OVA-expressing cells are efficiently killed and completely rejected *in vivo* regardless of PStRAP expression in this tumor model. Therefore, in this setting, the OVA-expressing cells are assumed to primarily serve as a source of antigen during or following immune-mediated cell death, rather than serve as antigen-presenting live cells. Under this condition, tumor growth of parental cells was significantly suppressed in PStRAP-expressing group (Figure 2G). These results demonstrate that PStRAP efficiently induces adaptive immune responses in association with cell death, consistent with its role in enhancing immune priming from dying cells.

### PStRAP requires recognition of both PS and CD91 in order to establish adaptive antitumor immune responses

**Figure 3:**
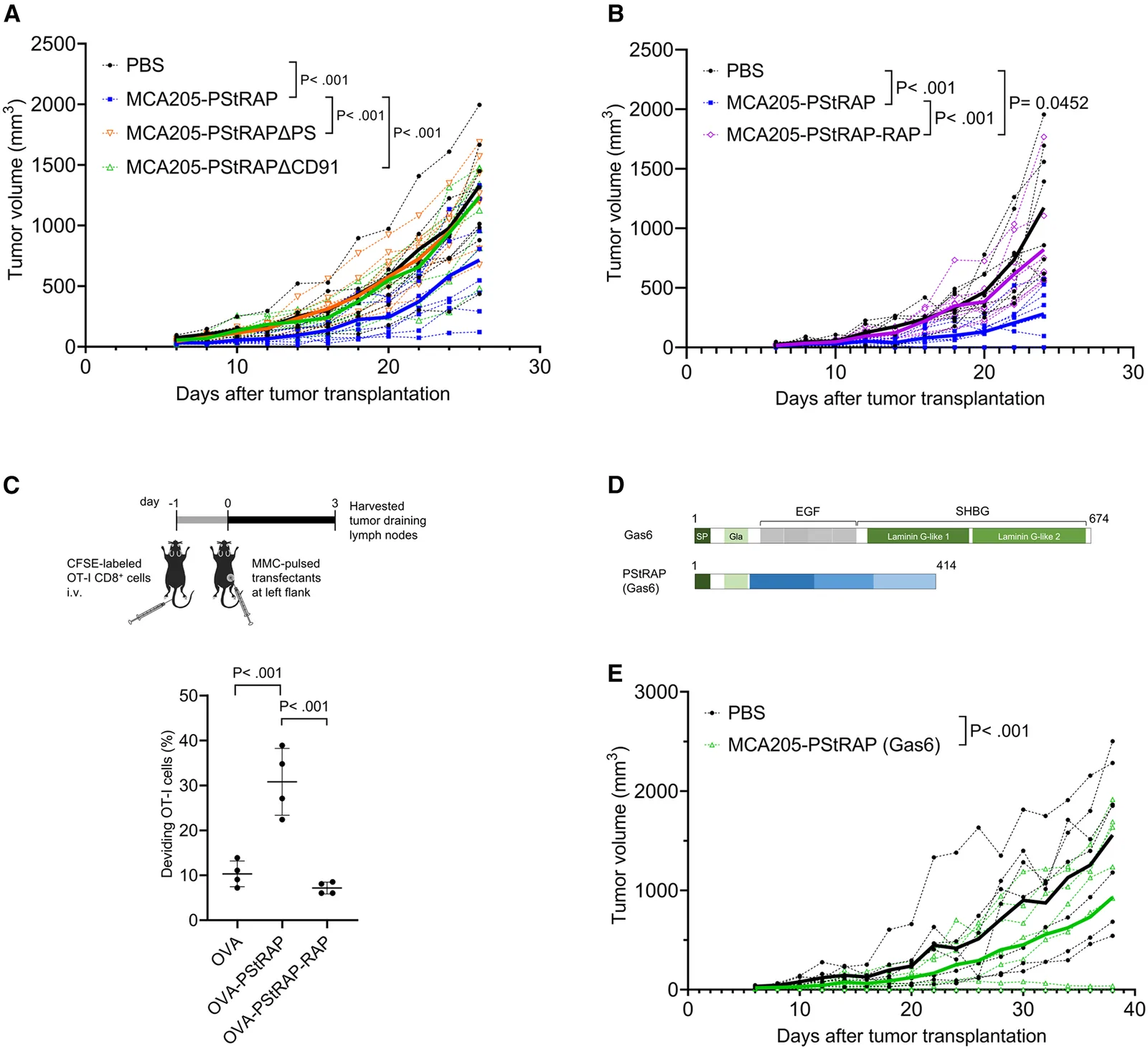
PS and CD91 recognition enable for PStRAP-induced adaptive immune responses (A, B, and E) MMC-pulsed MCA205-PStRAP, its mutant (ΔPS or ΔCD91) (A), RAP co-expressing (B), or PStRAP (Gas6) (E) transfectants (5 × 10^5^ cells), or PBS were subcutaneously inoculated into the left flank of the mice. Viable MCA205 (5 × 10^5^ cells) cells were subcutaneously transplanted into the right flank seven days later, and then tumor volumes were measured every other day (*n* = 8/group). Significance differences were determined using two-way ANOVA followed by post hoc Tukey’s test. (C) The effect of RAP on T-cell proliferation *in vivo* was examined. Wild-type C57BL/6 mice were adoptively transferred with CFSE-labeled OT-I cells, 1 day before immunization with MMC-pulsed MCA205-OVA with or without PStRAP and RAP expression. After three days, the draining subiliac lymph nodes were harvested and stained for TCR-Vβ5 and CD8 expression. The percentages of dividing OT-I cells are shown (*n* = 4/group). Error bars show mean (SD). Significance differences were determined using one-way ANOVA followed by post hoc Tukey’s test. The gating strategy and individual histograms are shown in Figure S8. (D) The structures of Gas6 (top) and PStRAP (Gas6) (bottom) are depicted. SP, signal peptide; SHBG, sex hormone-binding globulin.

After confirming PStRAPΔPS and PStRAPΔCD91 have no binding capabilities to their respective targets (Figure S6), we then examined the antitumor effects of inoculating with the dying tumor cells expressing these mutants. No significant antitumor effects were observed in the mice inoculated with dying MCA205 cells expressing these mutants (Figure 3A). This contrasts with the tumor behaviors observed in the mice inoculated with the dying MCA205 cells expressing PStRAP. Interestingly, the tumors in the mice treated either with PStRAPΔPS or PStRAPΔCD91 showed more rapid growth compared with the tumors in the mice inoculated with the cells without PStRAP expression (Figures 2B and 3A). The tumor growth suppression effect induced by PStRAP attenuated when both PStRAP and RAP were expressed in MCA205 cells used as an immunogen (Figures 3B and S7). To evaluate the cross-presentation efficacy *in vivo*, CFSE-labeled OT-I CD8^+^ T-cells were adoptively transferred to wild-type mice. One day later, OVA-expressed MCA205 cells with or without PStRAP, or with both PStRAP and RAP, were inoculated to flanks of mice. Three days later, the draining lymph nodes were harvested and detected for dividing OT-I cells. The number of dividing OT-I cells increased in the presence of PStRAP compared with its absence, and this effect was eliminated by the presence of RAP (Figures 3C and S8).

It is conceivable that different types of PStRAP can be constructed using the other PS-binding molecules that are functionally similar to the C1C2 domain of MFG-E8. Thus, we incorporated Gla domain of growth arrest-specific 6 (Gas6) and T-cell immunoglobulin mucin protein 4 (Tim4), which have potent PS-binding capability, into PStRAP, respectively (PStRAP (Gas6) and PStRAP (Tim4)) (Figures 3D and S9A). Similar to MFG-E8, Gas6 is a secreted protein that opsonizes apoptotic cells.^23^^,^^24^ PStRAP (Gas6) also induced antitumor effects similar to that of PStRAP using the C1C2 domain (Figure 3E). Although MFG-E8 and Tim4 are expressed by different subsets of macrophages, they both tightly bind to PS on apoptotic cells and enhance efferocytosis by phagocytes.^25^^,^^26^ Furthermore, mice with these genes knocked out age-dependently develop an autoimmune disease.^27^ Nevertheless, PStRAP (Tim4) expression was not associated with significant antitumor effects (Figure S9B). The strategy of capturing PS is effective, but the capture molecules should be selected with caution. These results confirm that the significant antitumor effects were induced by PStRAP functions that could bridge between PS and CD91 and promote the induction of tumor antigen-specific CTLs.

### Systemic expressing PStRAP via intravenous injection of LNP/mRNA enhanced antitumor effect of cytotoxic drug

**Figure 4:**
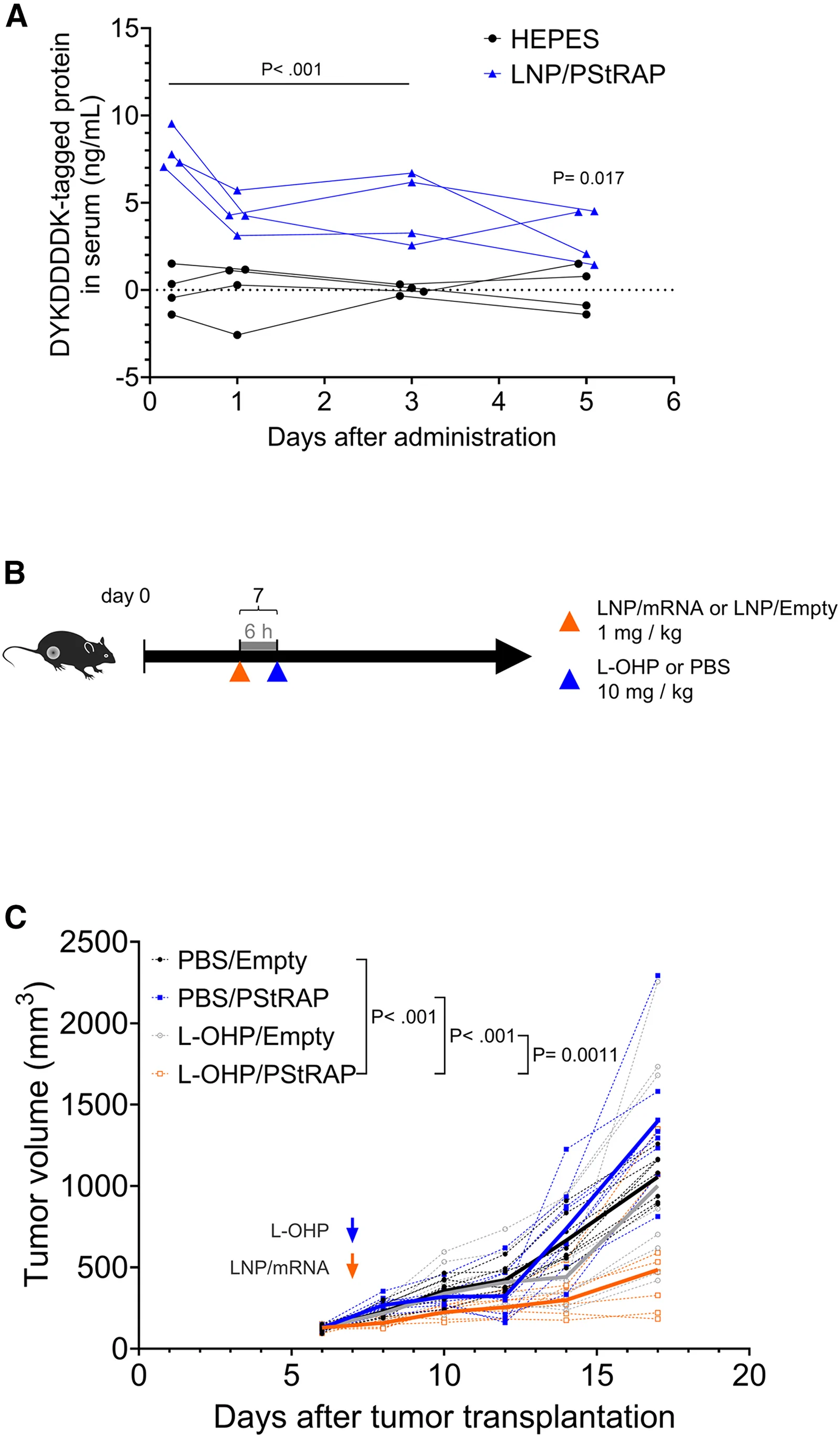
Anticancer therapeutic effects of PStRAP systemically expressed via LNP/mRNA inoculation (A) Blood was collected from the mice at the indicated time points after intravenous LNP/mRNA (1 mg/kg) or HEPES injection. The amounts of PStRAP in serum were measured via detecting DYKDDDDK-tagged proteins. Significance differences were determined using an unpaired Welch’s *t* test at each time point. (B and C) MCA205-bearing mice were intravenously injected on day 7 with LNPs containing mRNA encoding PStRAP (1 mg/kg) or empty LNP 6 h prior to intraperitoneally administrating L-OHP (10 mg/kg) or PBS on the same day. Significance differences were determined using two-way ANOVA followed by post hoc Tukey’s test.

To confirm the effectiveness of PStRAP treatment in a therapeutic model, mRNA of PStRAP encapsulated in lipid nanoparticle (LNP/PStRAP-mRNA) was administered intravenously. Intravenously administering LNP/mRNA is expected to be mainly delivered to the liver and translated into protein and then released in the systemic circulation. To confirm the *in vivo* delivery of this system, firefly luciferase (FLuc)-mRNA encapsulated in LNPs (LNP/Fluc-mRNA) were injected into tail veins of the mice. Several organs were harvested 6 or 24 h after administration, and the luminescence intensities were measured. The luminescence was detected in only the liver and 6 h after administration, and it was detectable but at decreased levels 24 h after administration (Figure S10A). PStRAP protein was detected in the serum 6 h after LNP/PStRAP-mRNA administration and remained detectable until five days after administration but at decreased levels (Figure 4A). The levels of alanine aminotransferase (ALT) and aspartate aminotransferase (AST), and the weight of the administrated mice did not differ from those of the vehicle-administrated mice (Figures S10B and S10C). These results suggest that intravenously injecting this dose of LNP/PStRAP-mRNA showed no serious toxicity and did not induce liver injury (Figure S10C). To investigate the antitumor effects of LNP/PStRAP-mRNA treatment in an established tumor model, mice bearing day 7 tumors were treated with LNP/PStRAP-mRNA combined with L-OHP (Figure 4B). L-OHP induced apoptosis in MCA205, which was recognized by PStRAP *in vitro* (Figure 1D). Although administrating L-OHP alone or LNP/PStRAP-mRNA alone did not affect tumor growth compared with that in the vehicle-administrated group, combined L-OHP and LNP/PStRAP-mRNA treatment significantly suppressed tumor growth (Figure 4C). These results suggest that LNP/PStRAP-mRNA treatment can be used to enhance antitumor effects when combining with the treatment that induce apoptosis in established tumors.

## Discussion

It has been shown by us and other investigators that antitumor effects can be elicited by the treatment with antibodies against PS-bridging molecules including MFG-E8 or β2-glycoprotein 1.^8^^,^^28^ Since these antibodies have been used to block the functions related to PS by targeting the PS-bridging molecules that spontaneously bind to PS, various other PS-bridging molecules and PS receptors can still bind to PS and modulate immune functions.^4^^,^^29^ One of the strategies to improve this situation is to develop treatments using molecules with high binding affinities to PS to impede the binding of other molecules. In murine neuroblastoma model where viable tumor cells constitutively expose PS on their surface to suppress T-cell functions, the masking of PS with annexin V can induce antitumor immune responses.^30^ However, annexin V requires a higher percentage of PS on the external plasma membrane for binding compared with MFG-E8.^31^^,^^32^ As such, we have initiated the study using C1C2 domain of MFG-E8, instead of anti-MFG-E8 antibody, to directly access the PS of apoptotic cells. We designed PStRAP, a chimeric protein, to modulate the immunoregulatory PS-related pathway with the C1C2 domain of MFG-E8. Using PStRAP, we have successfully shown in two different syngeneic tumor cell line models (fibrosarcoma MCA205 and colon adenocarcinoma MC38) that the tumor growth in the mice inoculated with PStRAP-expressing dying tumor cells was significantly suppressed when compared with that in the mice inoculated with the dying tumor cells transfected with empty vector (Figures 2B and 2C). Since this tumor growth suppression was shown to be associated with the promotion of tumor-specific immunity (Figures 2D–2F), the concept of our strategy appears to be proven.

Interestingly, the tumors in the mice treated either with PStRAPΔPS or PStRAPΔCD91 showed more rapid growth compared with the tumors in the mice treated with cells without PStRAP expression (Figures 2B and 3A). The PStRAP lacking CD91 binding ability (PStRAPΔCD91) might play the immunoregulatory roles similar to the MFG-E8 mutant lacking integrin-binding capacity (e.g., C1C2 domain only, RGD motif replaced with RGE), which prevents the engulfment of apoptotic cells and suppresses subsequent antigen cross-presentation as shown in the study reported by the other group.^6^ Regarding PStRAP lacking PS-binding ability (PStRAPΔPS), it might behave similarly to the RAP, which inhibited usual efferocytosis by RAW264.7 cells *in vitro* (Figure 1H) and could suppress CD91-dependent pathways and prevent the induction of adaptive immune responses.^33^ These results support that it is necessary to express PStRAP in a complete form to induce positive antitumor immune responses.

It is conceivable that alternative PS-binding molecules could be used to construct PStRAP variants functionally similar to the C1C2 domain of MFG-E8. One candidate molecule is Gas6, which binds PS via its Gla domain. Although full-length Gas6 also contains a sex hormone-binding globulin (SHBG)-like domain that engages three receptor tyrosine kinases of the TAM (Tyro3, Axl, and Mertk) family, our construct utilized only the Gla domain and therefore lacks TAM receptor-binding capacity (Figure 3D).^24^^,^^34^ Gas6 binds to TAM receptors on APCs during efferocytosis to promote phagocytosis and transmit immunosuppressive signals that prevent autoimmune responses.^35^^,^^36^ Another candidate molecule is Tim4, which acts as a PS receptor and cooperates with integrins to promote efferocytosis.^37^ In cancer situations, Tim4 is reported to be expressed on non-small cell lung cancer and interacts with the α_v_β_3_ integrin to promoted tumor growth.^38^ Thus, we incorporated Tim4, a molecule with potent PS-binding capability, into PStRAP (PStRAP (Tim4)) (Figure S9A). Although Tim4 binds to PS similarly to MFG-E8 and Gas6, PStRAP (Tim4) was unable to induce significant antitumor effects (Figure S9B). Notably, the PS-binding region of Tim4 resides within an IgV-like domain that also contains an RGD motif implicated in integrin interactions (Figure S9A).^39^ It is possible that integrin engagement or structural constraints within the Tim4 domain alter the orientation or signaling context of PS presentation, thereby limiting its capacity to promote immunogenic antigen processing. Therefore, PS-binding molecules with other functions but located in the same domain may not be suitable for our strategy.

The data shown in our current study suggest that PStRAP promotes the uptake of PS-exposing dying cells, leading to antigen processing and cross-presentation by recipient APCs. However, it is possible that extracellular vesicles (EVs) that express PS have roles in our system, since EVs have been shown to be involved in various types of immunological stimulation and regulation.^40^^,^^41^^,^^42^^,^^43^ DC-derived EVs have been reported to express peptide-MHC complexes together with co-stimulatory molecules and can directly activate T-cells.^44^ In contrast, tumor-derived EVs have been reported to induce T cell dysfunction by modulating the tumor microenvironment.^45^^,^^46^ Furthermore, they may also serve as a source of antigen for uptake and processing by recipient APCs.^47^ Although we cannot exclude a contribution of EV-associated antigen to the results of the current study, it appears unlikely that EVs alone account for the observed effects, since similar responses were not observed in our experimental setting using the viable cells (Figure 4C). Further studies might lead to a better understanding of the roles of EVs in our system.

CD91 is a highly endocytic and rapidly recycling scavenger receptor that constitutively undergoes ligand-induced internalization followed by efficient return to the plasma membrane.^48^^,^^49^ Although RAP is a well-characterized CD91 ligand that can induce transient receptor internalization,^50^ due to the rapid recycling kinetics of CD91, such internalization does not necessarily lead to sustained surface depletion.^49^^,^^51^ In the present study, PStRAP may exist in both soluble and PS-bound forms depending on the availability of exposed PS. In the context of apoptotic tumor cells, PS exposure likely promotes local enrichment of PStRAP at the dying cell surface, thereby facilitating spatially restricted CD91 engagement. Under these conditions, PStRAP is less likely to function as a systemic excess ligand causing global receptor downregulation. Rather than quantitatively altering CD91 surface levels, our data support a model in which PStRAP qualitatively redirects CD91-mediated processing of dying tumor cells toward an immunogenic outcome. Importantly, we did not observe phenotypic evidence of impaired apoptotic cell clearance *in vivo* under the experimental conditions used. Together, these findings support a mechanism in which PStRAP enhances the immunogenic context of CD91 engagement without disrupting physiological receptor availability. Future studies examining the kinetics of CD91 trafficking in response to PStRAP will further refine this mechanistic framework.

In this report, we developed a chimeric protein termed PStRAP to modulate not only the immunoregulatory PS-related pathway but also the immunostimulatory CD91-related pathway.^52^ CD91 is expressed on professional APCs, including DCs and macrophages, where it mediates endocytic uptake of extracellular ligands and contributes to antigen cross-presentation.^10^^,^^53^ CD91-expressing DCs have been identified as a subset of professional APCs capable of enhancing adaptive immune responses to CD91-targeted antigens.^54^ In the context of tumor immunity, CD91-dependent interactions have been shown to be required for the establishment of effective antitumor immune responses.^33^ Additionally, CD91-gp96 signaling has been demonstrated to confer cross-priming capacity to multiple APC subsets during immune responses to emerging tumors.^55^ Within this established framework, our findings support a model in which PStRAP enhances the immunogenic processing of apoptotic tumor cells by promoting CD91-mediated antigen acquisition at the dying cell-APC interface. Since the results of cytokine profile analysis for dying tumor cells did not reveal apparent or consistent changes in inflammatory mediator production in the presence or absence of PStRAP (Figure S5B), it would be difficult to speculate the mechanism of the PStRAP-related antitumor effects would be primarily driven by secondary soluble factors. In addition to the examination of enhanced apoptotic cell uptake, we evaluated cross-presentation function using antigen-specific CD8^+^ T-cells. The presence of PStRAP significantly increased CD8^+^ T-cell proliferation, indicating enhanced cross-presentation efficiency (Figures 1H and 3C). These data indicate that PStRAP does not primarily amplify adaptive immunity through nonspecific inflammatory activation but rather augments receptor-mediated antigen acquisition. Given that apoptotic cells alone induce a baseline adaptive response (Figures 2B and 2C), the enhanced uptake observed in the presence of PStRAP supports a model in which PStRAP qualitatively redirects or amplifies CD91-dependent antigen processing, thereby significantly strengthening antitumor immunity without broadly reshaping the inflammatory milieu of dying tumor cells.

The mode of tumor cell death that causes subsequent adaptive antitumor immune responses has been defined as immunogenic cell death (ICD), and several treatments for cancer have been shown to induce ICD.^56^ For example, cell death involving cell surface exposure of CRT is considered a hallmark of ICD. However, multiple types of cancer do not respond to chemotherapeutic agents that induce ICD. In contrast, PStRAP was able to promote potent adaptive antitumor immune responses even when cell death was induced by MMC, which is a representative non-ICD inducer (Figures 2B and 2C). PStRAP also was able to enhance the adaptive immune responses resulting from tumor cell death caused by CTLs occurring *in vivo* (Figure 2G). This suggests that modulation of CD91-mediated uptake represents a strategy to amplify antitumor immunity independent of the intrinsic immunogenicity of tumor cell death. In addition, recent reports showed that PS exposure to the outer membrane leaflet of cell surface also occurs in programmed necrosis, such as necroptosis and pyroptosis.^57^^,^^58^ PStRAP can induce the antitumor immune responses independent of the mode of cell death and may be useful when combined with various therapies. They include not only conventional chemotherapy and radiotherapy but also other types of treatments, such as CAR-T therapy and oncolytic virus therapy.

In current study, when mRNA of PStRAP encapsulated in lipid nanoparticle (LNP/PStRAP-mRNA) was intravenously administered, mice bearing day 7 established tumors were successfully treated with LNP/PStRAP-mRNA combined with the chemotherapy (Figure 4C). These results suggest that also systemically administrating PStRAP protein can be an effective therapy when combined with other types of treatments that induce tumor apoptosis including existing immune checkpoint inhibitors.

Collectively, our findings demonstrate that manipulating the efferocytosis pathway may lead to the activation of potent adaptive antitumor immune responses, and PStRAP may serve as a potentiator for existing various types of cancer therapy.

## Materials and methods

### Mice

The C57BL/6NJ and CAnN.Cg-*Foxn1*^*nu/nu*^/CrlCrlj (nude) mice were obtained from Charles River Laboratories Japan (Kanagawa, Japan). The C57BL/6-Tg (TcraTcrb) 1100Mjb (OT-I) mice were provided by Dr. W.R. Heath (Walter & Eliza Hall Institute of Medical Research, Melbourne, Australia).^22^ All animal experiments were approved by the Animal Care and Use Committee of the Osaka International Cancer Institute (approval numbers: 25030325, 24021909, and 23022116). The experiments were conducted under specific pathogen-free conditions, reproduced more than twice, and representative data were depicted.

### Cells and transfectants

MCA205 (murine fibrosarcoma) and MC38 (murine colon adenocarcinoma) cell lines were provided by Dr. S.A. Rosenberg (National Cancer Institute, Bethesda, MD). These cell lines were authenticated via short tandem repeat profiling using an FTA Sample Collection Kit for Mouse Cell Authentication Service (137-XV, American Type Culture Collection: ATCC, Manassas, VA). RAW264.7 (murine leukemia macrophage) cell lines were purchased from ATCC (TIB-71). Tumor cell lines and primary hepatocytes were cultured in Dulbecco’s modified Eagle’s medium (DMEM), high-glucose, GlutaMAX Supplement (10566, Thermo Fisher Scientific, Waltham, MA) supplemented with 1% penicillin-streptomycin (15140, Thermo Fisher Scientific), and 10% fetal bovine serum (35-079-CV, Corning Inc., Corning, NY) (complete DMEM) at 37°C in a 5% CO_2_ atmosphere.

Primary cells from thymus, spleen, and lymph nodes were cultured in Roswell Park Memorial Institute (RPMI) 1640, GlutaMAX Supplement (61870, Thermo Fisher Scientific) supplemented with 1% penicillin-streptomycin, 50 μM of 2-Mercaptoethanol (21985023, Thermo Fisher Scientific), and 10% fetal bovine serum (complete RPMI) at 37°C in a 5% CO_2_ atmosphere.

OVA-expressing MCA205 cells (MCA205-OVA) were established following a previously reported method.^7^ PStRAP, its mutants, and PA-tagged RAP at the C terminus were expressed via pMXs retroviral transfection (RTV-012, Cell Biolabs, San Diego, CA). Using the information on MFG-E8 and RAP on binding to PS and CD91, respectively, we constructed PStRAP mutants that were functionally defective in binding to PS or CD91. PStRAPΔPS was produced by replacing W333, N334, L335, F388, and G389 in the MFG-E8 region with alanine and was unable to bind to PS.^59^ PStRAPΔCD91 was produced by replacing K293 and K307 in the RAP region with alanine and was unable to bind to CD91 (Figure S1A).^18^

For knockdown of CD91 (GenBank: NM_008512.2), short hairpin RNA (shRNA) expressing lentivirus made from pLV vector was used. Three *Cd91*-targeted shRNAs (#1: AGGCGCCTGTGTGGTCAATAA, #2: CGACAACGACTGCGGAGATAA, or #3: CGGAGTCACTTACATCAATAA) and one scramble shRNA control (CCTAAGGTTAAGTCGCCCTCG) containing lentivirus particles were purchased from VectorBuilder (Chicago, IL), which were infected to RAW264.7 cells. To detect NF-κB activation, pNiFty2-N-Lucia-Blasti (pnf2b-lc, InvivoGen, San Diego, CA) was transfected into shRNA-expressing RAW264.7 cell sublines.

### mRNA synthesis and LNP formulation

DNA synthesis, codon optimization (using GenSmart Optimization version Beta 1.0), cloning, and plasmid preparation were provided by GenScript (Piscataway NJ). The plasmid DNA was linearized by the restriction enzyme, BspQI (#R0712, New England Biolabs, Ipswich, MA). mRNA was synthesized *in vitro* on linearized plasmid, which consisted of a T7 promoter, a 5′-untranslated region (UTR) containing a Kozak sequence, a coding region, a 3′-UTR, and a 120-nt poly(A) tail, using a MEGAscript T7 Transcription Kit (#AM1330, Thermo Fisher Scientific) following the instructions provided by the manufacturer (Figure S1B). Uridine 5′-triphosphate was fully substituted with 5-methoxyuridine-5′-triphosphate (5moU; #*N*-1093, TriLink Biotechnologies, San Diego, CA), and the 5′ capping of the IVT mRNA was cotranscriptionally incorporated using the CleanCap Reagent AG (#*N*-7113-5, TriLink Biotechnologies). IVT mRNA was purified using high-performance liquid chromatography (Akta explorer 100, cytiva, Wilmington, DE) with a CIMmultus Oligo dT18 1 mL Monolithic Column (C12 Linker) (2-μm channels) (#311.1219–2, Sartorius, Göttingen, Germany). The collected fractions were concentrated using an Amicon Ultra-15 centrifugal filter unit (30 kDa) (UFC9030, Merck KGaA, Darmstadt, Germany). The integrity of the IVT mRNAs was analyzed using capillary electrophoresis.

For formulation of mRNA-encapsulated LNPs (LNP/mRNA), NanoAssemblr Benchtop Systems (Precision NanoSystems, Vancouver, Canada) was used as previously described.^60^^,^^61^ Briefly, the lipid mixture with mRNA (or without mRNA for control) was rapidly mixed at an N/P ratio of 6 in citrate buffer (50 mM, pH 3.5). These LNP solutions were ultrafiltered using Amicon Ultra-15 centrifugal filter units (100 kDa) (UFC9100, Merck KGaA) for buffer exchange with 20 mM HEPES (4-(2-hydroxyethyl)piperazine-1-ethanesulfonic acid) 9% sucrose buffer (pH 7.45). LNPs containing ALC-0315, an ionizable cationic lipid also used in the approved SARS-CoV-2 mRNA vaccine,^62^ were employed to deliver mRNA to the liver.

### Hepatocyte purification and transfection

Single-cell hepatocytes were dissociated from fresh mouse liver using a Liver Perfusion Kit (130-128-030, Miltenyi Biotec, Bergisch Gladbach, Germany) and gentleMACS Octo Dissociator (130-134-029, Miltenyi Biotec) according to the instructions provided by the manufacturer. After the hepatocytes were purified, the primary hepatocytes were transfected with mRNA using Lipofectamine MessengerMAX Transfection Reagent (LMRNA, Thermo Fisher Scientific). Culture supernatants were collected daily for 4 days after transfection and cryopreserved until use.

### Western blotting

Whole-cell lysates were prepared using RIPA Lysis Buffer containing a Halt Protease Inhibitor Cocktail (89900 and 87786, Thermo Fisher Scientific). Each cell lysate (1 × 10^5^ cells per lane) was loaded on mPAGE 4–20% Bis-Tris Precast Gels (MP42G15, Merck KGaA) and separated using SDS-PAGE. The proteins in the gel were transferred to an Immobilon-P transfer membrane (IPVH07850, Merck KGaA), and the membrane was stained with LRP1 Recombinant Rabbit Monoclonal Antibody (clone SA0290, 1/1,000 dilution; MA5-31959, Thermo Fisher Scientific), Anti PA tag Rat Monoclonal Antibody (clone NZ-1, 1/1,000 dilution; 012–25863, FUJIFILM Wako, Osaka, Japan), or Anti β-Actin Monoclonal Antibody (clone 6D1, 1/10,000 dilution; 010–27841, FUJIFILM Wako). The membrane was then stained with Peroxidase-conjugated AffiniPureR Alpaca Anti-Rabbit IgG (H + L), Anti-Mouse IgG (H + L), or Donkey Anti-Rat IgG (H + L) (polyclonal, 1/10,000 dilution; 611-035-215, 615-035-214, and 712-035-153, respectively, Jackson ImmunoResearch, Philadelphia, PA). The enzymatic reaction was induced using a Clarity Western ECL Substrate (1705060, BIO-RAD Laboratories, Hercules, CA) and detected by a ChemiDoc Touch (BIO-RAD Laboratories).

The culture supernatants from PStRAP mRNA-transfected hepatocytes were analyzed using a Jess Simple Western system (ProteinSimple, San Jose, CA) according to the instructions provided by the manufacturer. The DYKDDDDK Tag Rabbit mAb (clone D6W5B, 1/50 dilution; #14793 Cell Signaling Technology, Danvers, MA) was used for detection.

### Lipid-protein interaction assay

To assess the direct lipid-binding ability and specificity of PStRAP, a lipid-protein interaction assay was performed using Membrane Lipid Strips (P-6002, Echelon Biosciences, Salt Lake City, UT), according to the instructions provided by the manufacturer. Briefly, membranes were blocked in 3% bovine serum albumin (BSA) in PBS and then incubated for 1 h with the culture supernatants from the PStRAP mRNA-transfected hepatocytes. After washing the membranes, they were incubated for 1 h with 1/1,000 diluted DYKDDDDK Tag Rabbit mAb (clone D6W5B; #14793, Cell Signaling Technology) followed by incubation with 1/10,000 diluted Peroxidase-conjugated AffiniPureR Alpaca Anti-Rabbit IgG (H + L). The enzymatic reaction was induced using a Clarity Western ECL Substrate and detected by a ChemiDoc Touch.

### Detecting PS with flow cytometry

MCA205 cells were induced to undergo apoptosis via treatment with 500 μM of L-OHP (O0372, Tokyo Chemical Industry: TCI, Tokyo, Japan) for 24 h. To confirm the state of MMC-pulsed cells, MCA205 was precultured with 500 μM of MMC (M2320, TCI) or vehicle (dimethyl sulfoxide) for 3 h. The cells were washed three times and then continued to be cultured, and they were harvested 3, 24, and 48 h after MMC treatment. The thymocytes from the C57BL/6NJ mice were cultivated with 10 μM of dexamethasone (Dex; D1961, TCI) in complete DMEM for 4 h.^5^ The PS on the cell surface was detected using Brilliant Violet 421 (BV421) annexin V (640923, BioLegend, San Diego, CA), and dead cells were costained with 7-AAD (420403, BioLegend) or Helix NP NIR (425301, BioLegend). As a method to detect PS instead of annexin V, the apoptosis-induced cells were incubated with the culture supernatants from PStRAP- or mock-mRNA-transfected hepatocytes followed by staining with a BV421 anti-DYKDDDDK Tag antibody (clone L5; 637322, BioLegend). For the competitive assay, either PStRAP- or mock-supernatant was added during annexin V staining. The stained cells were detected using LSRFortessa X-20 (Becton, Dickinson and Company: BD, Franklin Lakes, NJ), and the results were analyzed using FlowJo v10.8.1 (BD).

### NF-κB reporter assay

pNiFty2-N-Lucia-Blasti vector-transfected RAW264.7 cell sublines were seeded on a 96-well flat-bottom plate (353072, Corning Inc.) at a density of 1 × 10^5^ cells per well. Cells were treated with the culture supernatants from PStRAP- or mock-mRNA-transfected hepatocytes, 40 μg/mL of recombinant mouse RAP protein (4480-LR-050, R&D Systems, Minneapolis, MN), or with no treatment in complete DMEM for 1 h. After treatment, 20 μL of the supernatant was transferred to a 96-well white plate (3912, Corning Inc.), and 50 μL of QUANTI-Luc 4 Lucia/Gaussia Flash solution (rep-qlc4lg, InvivoGen) was added. The luciferase activity was measured using Infinite M Plex (Tecan, Männedorf, Switzerland). NF-κB activity was indicated as a fold change with respect to the activity in the untreated group as a reference.

### Phagocytosis assay

The apoptosis-induced thymocytes from the C57BL/6NJ mice were cultivated with 10 μM of Dex in complete DMEM for 4 h and then labeled with 5 μM CypHer5E (PA15401, cytiva) in HBSS (Hanks' Balanced Salt Solution; 14175, Thermo Fisher Scientific) for 30 min at 37°C. The CypHer5E is a pH-sensitive dye that exhibits maximally fluorescent in the acidic conditions of late phagosomes and demonstrates resistance to fluorescence quenching in this environment.^63^ A total of 50,000 RAW264.7 cells, which were primed by 200 U/mL of IFNγ (575306, BioLegend) and labeled with CFSE (Carboxyfluorescein Succinimidyl Ester; C34554, Thermo Fisher Scientific) one day before the assay, was cocultured with 100,000 apoptosis-induced cells for 2 h in conditioning medium containing PStRAP- or mock-supernatant. For the competitive assay, either 20 μg/mL of purified annexin V protein (640901, BioLegend) or 40 μg/mL of mouse RAP protein was added to the medium during co-culturing. After the residual apoptotic cells were removed, the phagocytes were analyzed using flow cytometry. The percentage of CypHer5E-positive RAW264.7 cells was considered the rate of phagocytosis.

### *In vivo* tumor challenges and treatments

Seven- to nine-week-old female mice were subcutaneously transplanted into the right flank with the desired number of tumor cells (MCA205 and its sublines: 5 × 10^5^ cells; MC38: 3 × 10^5^ cells) suspended in 100 μL of HBSS on day 0. Tumor volumes were calculated as (long diameter × square short diameter) × π/6, which were measured using calipers every other day until reaching 10% of the body weight. All animal studies are indicated in figures as individual values (dotted lines) and means (bold lines). To assess the therapeutic effect of PStRAP, MCA205-bearing mice on day 7 were treated with LNP/mRNA (1 mg/kg) or empty LNP as a control intravenously 6 h prior to intraperitoneally administering 10 mg/kg of L-OHP on the same day.

When the studies aimed at evaluating the induction of adaptive immune responses, tumor cells were precultured with 500 μM of MMC in complete DMEM for 3 h, and then the left flank of the mice was subcutaneously inoculated with the dying tumor cells (5 × 10^5^ cells) suspended in 100 μL of PBS. To evaluate whether cell death induced by the endogenous CTL response induces the adaptive immune response, eight-week-old male OT-I mice were subcutaneously transplanted into the left flank with the viable OVA-expressing tumor cells (5 × 10^5^ cells) suspended in 100 μL of HBSS. Viable parental cells were subcutaneously transplanted into the right flank seven days later.

### *In vitro* restimulation with neoepitopes

The splenocytes were harvested from the tumor-bearing mice 26 days after MC38 transplantation (Figure 2C) and seeded in 48-well plate (3338, Corning Inc.) at 1 million cells per well in complete RPMI. The following three known neoepitope peptides of MC38 were synthesized by GeneScript: H-2D^b^-restricted ADPGK (ADP-dependent glucokinase); ASMTN***M***ELM, H-2D^b^-restricted REPS1 (RalBP1-associated Eps domain-containing protein 1); AQL***A***NDVVL, H-2K^b^-restricted RPL18 (Ribosomal Protein L18); KILTFD***R***L.^20^^,^^21^ The splenocytes cultured with 10 μM of individual peptide or no peptide for three days, the culture supernatants were then collected, and the amounts of IFNγ and TNFα were measured using ELISA.

### *Ex vivo* cross-presentation

MMC-pulsed parental MCA205, MCA205-OVA, or MCA205-OVA-PStRAP cells (2 × 10^6^ cells) were subcutaneously inoculated into the right flank. The tumor-draining subiliac lymph nodes were harvested one day later, and the APCs were prepared by removing T-, B-, and NK cells.^7^ The effector CTLs were prepared from the spleens of the OT-I mice using an EasySep Mouse CD8a Positive Selection Kit II (#18953, STEMCELL Technologies, Vancouver, Canada). A total of 1 million effector cells were cocultured with 250,000 APCs in complete RPMI on a 24-well plate (3337, Corning Inc.) for three days. The amounts of IFNγ in the culture supernatants were measured using ELISA.

### *In vivo* T-cell proliferation assay

CD8^+^ T-cells were enriched by EasySep Mouse CD8a Positive Selection Kit II (18953, STEMCELL Technologies, Vancouver, Canada) from splenocytes of OT-I mice and were labeled with CFSE. CFSE-labeled OT-I cells were adoptive transferred into wild-type C57BL/6NJ mice via tail vein injection (1.2 × 10^6^ cells per mouse). One day later, recipient mice were subcutaneously inoculated with MMC-pulsed MCA205-OVA, MCA205-OVA-PStRAP, or MCA205-OVA-PStRAP-RAP cells (5 × 10^5^ cells) into the right flank. The tumor-draining subiliac lymph node was harvested three days later, and the lymphocyte were stained with BV421 anti-mouse CD8a Antibody (clone 53-6.7; 100753, BioLegend) and PE anti-mouse TCR Vβ5.1, 5.2 Antibody (clone MR9-4; 139504, BioLegend) for distinguishing the donor cells. The stained cells were analyzed using flow cytometry.

### Sandwich ELISA

The amounts of mouse IFNγ and TNFα in the culture supernatants were detected using Mouse IFN-gamma DuoSet ELISA and Mouse TNF-alpha DuoSet ELISA (DY485 and DY410, respectively, R&D systems) according to the instructions provided by the manufacturer.

### Cytokine array

MCA205-PstRAP or MCA205-empty cells (3 × 10^6^ cells) were cultured in a φ60 dish (VTC-D60, AS ONE, Osaka, Japan). In the event of cell death induction, these cells were precultured with 500 μM of MMC. 3 h later, the cells were washed three times, and the medium was exchanged. After cultivation for 24 h, culture supernatants were collected. To detect 111 mouse cytokines, chemokines, and growth factors, 1 mL of culture supernatants was loaded onto membrane of Proteome Profiler Mouse XL Cytokine Array (ARY208, R&D Systems) and processed according to the manufacturer’s protocol. Densitometric analysis was performed using the Protein Array Analyzer plugin (https://rsb.info.nih.gov/ij/macros/toolsets/Protein Array Analyzer.txt) on ImageJ.^64^

### Quantification of luciferase bioluminescence intensity

To validate the delivery of the LNPs, Fluc mRNA-encapsulated LNPs were used. Seven-week-old mice were intravenously injected with these LNPs, and the desired organs were harvested 6 and 24 h later, accompanying an intravenously injected D-Luciferin (potassium salt) (14681, Cayman Chemical, Ann Arbor, MI) immediately before. The luminescence from the harvested organs *ex vivo* was measured using an IVIS Imaging System (PerkinElmer, Waltham, MA).

### Detecting protein in serum

Blood was collected from the superficial temporal vein of the mice at the desired time points using Goldenrod Animal Lancets 4 mm (18310300, MEDIpoint, Long Island, NY). Blood samples were allowed to stand for 1 h followed by centrifugation (2,000 × *g*, 10 min), and then the resulting serum was collected. An AlphaScreen FLAG Detection Kit (6760613, Revvity, Waltham, MA) was used to detect DYKDDDK-tagged protein in the serum. The ALT and AST levels were measured using a Transaminase CII-test (431–30901, FUJIFILM Wako). Each kit was performed according to the instructions provided by the manufacturer.

### Statistical analysis

All statistical analyses were performed using the GraphPad Prism 8 (GraphPad Software, San Diego, CA). For comparison of the means between groups, an unpaired Welch’s *t* test was applied. Comparisons of the means among two or more groups were performed using one- or two-way analysis of variance (ANOVA) followed by post hoc analysis with Tukey’s test in some cases. Data are presented as the mean ± standard deviation (SD). The *p* values <.05 were considered statistically significant.

## Data and code availability

All data supporting the findings of the study are available in the article and its supplemental information. All raw data are available from Y.M. upon reasonable request.

## Acknowledgments

We would like to thank Mr. Takeshi Ueda (Nitto Denko Corporation) for supporting us throughout this study. We thank Drs. Takashi Akazawa and Tomoya Ekawa (10.13039/100024217Osaka International Cancer Institute) for fruitful discussions as well as Mr. Hiroya Komaki and Mses. Yachiyo Kumamoto, Arisa Tsuda, Kazuko Shizuma, and Mitsuki Yamaguchi (10.13039/100024217Osaka International Cancer Institute) for supporting the experiments. We thank Dr. William R. Heath for providing the OT-I mice and Dr. Steven. A. Rosenberg for providing the murine cell lines. We would like to thank Editage (www.editage.jp) for the English language editing. This study was funded by the 10.13039/501100001691JSPS KAKENHI under grant number JP22K07268 (Y.M.).

## Author contributions

Y.M.: conceptualization, data curation, formal analysis, funding acquisition, investigation, project administration, resources, visualization, writing – original draft, writing – review and editing. H.N.: data curation, investigation, resources, validation, visualization, writing – original draft, writing – review and editing. K.H.: resources, writing – review and editing. C.O.: resources, writing – review and editing. S.A.: writing – review and editing. K.M.: writing – review and editing. H.T.: conceptualization, funding acquisition, supervision, writing – original draft, writing – review and editing.

## Declaration of interests

Y.M. and H.T. are the inventors of patents application (PCT/JP2024/041545 and JP2023-199462) associated with this publication. H.N., K.H., C.O., S.A., and K.M. were employees of the Nitto Denko Corporation (Osaka, Japan) at the time of this study.

## Supplemental information

Document S1. Figures S1–S10

Document S2. Article plus supplemental information
