# Supplementary material for: Restricting efferocytosis pathway to CD91 via phosphatidylserine-targeting chimeric protein augments antitumor immune responses
Source: Mol Ther. 2026 May 26;34(8):4729–42. doi: 10.1016/j.ymthe.2026.05.013 (PMC13464154; doi:10.1016/j.ymthe.2026.05.013)
Supplement: Supplementary file 1 [file mmc1.pdf]

YMTHE, Volume *34*

Supplemental Information

Restricting efferocytosis pathway to CD91 via phosphatidylserine-targeting chimeric protein augments antitumor immune responses

Yu Mizote, Hiroki Nishita, Kazuki Hashiba, Chisa Okuma, Sachiko Akane, Kenjiro Minomi, and Hideaki Tahara

A

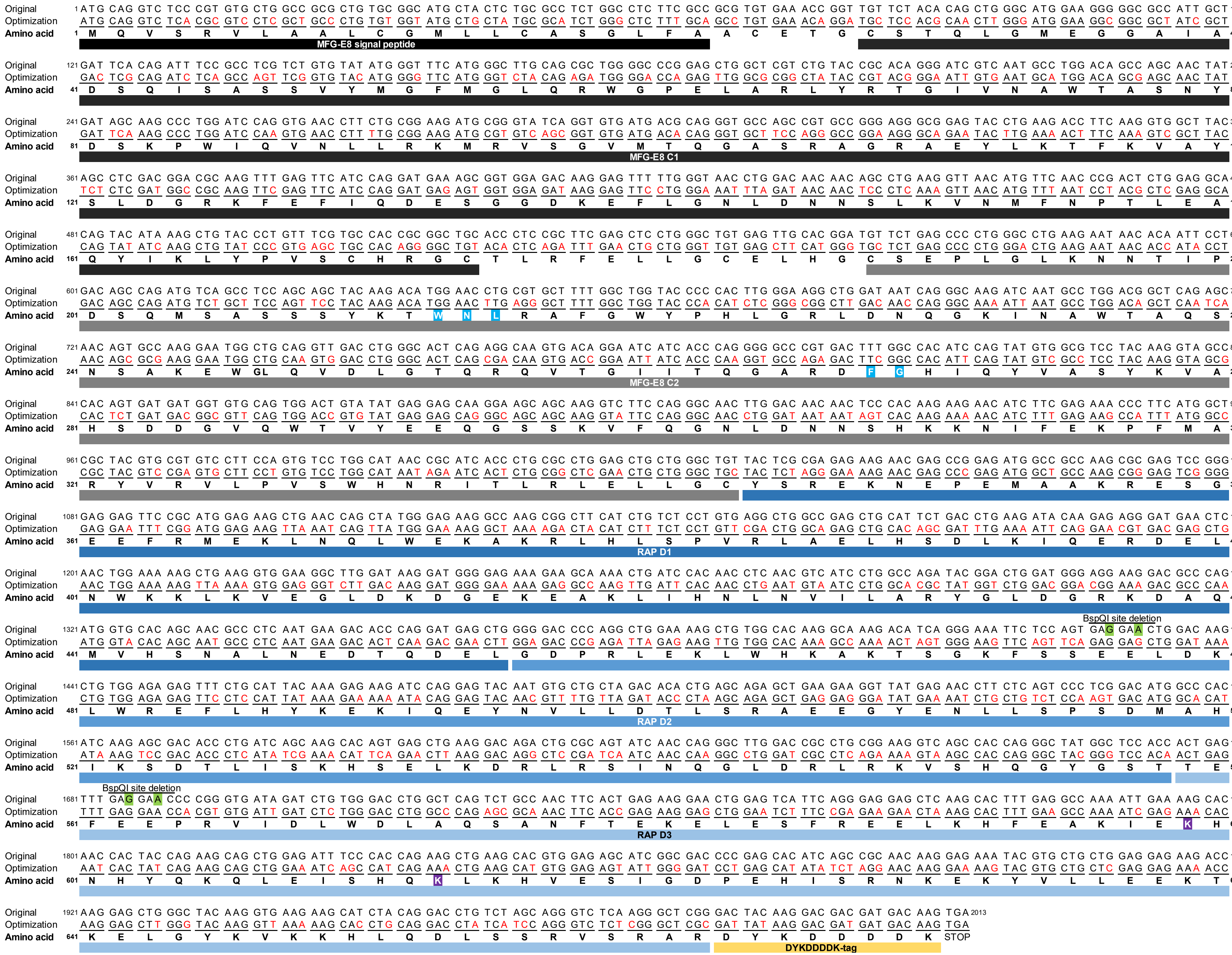
Original
Optimization
Amino acid
MFG-E8 signal peptide
MFG-E8 C1
MFG-E8 C2
RAP D1
RAP D2
RAP D3
BspQI site deletion
BspQI site deletion
DYKDDDDK-tag
STOP

B

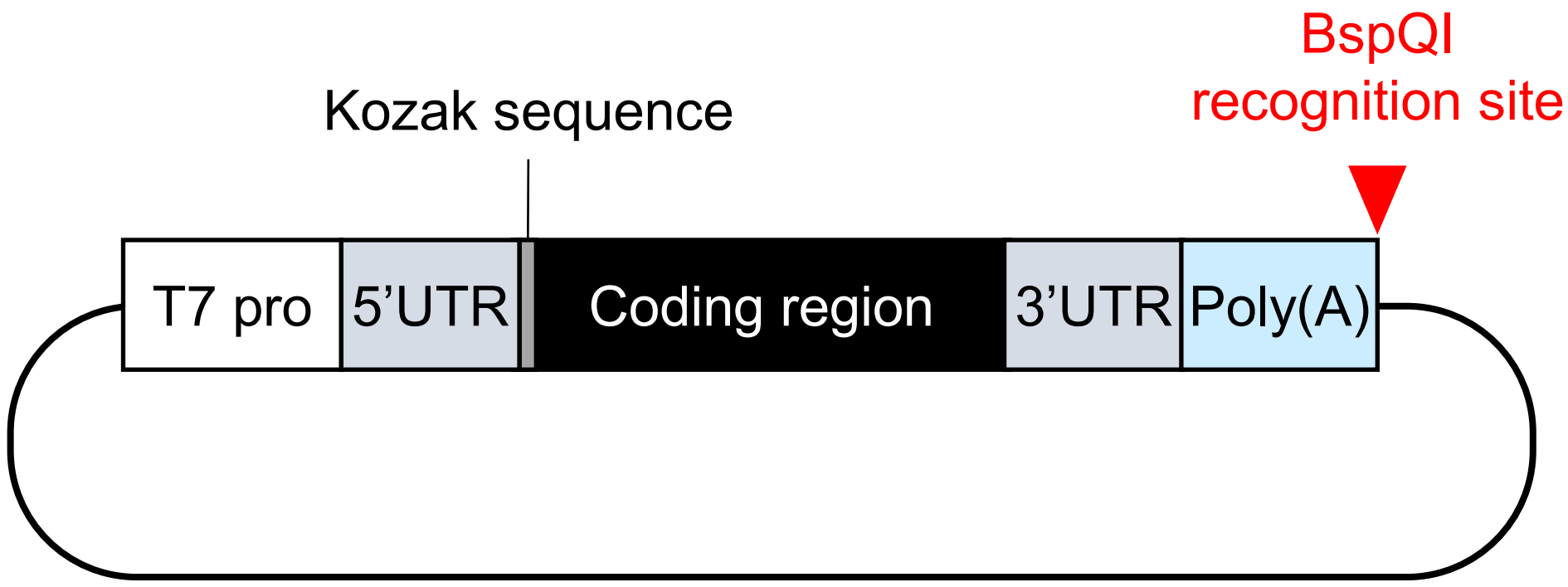

Figure S1. PStRAP sequence and DNA plasmid structure for synthesizing mRNA.

(A) The PStRAP original DNA, codon-optimized DNA, and amino acid sequences are depicted. The BspQI recognition site (GAAGAGC) was deleted from the spontaneous sequence to linearize the plasmid (green). The DNA replaced for optimizing the codon is shown red letters. Each amino acid (blue) was replaced with alanine in the PS binding activity deletion mutant (PStRAPΔPS). Each amino acid (purple) was replaced with alanine in the CD91 binding activity deletion mutant (PStRAPΔCD91). (B) The plasmid construct for synthesizing mRNA contains a T7 promoter, a 5'-untranslated region (UTR), a Kozak sequence, a coding region, a 3'-UTR, a 120 nt poly(A) tail, and a BspQI recognition site for linearization, in this order.

A

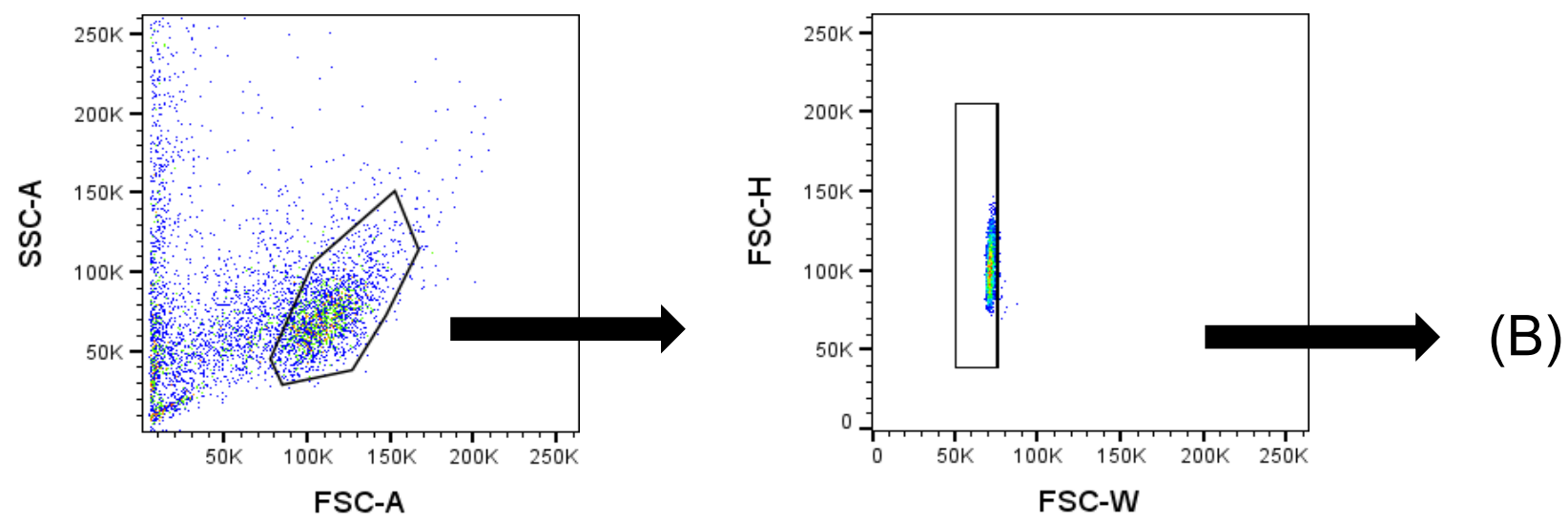

B

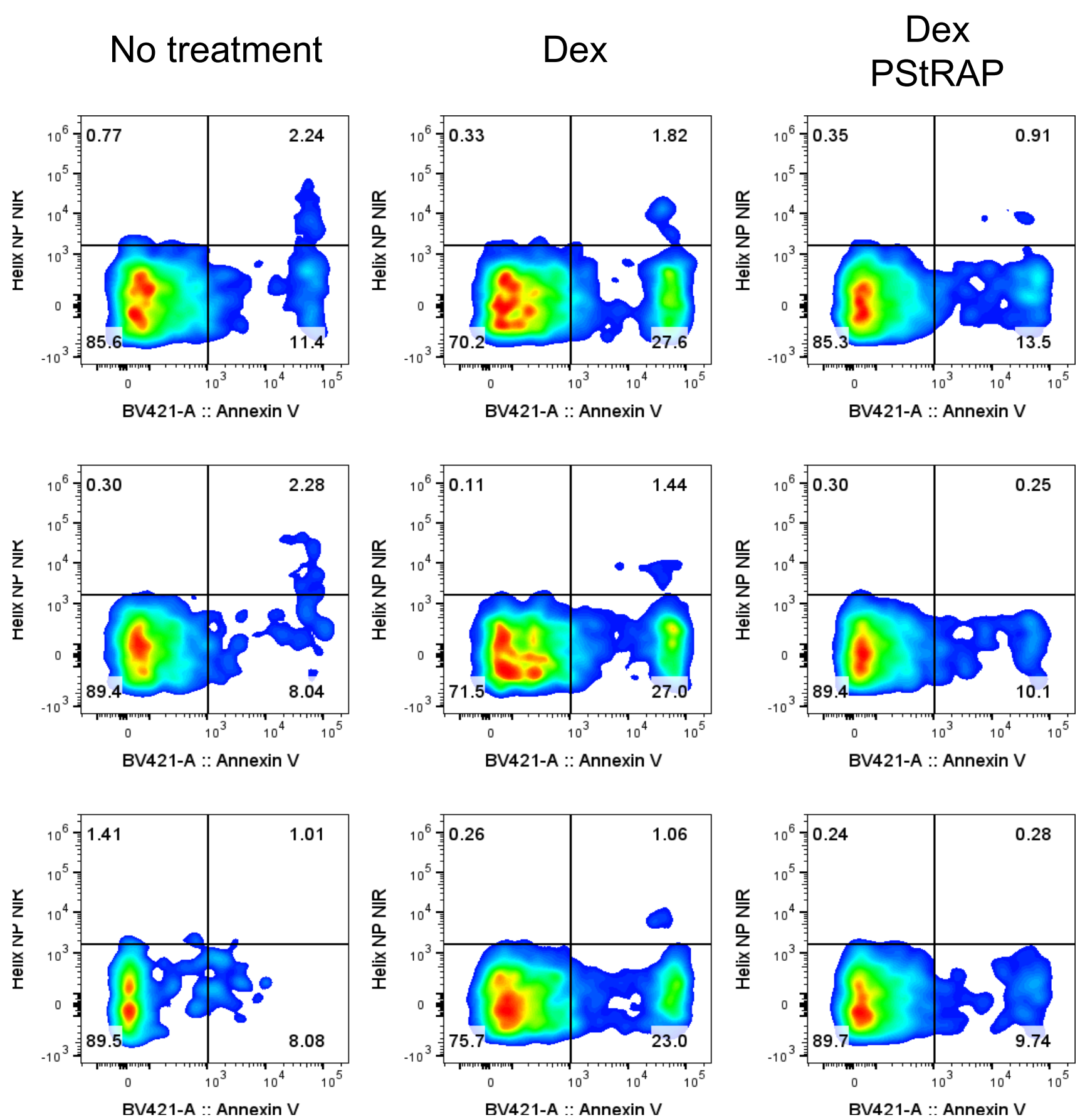

	No treatment	Dex	Dex PStRAP
Annexin V gMFI	197	650	225
	149	590	173
	154	454	73.1

Figure S2. Gating strategy of Annexin V staining and individual plots.
(A) The Annexin V^{+} cells were defined by gating FSC/SSC and then single cell-populations, in that order. (B) Individual plots are depicted (*n* = 3/group). Individual geometric mean fluorescent intensity (gMFI) of annexin V is shown in a table. The relative gMFI is depicted in Figure 1E

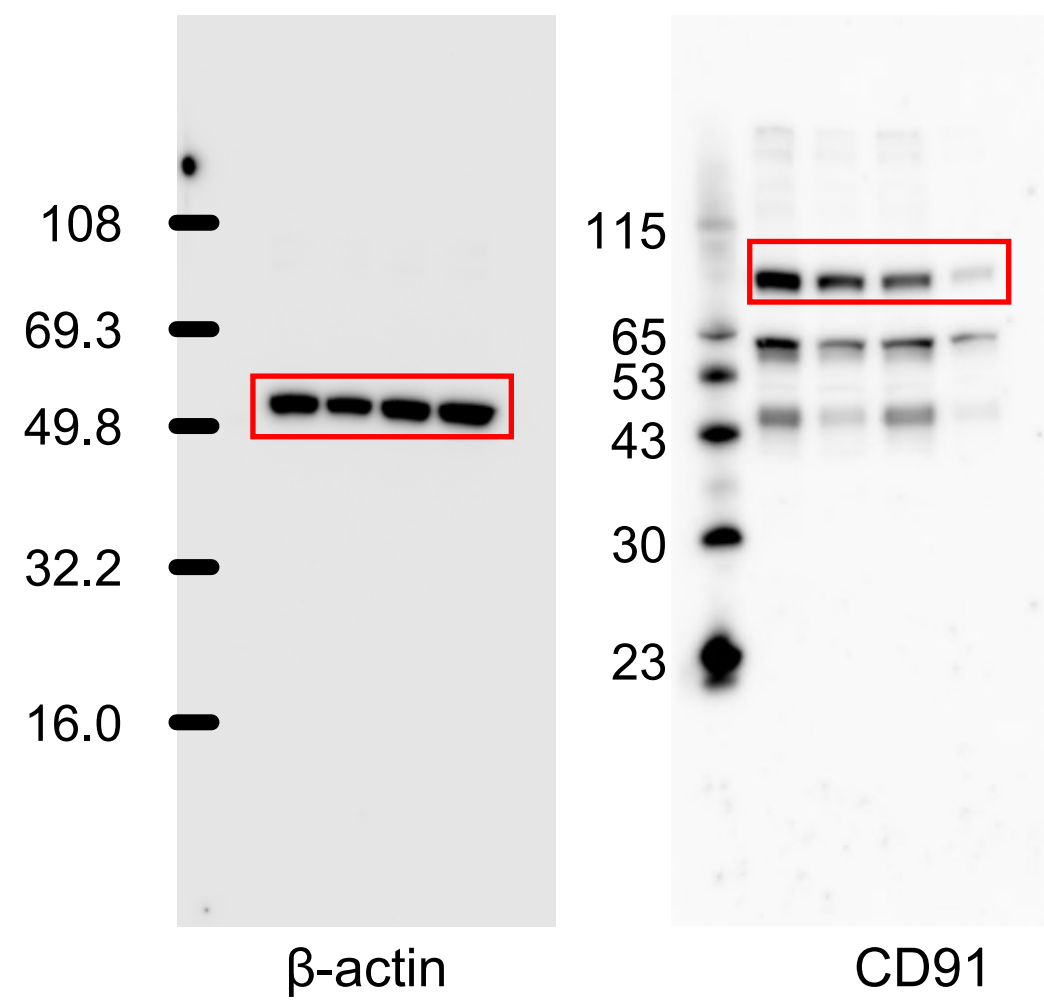

Figure S3. Expression levels of CD91 in RAW264.7 cell sublines. Original blot images depicting cropped regions for Figure 1F.

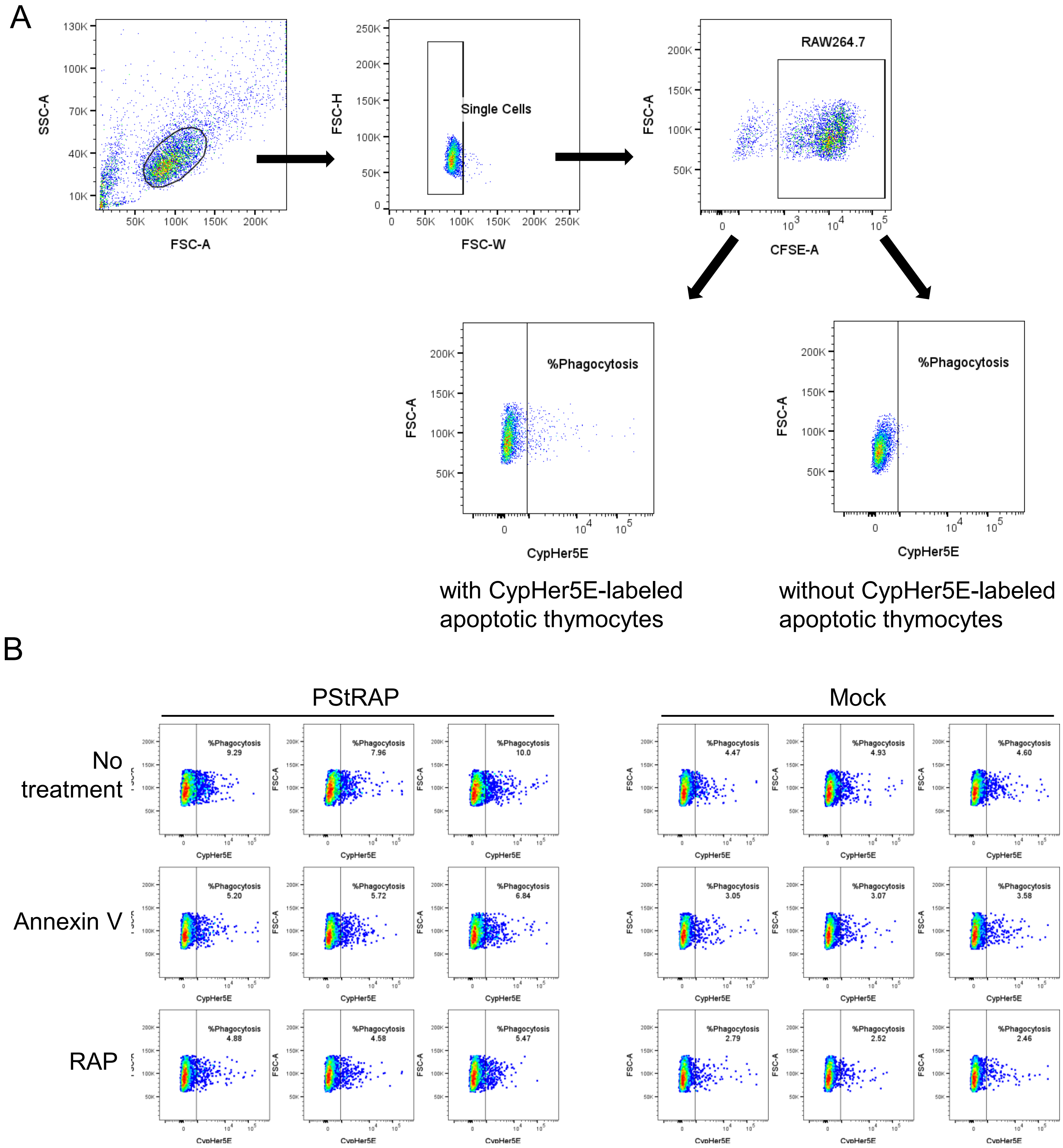

Figure S4. Gating strategy and phagocytosis assay.
(A) The CypHer5E⁺ RAW264.7 cells were defined by gating single cell-populations and then CFSE⁺, in that order. (B) The CypHer5E⁺ cell rate is depicted as a percentage of phagocytosis in Figure 1H (n = 3/group).

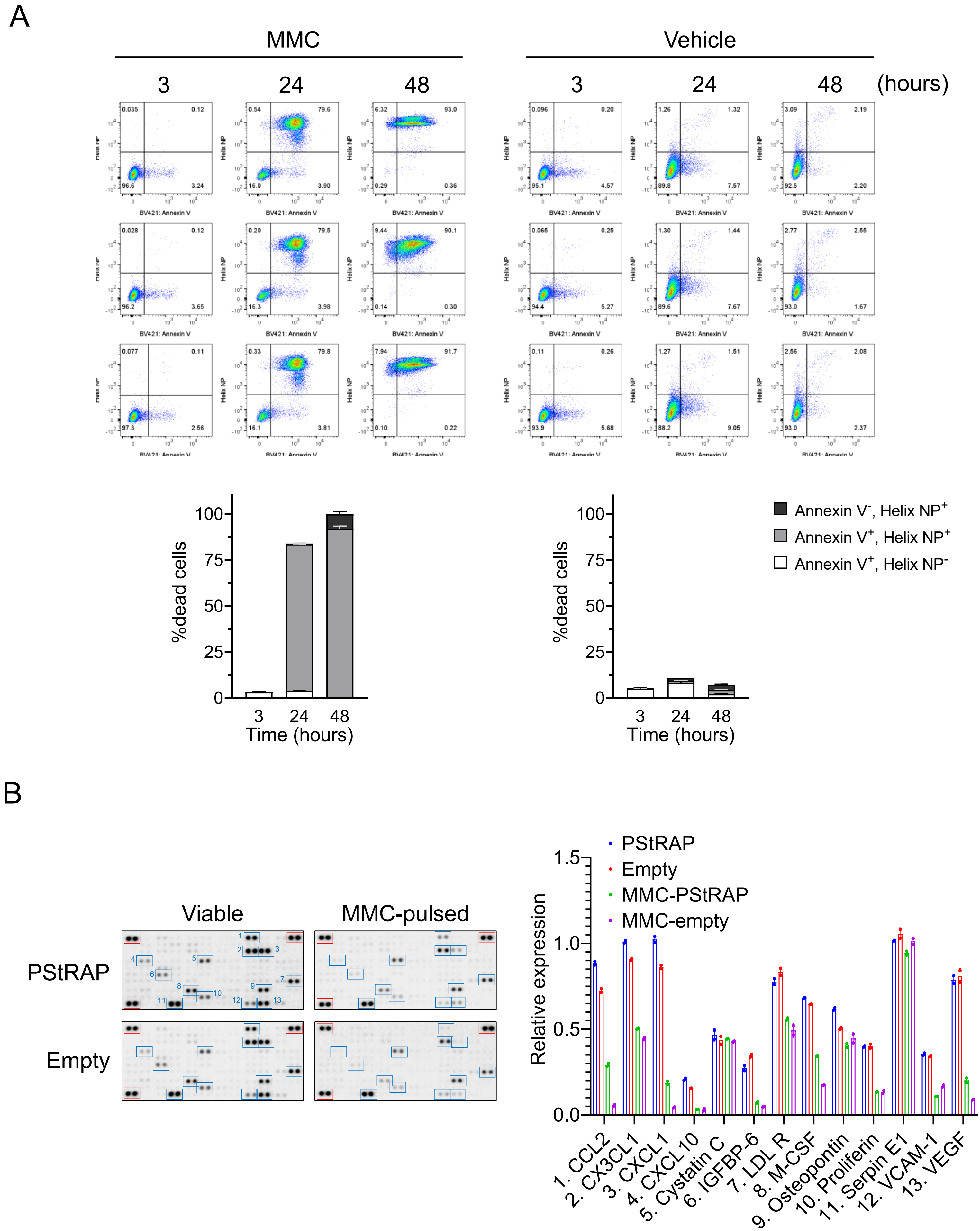
A
MMC
Vehicle
3
24
48
(hours)
BV421: Annexin V
Helix NP
%dead cells
Time (hours)
Annexin V-, Helix NP+
Annexin V+, Helix NP+
Annexin V+, Helix NP-
B
Viable
MMC-pulsed
PStRAP
Empty
PStRAP
Empty
MMC-PStRAP
MMC-empty
Relative expression
1. CCL2
2. CX3CL1
3. CXCL1
4. CXCL10
5. Cystatin C
6. IGFBP-6
7. LDL R
8. M-CSF
9. Osteopontin
10. Proliferin
11. Serpin E1
12. VCAM-1
13. VEGF

Figure S5. The characteristic of MMC-pulsed tumor cells.
(A) MMC (500 μM) or vehicle-pulsed MCA205 cells were co-stained with BV421-conjugated annexin V and Helix NP NIR at the desired time point (*n* = 3/group). Error bars show mean (SD). (B) The secreted factors in culture supernatant were detected using a membrane array. The culture supernatant was collected from viable or MMC-pulsed MCA205 transfectants (PStRAP or Empty), after culturing for 24 hours. The red boxes indicate the reference spots, while the blue box with a number indicates spots detected in the supernatant of viable cells (left). The density of the spots relative to the average value of the reference spots indicates the relative expression level of the secreted factor (right).

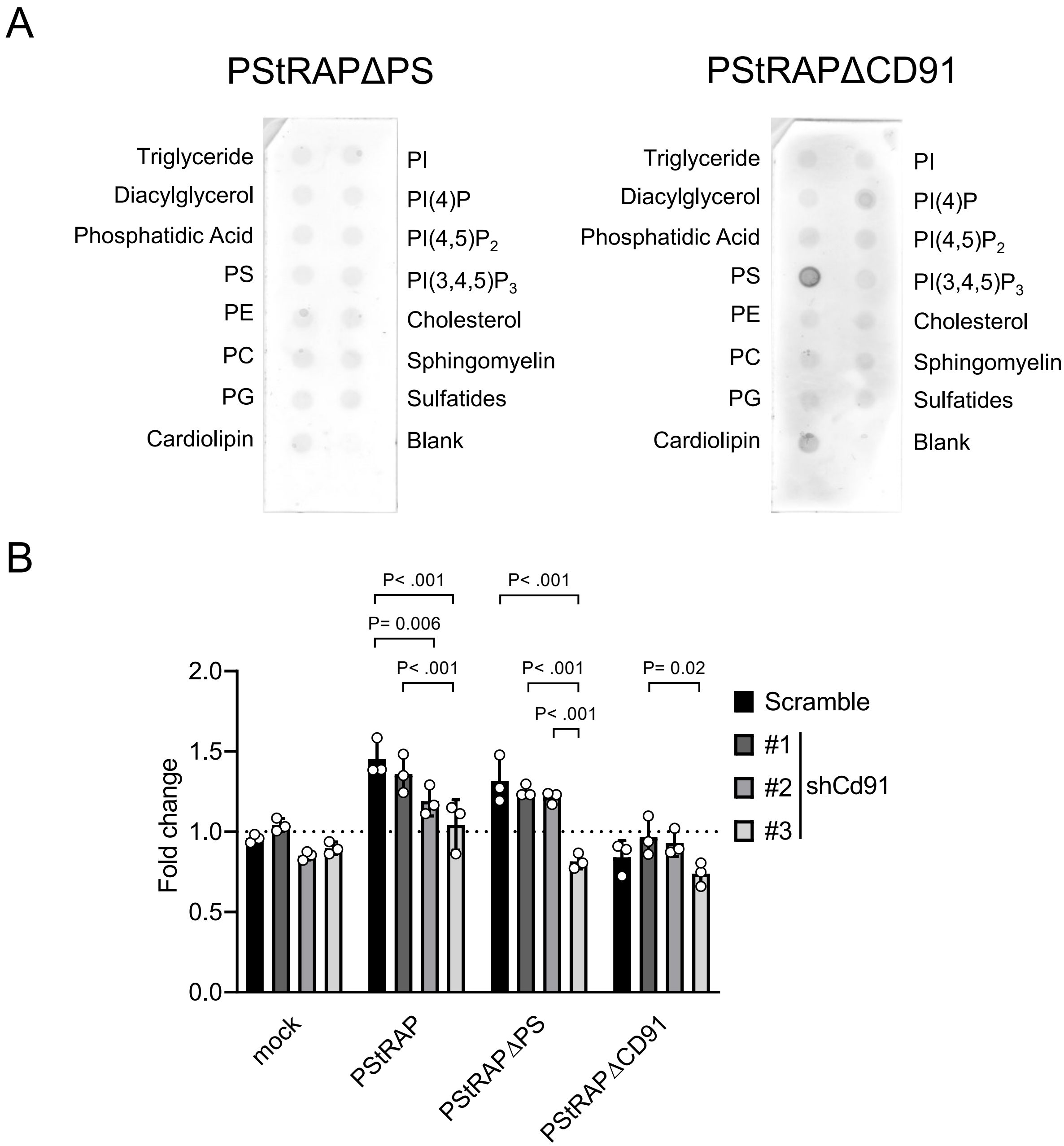

Figure S6. Loss-of-function mutants of PStRAP.

(A) A membrane lipid array was performed with the culture supernatants from MCA205 transfectants after two days of culturing. (B) The luciferase secreted upon NF-κB activation was measured in the culture supernatants from the RAW264.7 cell sublines (Figure 1E) one hour after stimulation with the culture supernatants of the transfectants (n = 3/group). The fold change was calculated using the luciferase level in the culture supernatants without stimulation as a reference. Error bars show mean (SD). Significance differences were determined using two-way ANOVA followed by post hoc Tukey's test.

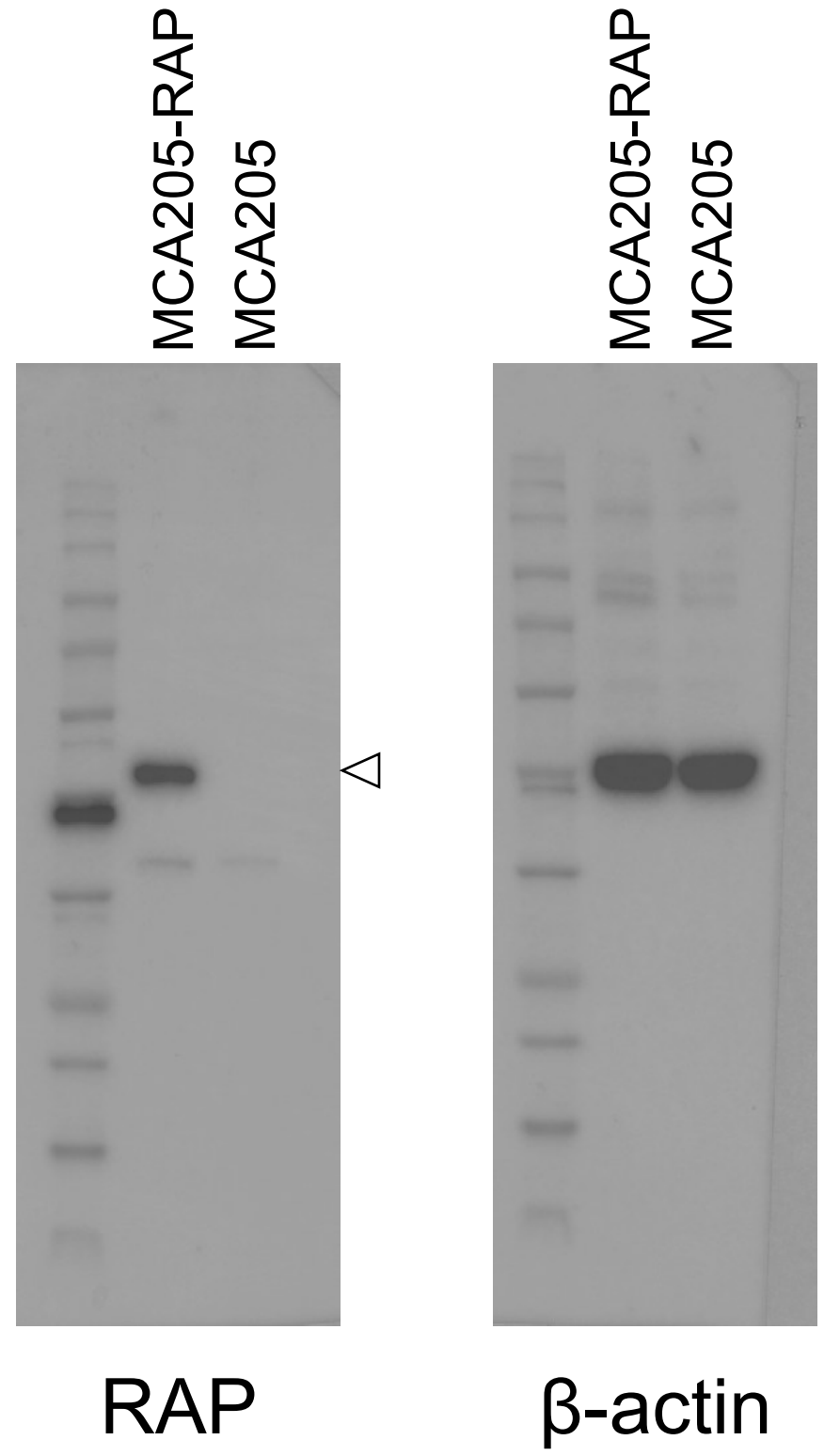

Figure S7. Expression of RAP in transfectant.
RAP expression in MCA205 transfectant used in Figures 3B and 3C was detected by immunoblotting. The RAP protein, which was tagged with PA at the C-terminus for differentiate its endogenous expression, was detected using an anti-PA tag antibody.

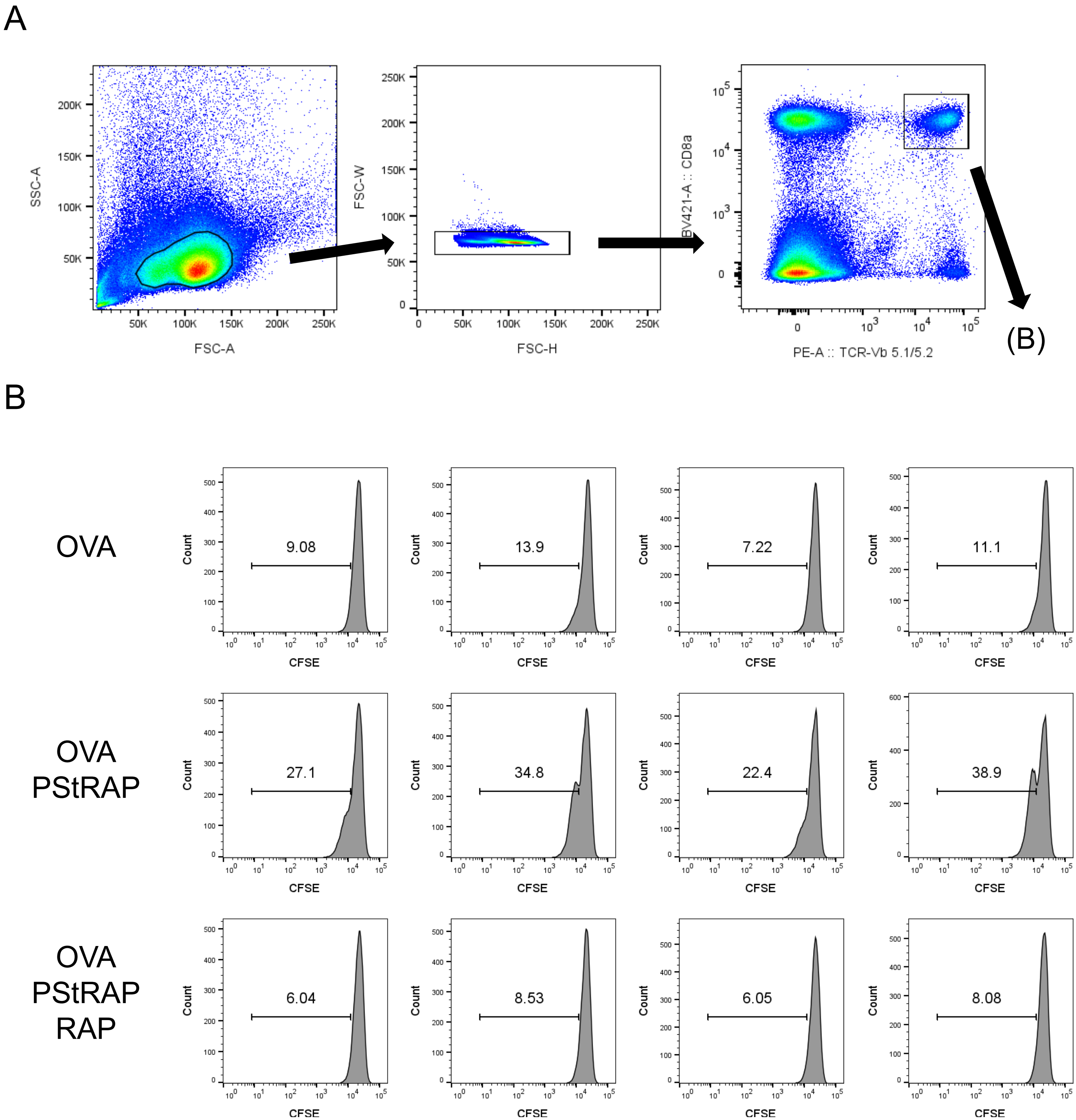

Figure S8. ***In vivo*** **$CD8^+$ T-cell proliferation assay.**

(A) Gating strategy for the detection of adoptively transferred $CD8^+$ T-cells from the OT-I mouse. The both TCR-Vβ5.1/5.2- and CD8α-positive cells were defined by gating FSC/SSC and then single cell-populations, in that order. (B) Histograms from the flow cytometry analysis of gated TCR-$V\beta5^+CD8^+$ OT-I cells from representative mice of each group (n = 4). The dividing OT-I cell rate is depicted as a percentage in Figure 3C.

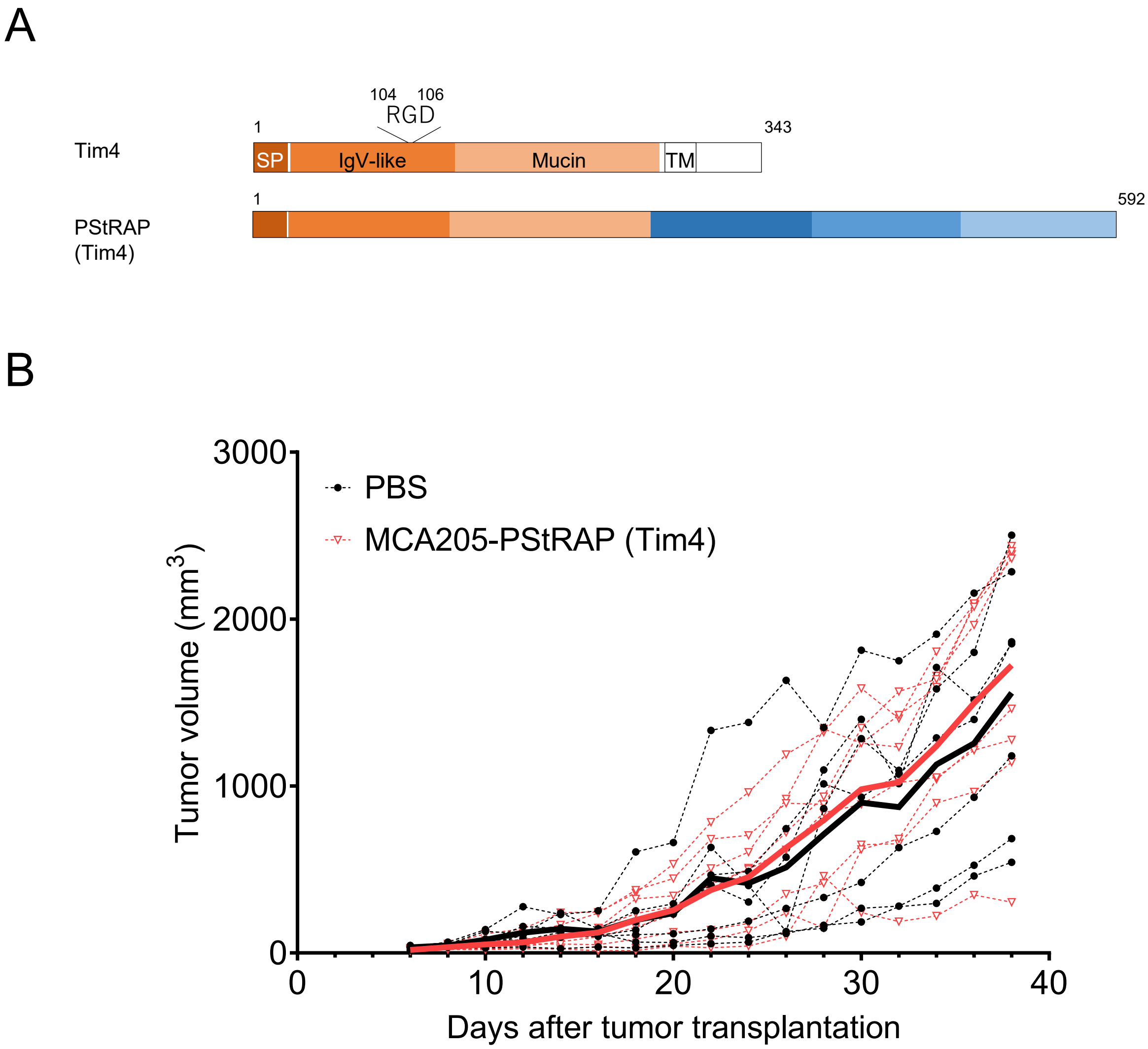

Figure S9. Structure and function of alternative PStRAP (Tim4).

(A) Structure of Tim4 and PStRAP (Tim4). SP; signal peptide, TM; transmembrane region. (B) MMC-pulsed MCA205-PStRAP (Tim4) (5 × 10^5 cells) cells were subcutaneously inoculated into the left frank of the mice. Viable MCA205 (5 × 10^5 cells) cells subcutaneously transplanted into the right frank seven days later, and then tumor volumes were measured every other day (n = 8/group). PBS group was same data in Figure 3E. Significance differences were determined using two-way ANOVA.

A

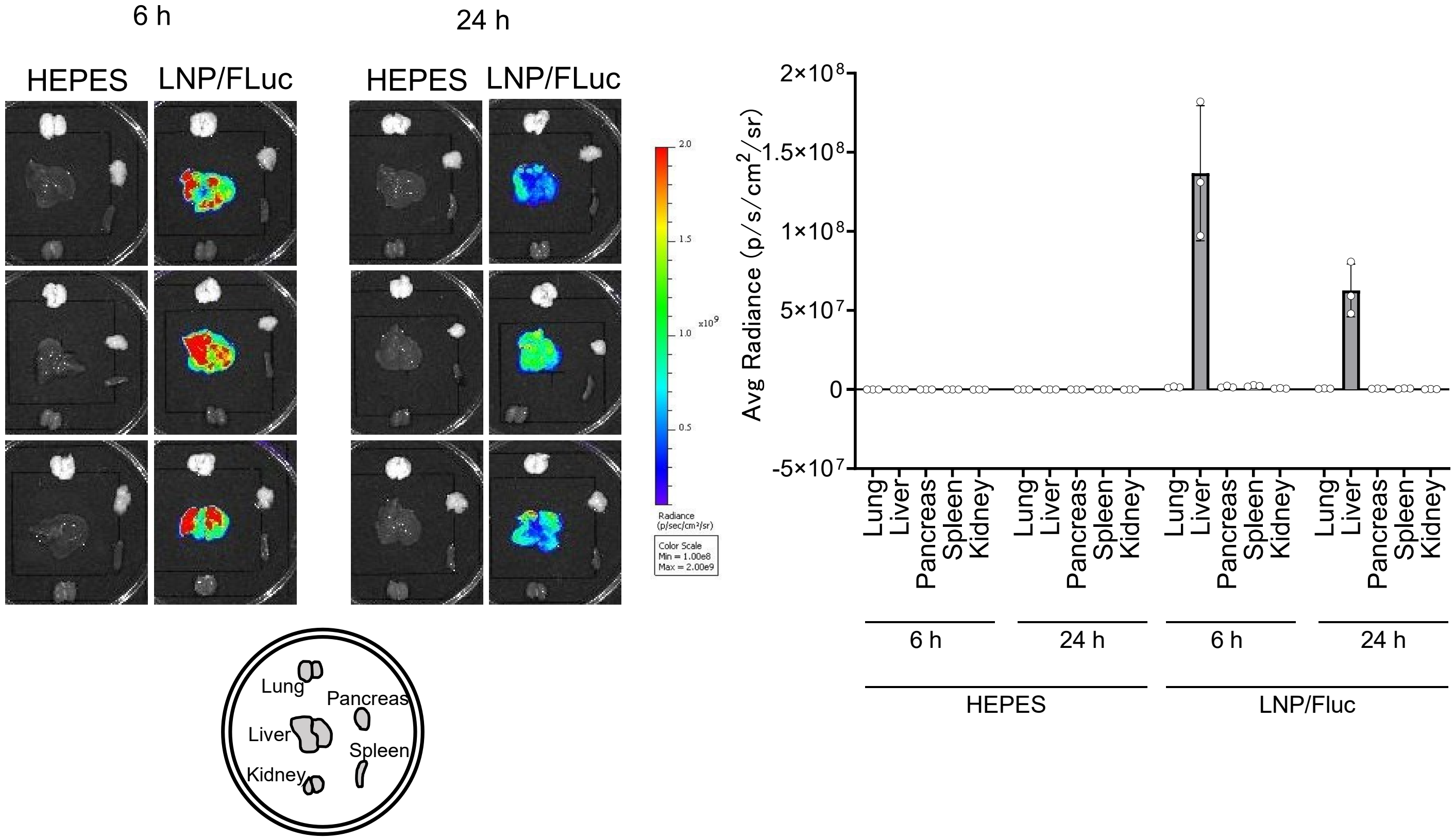
6 h
24 h
HEPES
LNP/FLuc
HEPES
LNP/FLuc
Radiance (p/sec/cm²/sr)
Color Scale Min = 1.00e8 Max = 2.00e9
Lung
Pancreas
Liver
Spleen
Kidney
Avg Radiance (p/s/cm²/sr)
2×10⁸
1.5×10⁸
1×10⁸
5×10⁷
0
-5×10⁷
Lung
Liver
Pancreas
Spleen
Kidney
6 h
24 h
6 h
24 h
HEPES
LNP/Fluc

B

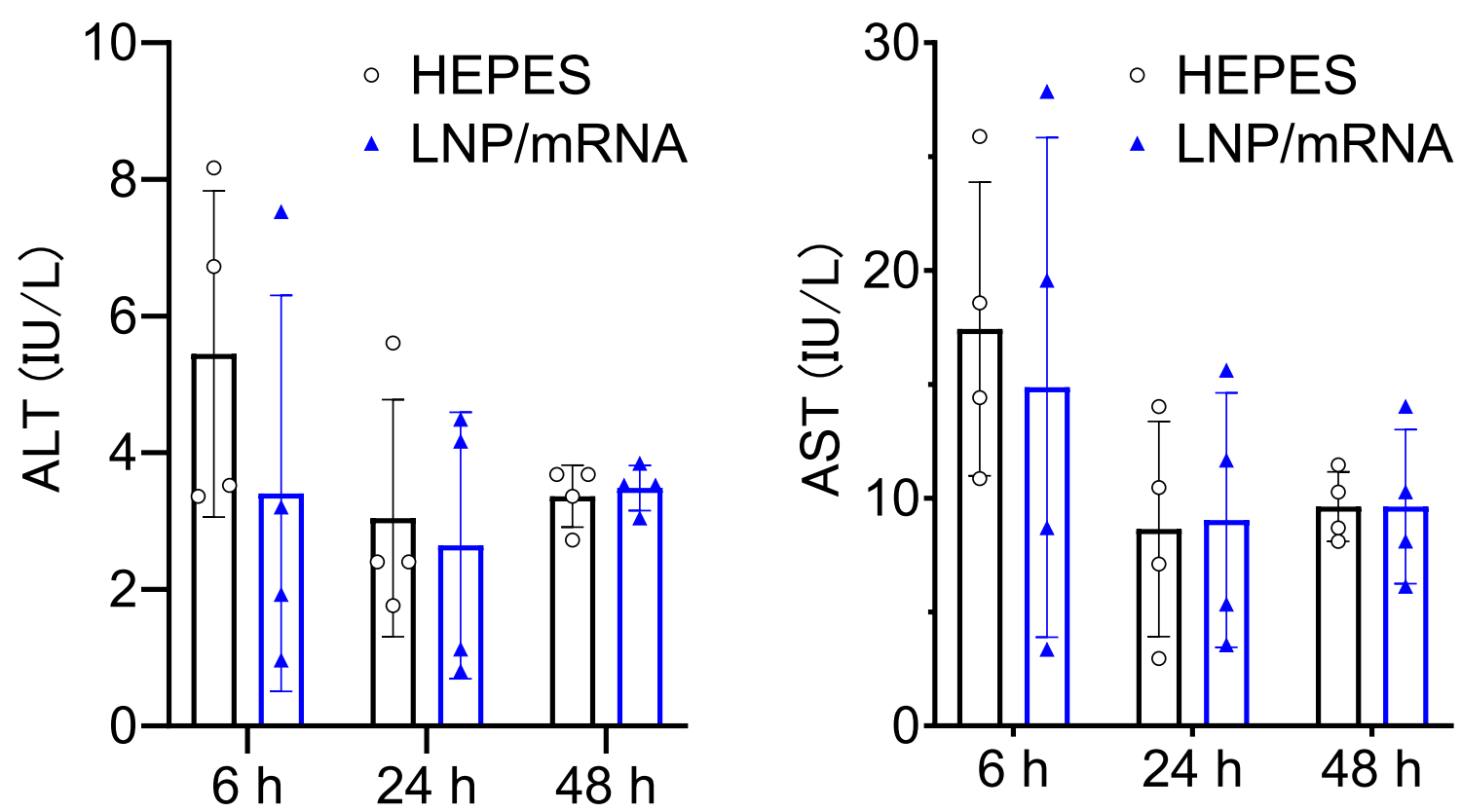
HEPES
LNP/mRNA
ALT (IU/L)
AST (IU/L)
6 h
24 h
48 h

C

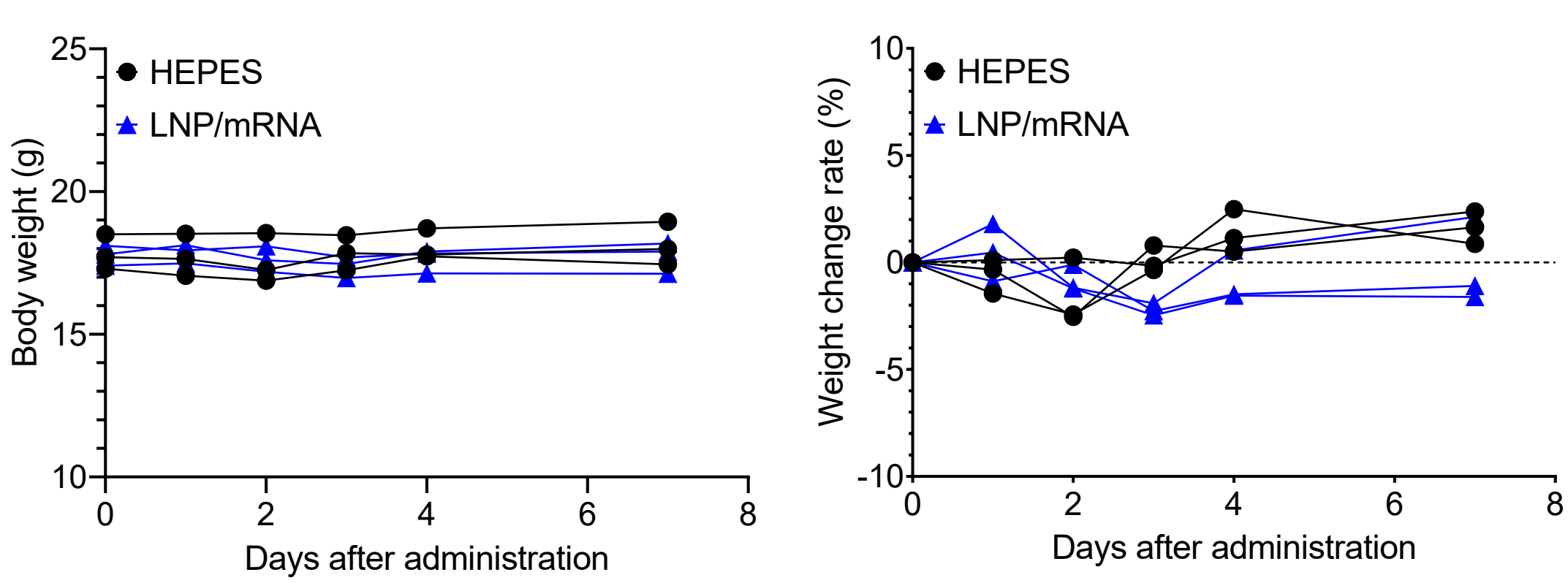
HEPES
LNP/mRNA
Body weight (g)
Weight change rate (%)
Days after administration

Figure S10. LNP/mRNA delivery and toxicity.
(A) The luminescence of the desired mouse organs (*n* = 3/group) at 6 and 24 hours (h) after intravenous LNP/Fluc or HEPES injection are shown. Error bars show mean (SD). (B) The amounts of ALT and AST in serum from mice (*n* = 4/group) were measured at the indicated time points after intravenous LNP/mRNA or HEPES injection. Error bars show mean (SD). Significance differences were determined using an unpaired Welch's *t*-test. (C) The body weights of the mice (*n* = 3/group) were measured for seven days after intravenous LNP/mRNA or HEPES injection, and fluctuations (left) and change rates (right) are indicated. Significance differences were determined using two-way ANOVA.
