# Supplementary material for: Restricting efferocytosis pathway to CD91 via phosphatidylserine-targeting chimeric protein augments antitumor immune responses
Source: Mol Ther. 2026 May 26;34(8):4729–42. doi: 10.1016/j.ymthe.2026.05.013 (PMC13464154; doi:10.1016/j.ymthe.2026.05.013)
Supplement: Supplementary file 2 [file mmc2.pdf]

Molecular Therapy
Original Article

Restricting efferocytosis pathway to CD91 via phosphatidylserine-targeting chimeric protein augments antitumor immune responses

Yu Mizote,[1,2,6] Hiroki Nishita,[3,4,6] Kazuki Hashiba,[4] Chisa Okuma,[4] Sachiko Akane,[4] Kenjiro Minomi,[4] and Hideaki Tahara[1,3,5]

[1]Department of Cancer Drug Discovery and Development, Research Center, Osaka International Cancer Institute, 3-1-69 Otemae, Chuo-ku, Osaka 541-8567, Japan; [2]Department of Cancer Immunotherapy, Research Center, Osaka International Cancer Institute, 3-1-69 Otemae, Chuo-ku, Osaka 541-8567, Japan; [3]Nitto joint Research Department for Nucleic Acid Medicine, Research Center, Osaka International Cancer Institute, 3-1-69 Otemae, Chuo-ku, Osaka 541-8567, Japan; [4]Nucleic Acid Medicine Business Division, Corporate Technology Sector, Nitto Denko Corporation, 1-1-2 Shimohozumi, Ibaraki, Osaka 567-8680, Japan; [5]Center for Clinical Research, Osaka International Cancer Institute, 3-1-69 Otemae, Chuo-ku, Osaka 541-8567, Japan

Phosphatidylserine is a representative eat-me signal on the outer membrane of apoptotic cells promoting phagocytosis/efferocytosis. It is recognized by various molecules, such as MFG-E8, which produce an immune-tolerizing environment. In order to develop immunological treatments targeting this mechanism, we designed a novel chimeric protein that consists of a phosphatidylserine-recognition domain from MFG-E8 and receptor-associated protein (RAP), which is a ligand of CD91 known as an immunostimulatory phagocytic receptor. This chimeric protein, PStRAP, can theoretically link phosphatidylserine-exposed apoptotic cells to phagocytes not through authentic immunosuppression but through an immunostimulative route via CD91. The inoculation of dying tumor cells expressing PStRAP, which served as an immunogen, induced potent adaptive immune responses against parental tumor cells in murine tumor models. The presence of PStRAP increased the phagocytosis of apoptotic cells *in vitro* and cross-priming to cytotoxic T-lymphocytes *in vivo* compared with the absence of it. Furthermore, PStRAP protein was supplied systemically by intravenous injection of PStRAP mRNA contained in lipid nanoparticles and along with cytotoxic drug to treat tumor-bearing mice. PStRAP significantly enhanced antitumor effects of cytotoxic drug alone. These results suggest that PStRAP can induce CD91-mediated phagocytosis of the apoptotic tumor cells and initiate meaningful adaptive immune responses against the tumor cells.

INTRODUCTION

Tumor cells escape from immune responses misappropriating various systems equipped for normal cells to be protected from autoimmune responses and maintain homeostasis.[1] One of the pivotal mechanisms used for avoiding autoimmune responses is rapidly disposing apoptotic cells by phagocytosis, which is termed efferocytosis.[2] Exposure of phosphatidylserine (PS) to the outer membrane leaflet of apoptotic cells is known not only to be an evolutionarily conserved pathway to prompt efferocytosis[3] but also can be considered to be an immune checkpoint.[4] Milk fat globule-epidermal growth factor-factor 8 (MFG-E8) is one of the PS-related molecules that is involved in efferocytosis.[5] MFG-E8 is a secreted molecule that bridges PS exposed on apoptotic cells and $\alpha_v\beta_{3/5}$ integrin on phagocytes. It prompts the rapid engulfment of the apoptotic cells by phagocytes, thereby inducing immune suppression in both normal and cancer cells.[6,7]

We have been trying to develop immunotherapy against cancer focusing on the PS/MFG-E8 pathway. In our previous study, immunological evasion of the tumor cells can be prevented via blocking the binding of MFG-E8 to integrins using an antibody targeting the region with RGD motif of the MFG-E8.[8] Our findings suggested that treatment using anti-MFG-E8 Ab with the fragment crystallizable (Fc) region can switch the pathway of efferocytosis from an immunosuppressive one through PS/MFG-E8/integrins to the immunostimulatory one through PS/MFG-E8/antibody/Fcγ receptors. Based on this proofed concept of the treatment, we initiated the study to use MFG-E8 itself combined with receptor-associated protein (RAP) instead of using anti-MFG-E8 Ab to access directly to PS. RAP is also known as LRPAP1, which is a chaperone protein in the endoplasmic reticulum (ER) and firmly binds to CD91.[9] CD91 is also known as low-density lipoprotein receptor-related protein 1 (LRP1) that acts as an endocytic receptor for various ligands including heat shock proteins (HSPs), such as gp96, hsp70, and calreticulin (CRT). When tumor cells undergo apoptosis with CRT

Received 8 October 2025; accepted 12 May 2026;
https://doi.org/10.1016/j.ymthe.2026.05.013.

[6]These authors contributed equally

Correspondence: Yu Mizote, Department of Cancer Drug Discovery and Development, Research Center, Osaka International Cancer Institute, 3-1-69 Otemae, Chuo-ku, Osaka 541-8567, Japan.
E-mail: mizote-yu@oici.jp

Published by Elsevier Inc. on behalf of The American Society of Gene and Cell Therapy.
This is an open access article under the CC BY license (http://creativecommons.org/licenses/by/4.0/).

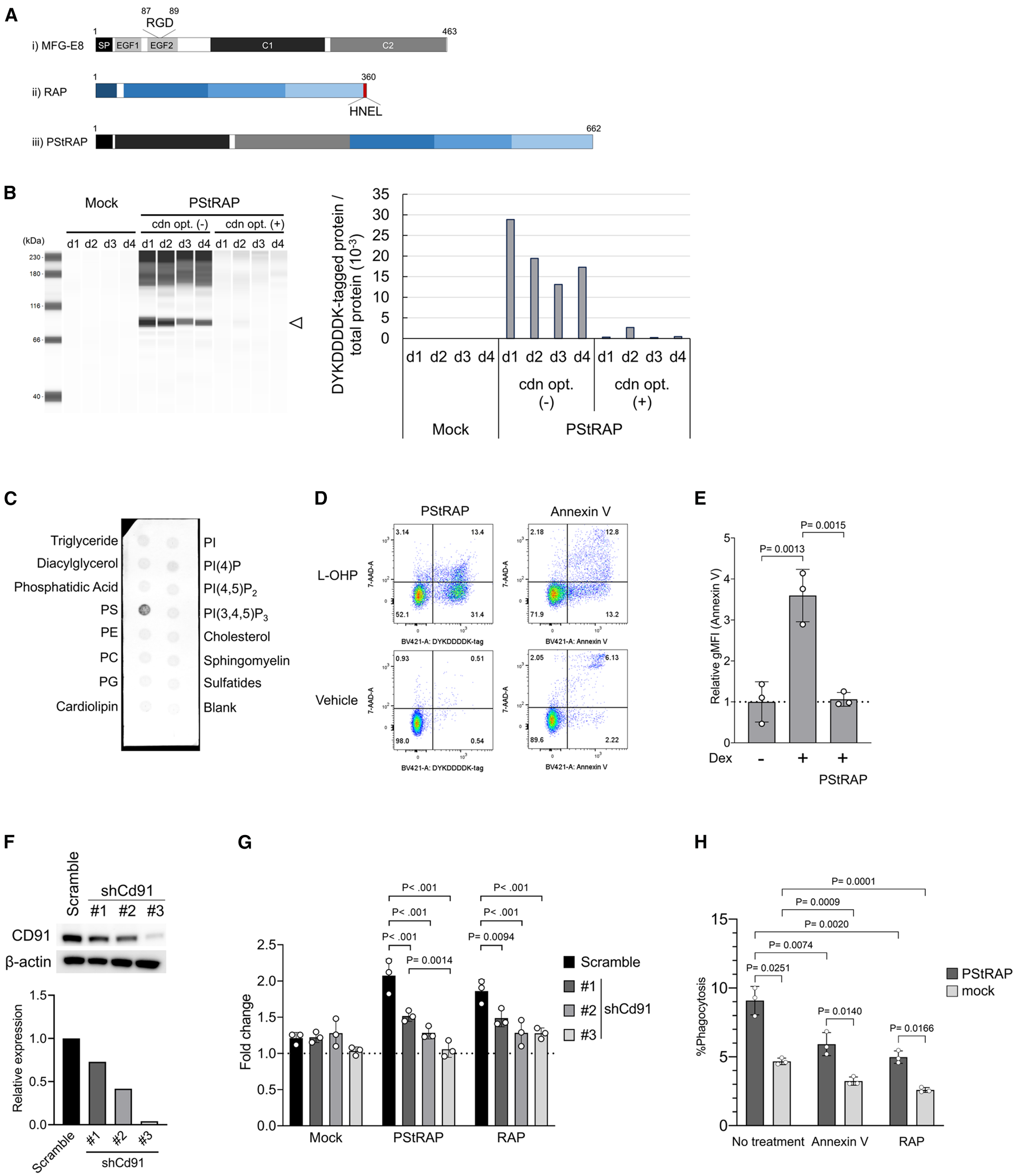

Figure 1. Structure and function of PStRAP

(A) The structures of MFG-E8 (i), RAP (ii), and PStRAP (iii) are depicted. RAP has tetrapeptide sequence HNEL at the C terminus for retaining in the ER, and PStRAP replaces this sequence to DYKDDDDK tag. Full-length sequence of PStRAP is shown in Figure S1A. SP, signal peptide. (B) PStRAP was detected using the capillary electrophoresis immunoblotting with anti-DYKDDDDK tag antibody from the culture supernatants of murine primary hepatocytes after mRNA transfection. Gel-like image view of

(legend continued on next page)

externalized, only the apoptotic tumor cells with such condition are engulfed via CD91. As a result of these events, tumor antigens are presented on dendritic cells (DCs) and induce adaptive immune responses.[10–13] We have chosen CD91 pathway instead of Fcγ receptors in this study, since some Fcγ receptors, such as FcγRIIB, do not necessarily induce activation of immune responses.[14]

We have shown that switching the pathway of efferocytosis using a chimeric protein, consisting of PS-binding domain of MFG-E8 and RAP, can induce potent antitumor immune responses when combined with various types of cancer treatment that cause apoptosis of the tumor cells.

RESULTS

Design and function of PStRAP

We designed a chimeric protein possessing MFG-E8-derived PS-binding and RAP-derived CD91-binding domain, termed PStRAP (PS-targeted RAP) (Figures 1A and S1A). The MFG-E8-derived PS-binding domain contains not only C2 segment, which binds to PS in a calcium ion-independent manner,[15,16] but also C1 segment. Recognizing apoptotic cells requires both the C1 and C2 segments of F5/8-type C domain of MFG-E8 in mouse.[5] The RAP-derived CD91-binding domain contains three segments (D1, D2, and D3) of RAP but not the tetrapeptide sequence HNEL at C terminus required to retain within the ER (Figure 1A(ii)).[17] The D3 domain primarily binds to CD91 and mediates endocytosis. However, the D1 and D2 domains also bind to CD91.[18] Plasmids were generated to express and utilize the chimeric protein. The protein was expressed by gene transfer of the plasmid or mRNAs that were synthesized by *in vitro* transcription (IVT) (Figure 1B).

The PStRAP protein was secreted from murine primary hepatocytes through transfecting the synthesized PStRAP mRNA. The PStRAP expression was detected in cell culture supernatant using a DYKDDDDK tag added to C terminus as a marker. We produced template DNA for IVT with the codons theoretically optimized (21.2% base substitution) to increase the protein expression level and to suppress dsRNA production. However, the expression level of the modified template DNA was substantially lower than that of the original sequence (Figures 1B and S1A). Therefore, we used template DNA with the original sequence in this study.

Membrane lipid array was performed on the culture supernatants obtained from murine primary hepatocytes one day after mRNA transfection to examine the specificity of the chimeric protein. The secreted PStRAP had bound to PS but not to other membrane lipids (Figure 1C). PStRAP was able to bind to the apoptotic MCA205 (fibrosarcoma) cells treated with oxaliplatin (L-OHP; 500 μM), but not to the viable cells (Figure 1D). Furthermore, PStRAP showed competitive inhibition against annexin V binding to PS of apoptotic thymocytes (Figures 1E and S2). These results indicate that PStRAP expressed in our system was capable of binding to PS on the apoptotic cells with certain specificity.

We also examined the function of PStRAP using murine macrophage cell line, RAW264.7, reported to express CD91, which activates NF-κB upon binding to several HSPs, such as gp96, hsp70, and CRT.[19] We thoroughly examined the function of PStRAP by producing RAW264.7 cells with different levels of CD91 knockdown using three types of shRNA (Figures 1F and S3). Similar to other HSPs, we confirmed that NF-κB was activated with PStRAP and RAP at the levels associated with the levels of CD91 expression (Figure 1G). Furthermore, efferocytosis efficiency was significantly increased with the presence of PStRAP compared with its absence, and the effect was attenuated by the addition of either annexin V or RAP (Figures 1H and S4). These results suggested that PStRAP can serve as a mediator for efferocytosis through recognizing PS and CD91.

PStRAP induced adaptive antitumor immune responses

We evaluated whether PStRAP induces adaptive antitumor immune responses *in vivo* as designed by immunizing tumor cell lines stably expressing PStRAP that was transfected with PStRAP DNA without the DYKDDDDK tag using a retrovirus-expressing vector. Mitomycin C (MMC)-pulsed transfectants were subcutaneously inoculated as an immunogen at the flank of mice. Under this treatment condition, although tumor cells are viable at the time of inoculation (3 h), more than 70% undergo apoptosis by 24 h, and nearly all cells

anti-DYKDDDDK tag antibody responses (left). Arrowhead indicates PStRAP with a predicted size of 76.4 kDa. The amounts of DYKDDDDK-tagged protein normalized to the total protein content (right). d, day; cdn opt, codon optimization. (C) Membrane lipid array was performed on the culture supernatants from the murine primary hepatocytes one day after mRNA transfection. PI, phosphatidylinositol; PE, phosphatidylethanolamine; PC, phosphatidylcholine; PG, phosphatidylglycerol. (D) Oxaliplatin (L-OHP; 500 μM) or vehicle-pretreated MCA205 cells were stained with PStRAP and BV421-conjugated anti-DYKDDDDK tag antibody or BV421-conjugated annexin V and counter stained with 7-AAD. (E) Thymocytes pretreated with dexamethasone (Dex; 10 μM) were stained with BV421-conjugated annexin V in the presence of PStRAP (n = 3/group). Relative geometric mean fluorescent intensity (gMFI) based on the Dex untreated group is shown. The gating strategy, individual plots, and gMFI values are shown in Figure S2. Error bars show mean (SD). Significance differences were determined using one-way ANOVA followed by post hoc Tukey's test. (F) CD91 expression level in RAW264.7 cell sublines transfected with shRNA was detected by immunoblotting (top). Pictures of the uncropped membrane are shown in Figure S3. The relative optical density was calculated using the optical density of the scramble shRNA transfectant as a reference (bottom). (G) The luciferase secreted upon NF-κB activation was measured in the culture supernatants from the RAW264.7 cell sublines 1 hour after stimulation with PStRAP or recombinant RAP protein (n = 3/group). The fold change was calculated using the luciferase level in the culture supernatants without stimulation as a reference. Error bars show mean (SD). Significance differences were determined using two-way ANOVA followed by post hoc Tukey's test. (H) RAW264.7 was cultured with CypHer5E-labeled thymocytes that had been pretreated with Dex (10 μM) for 4 h, in conditioning medium containing PStRAP- or mock-supernatant for 2 h. For the competitive assay, either annexin V or RAP protein was added to the medium during co-culturing. The CypHer5E-positive RAW264.7 cells were detected using flow cytometer, and the phagocytosis rate was calculated (n = 3/group). The gating strategy and individual plots are shown in Figure S4. Error bars show mean (SD). Significance differences were determined using an unpaired Welch's t test.

were dead by 48 h (Figure S5A). The relative expression level of 111 soluble proteins secreted into the culture supernatant was determined. Although 13 secreted factors were detected in viable cells, there was no change related to PStRAP expression. The MMC pulse caused some factors to either remain the same or decrease 24 h later; however, none of the factors increased as a result of the treatment. Similar profiles of soluble factors were shown between PStRAP-expressing and non-expressing MMC-pulsed cells (Figure S5B). Viable parental cells were subcutaneously transplanted at the opposite flank 7 days later, and then the resulting antitumor immune responses were evaluated (Figure 2A). Consistent with the results of our previous study,[7] the tumor growth in the mice inoculated with 5×10^5 cells of MMC-pulsed tumor cells were significantly suppressed compared with that in the mice inoculated with vehicle. The effect of tumor growth suppression was significantly enhanced by the inoculation of same number of MCA205 cells expressing PStRAP (Figure 2B). The significant enhancement of the growth suppression was also observed in a different tumor model using another syngeneic tumor cell line model (colon adenocarcinoma MC38) as well (Figure 2C).

Tumor growth suppression was not observed in the nude mice deficient in adaptive immunity, indicating that the tumor growth suppression was associated with the induction of adaptive antitumor immune responses due to the expression of PStRAP from dying tumor cells (Figure 2D). This interpretation was further supported by the observation that the splenocytes harvested from the mice inoculated with MC38-PStRAP secreted interferon gamma (IFNγ) and tumor necrosis factor alpha (TNFα) *in vitro* upon restimulation with peptides of mutation-derived neoepitopes (Figure 2E). These peptides are recognized by antigen-specific cytotoxic T-lymphocytes (CTLs) in the response to the MC38.[20,21] We further analyzed the effects of PStRAP on the cross-presentation efficiency by antigen-presenting cells (APCs) using ovalbumin (OVA)-expressing MCA205 and OT-I mice, which have a T-cell receptor that specifically recognizes the $OVA_{257\text{-}264}$ peptide bound to H-2K^b.[22] Mice were immunized with MCA205 transfectants pulsed with MMC, and the APCs were harvested from the tumor-draining lymph nodes one day later. The APCs were cocultured with CD8$^+$ cells from OT-I mice for three days. The amounts of IFNγ in the culture supernatants were detected only when PStRAP-expressing cells were inoculated (Figure 2F).

We also confirmed that PStRAP can induce adaptive immune responses through cell death caused by the endogenous immune response rather than by the drug. Instead of MMC-pulsed MCA205 cells, viable OVA-expressing MCA205 transfectants were subcutaneously inoculated at the flank of OT-I mice. Viable parental cells were subcutaneously transplanted at the opposite flank 7 days later. As OT-I mice harbor potent OVA-specific CTLs, the OVA-expressing cells are efficiently killed and completely rejected *in vivo* regardless of PStRAP expression in this tumor model. Therefore, in this setting, the OVA-expressing cells are assumed to primarily serve as a source of antigen during or following immune-mediated cell death, rather than serve as antigen-presenting live cells. Under this condition, tumor growth of parental cells was significantly suppressed in PStRAP-expressing group (Figure 2G). These results demonstrate that PStRAP efficiently induces adaptive immune responses in association with cell death, consistent with its role in enhancing immune priming from dying cells.

PStRAP requires recognition of both PS and CD91 in order to establish adaptive antitumor immune responses

After confirming PStRAPΔPS and PStRAPΔCD91 have no binding capabilities to their respective targets (Figure S6), we then examined the antitumor effects of inoculating with the dying tumor cells expressing these mutants. No significant antitumor effects were observed in the mice inoculated with dying MCA205 cells expressing these mutants (Figure 3A). This contrasts with the tumor behaviors observed in the mice inoculated with the dying MCA205 cells expressing PStRAP. Interestingly, the tumors in the mice treated either with PStRAPΔPS or PStRAPΔCD91 showed more rapid growth compared with the tumors in the mice inoculated with the cells without PStRAP expression (Figures 2B and 3A). The tumor growth suppression effect induced by PStRAP attenuated when both PStRAP and RAP were expressed in MCA205 cells used as an immunogen (Figures 3B and S7). To evaluate the cross-presentation efficacy *in vivo*, CFSE-labeled OT-I CD8$^+$ T-cells were adoptively transferred to wild-type mice. One day later, OVA-expressed MCA205 cells with or without PStRAP, or with both PStRAP and RAP, were inoculated to flanks of mice. Three days later, the draining lymph nodes were harvested and detected for dividing OT-I cells. The number of dividing OT-I cells increased in the presence of PStRAP compared with its absence, and this effect was eliminated by the presence of RAP (Figures 3C and S8).

It is conceivable that different types of PStRAP can be constructed using the other PS-binding molecules that are functionally similar to the C1C2 domain of MFG-E8. Thus, we incorporated Gla domain of growth arrest-specific 6 (Gas6) and T-cell immunoglobulin mucin protein 4 (Tim4), which have potent PS-binding capability, into PStRAP, respectively (PStRAP (Gas6) and PStRAP (Tim4)) (Figures 3D and S9A). Similar to MFG-E8, Gas6 is a secreted protein that opsonizes apoptotic cells.[23,24] PStRAP (Gas6) also induced antitumor effects similar to that of PStRAP using the C1C2 domain (Figure 3E). Although MFG-E8 and Tim4 are expressed by different subsets of macrophages, they both tightly bind to PS on apoptotic cells and enhance efferocytosis by phagocytes.[25,26] Furthermore, mice with these genes knocked out age-dependently develop an autoimmune disease.[27] Nevertheless, PStRAP (Tim4) expression was not associated with significant antitumor effects (Figure S9B). The strategy of capturing PS is effective, but the capture molecules should be selected with caution. These results confirm that the significant antitumor effects were induced by PStRAP functions that could bridge between PS and CD91 and promote the induction of tumor antigen-specific CTLs.

Systemic expressing PStRAP via intravenous injection of LNP/mRNA enhanced antitumor effect of cytotoxic drug

To confirm the effectiveness of PStRAP treatment in a therapeutic model, mRNA of PStRAP encapsulated in lipid nanoparticle

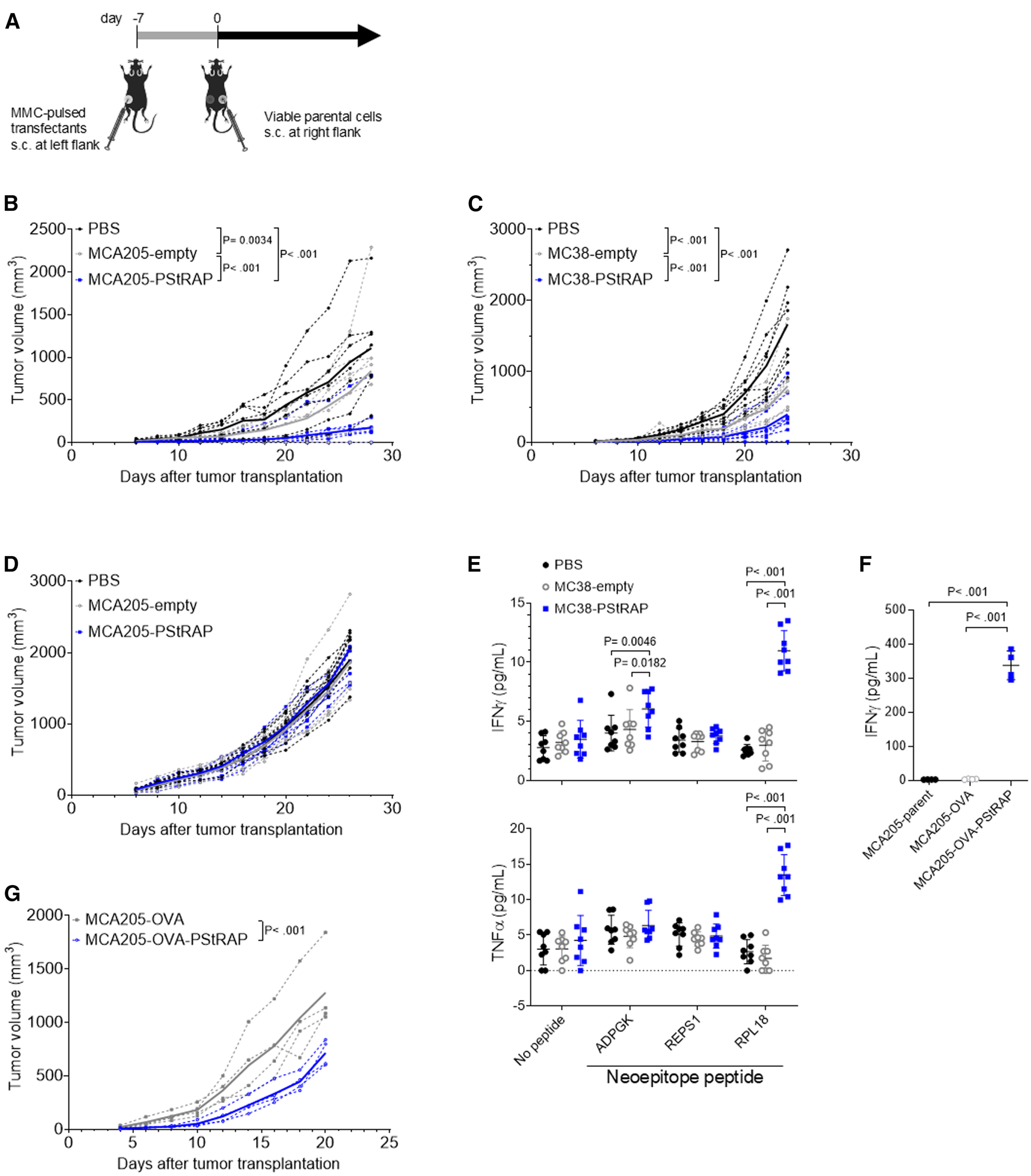

Figure 2. PStRAP enhanced cross-presentation and induced adaptive antitumor immune responses

(A–D) Each MMC-pulsed transfectant (5×10^5 cells) or PBS was subcutaneously inoculated into the left flank 7 days before subcutaneous transplantation of viable MCA205 (5×10^5 cells) (B and D) or MC38 (3×10^5 cells) (C) cells at the right flank of C57BL/6 mice (B and C) or nude mice (D), and then the tumor volumes were measured every other day (*n* = 8/group). Significance differences were determined using two-way ANOVA followed by post hoc Tukey's test. (E) The splenocytes were harvested from MC38-transplanted mice on day 26, as shown in (C). The splenocytes cultured with 10 μM of indicated peptide or no peptide for three days, the culture supernatants were collected, and the amounts of IFNγ and TNFα were measured using ELISA. Error bars show mean (SD). Significance differences were determined using two-way ANOVA followed by post hoc Tukey's test. (F) APCs were prepared from tumor-draining subiliac lymph node of mice (*n* = 4/group) one day after MMC-pulsed parental MCA205, MCA205-OVA, or MCA205-OVA-PStRAP cells (2×10^6 cells) subcutaneously inoculated at the right flank. CTLs that were purified from splenocytes of OT-I mice and the APCs were cocultured for three days followed by collection of the culture supernatants and measuring the amounts of IFNγ using ELISA. Error bars show mean (SD). Significance differences were determined using one-way ANOVA followed by post hoc Tukey's test. (G) MCA205-OVA or MCA205-OVA-PStRAP (5×10^5 cells) were subcutaneously inoculated into the left flank of the OT-I mice. Viable MCA205 (5×10^5 cells) cells were subcutaneously transplanted into the right flank seven days later, and then tumor volumes were measured every other day (*n* = 4/group). Significance differences were determined using two-way ANOVA.

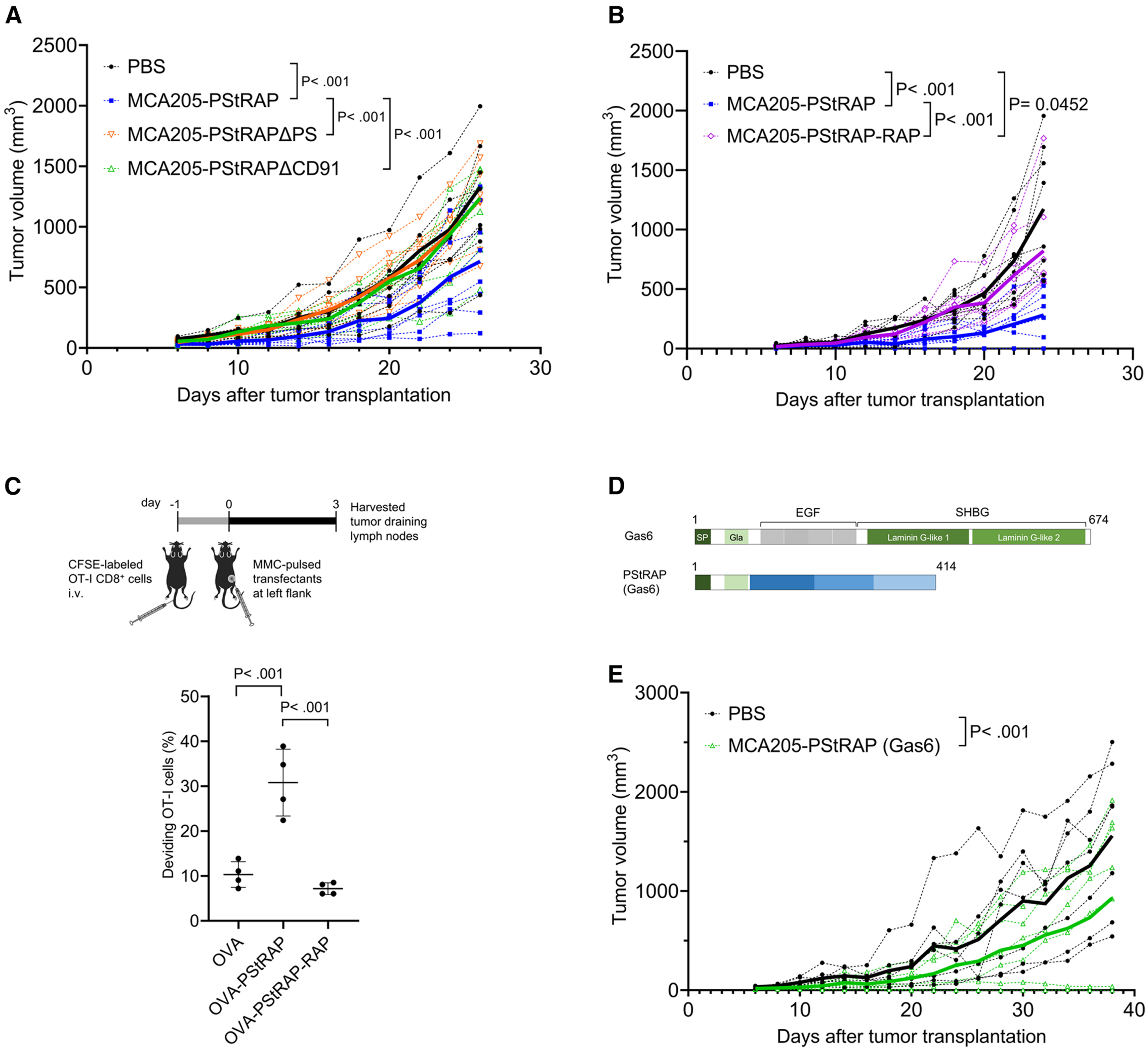

Figure 3. PS and CD91 recognition enable for PStRAP-induced adaptive immune responses

(A, B, and E) MMC-pulsed MCA205-PStRAP, its mutant (ΔPS or ΔCD91) (A), RAP co-expressing (B), or PStRAP (Gas6) (E) transfectants (5×10^5 cells), or PBS were subcutaneously inoculated into the left flank of the mice. Viable MCA205 (5×10^5 cells) cells were subcutaneously transplanted into the right flank seven days later, and then tumor volumes were measured every other day (n = 8/group). Significance differences were determined using two-way ANOVA followed by post hoc Tukey's test. (C) The effect of RAP on T-cell proliferation *in vivo* was examined. Wild-type C57BL/6 mice were adoptively transferred with CFSE-labeled OT-I cells, 1 day before immunization with MMC-pulsed MCA205-OVA with or without PStRAP and RAP expression. After three days, the draining subiliac lymph nodes were harvested and stained for TCR-Vβ5 and CD8 expression. The percentages of dividing OT-I cells are shown (n = 4/group). Error bars show mean (SD). Significance differences were determined using one-way ANOVA followed by post hoc Tukey's test. The gating strategy and individual histograms are shown in Figure S8. (D) The structures of Gas6 (top) and PStRAP (Gas6) (bottom) are depicted. SP, signal peptide; SHBG, sex hormone-binding globulin.

(LNP/PStRAP-mRNA) was administered intravenously. Intravenously administering LNP/mRNA is expected to be mainly delivered to the liver and translated into protein and then released in the systemic circulation. To confirm the *in vivo* delivery of this system, firefly luciferase (FLuc)-mRNA encapsulated in LNPs (LNP/Fluc-mRNA) were injected into tail veins of the mice. Several organs were harvested 6 or 24 h after administration, and the luminescence intensities were measured. The luminescence was detected in only the liver and 6 h after administration, and it was detectable but at decreased levels 24 h after administration (Figure S10A). PStRAP protein was detected in the serum 6 h after LNP/PStRAP-mRNA administration and remained detectable until five days after administration but at decreased levels (Figure 4A). The levels of alanine aminotransferase (ALT) and aspartate aminotransferase (AST), and the weight of the administrated mice did not differ from those of the vehicle-administrated mice (Figures S10B and S10C). These results suggest that intravenously injecting this dose of LNP/PStRAP-mRNA showed no serious toxicity and did not induce liver injury (Figure S10C). To investigate the antitumor effects of LNP/PStRAP-mRNA treatment in an established tumor model, mice

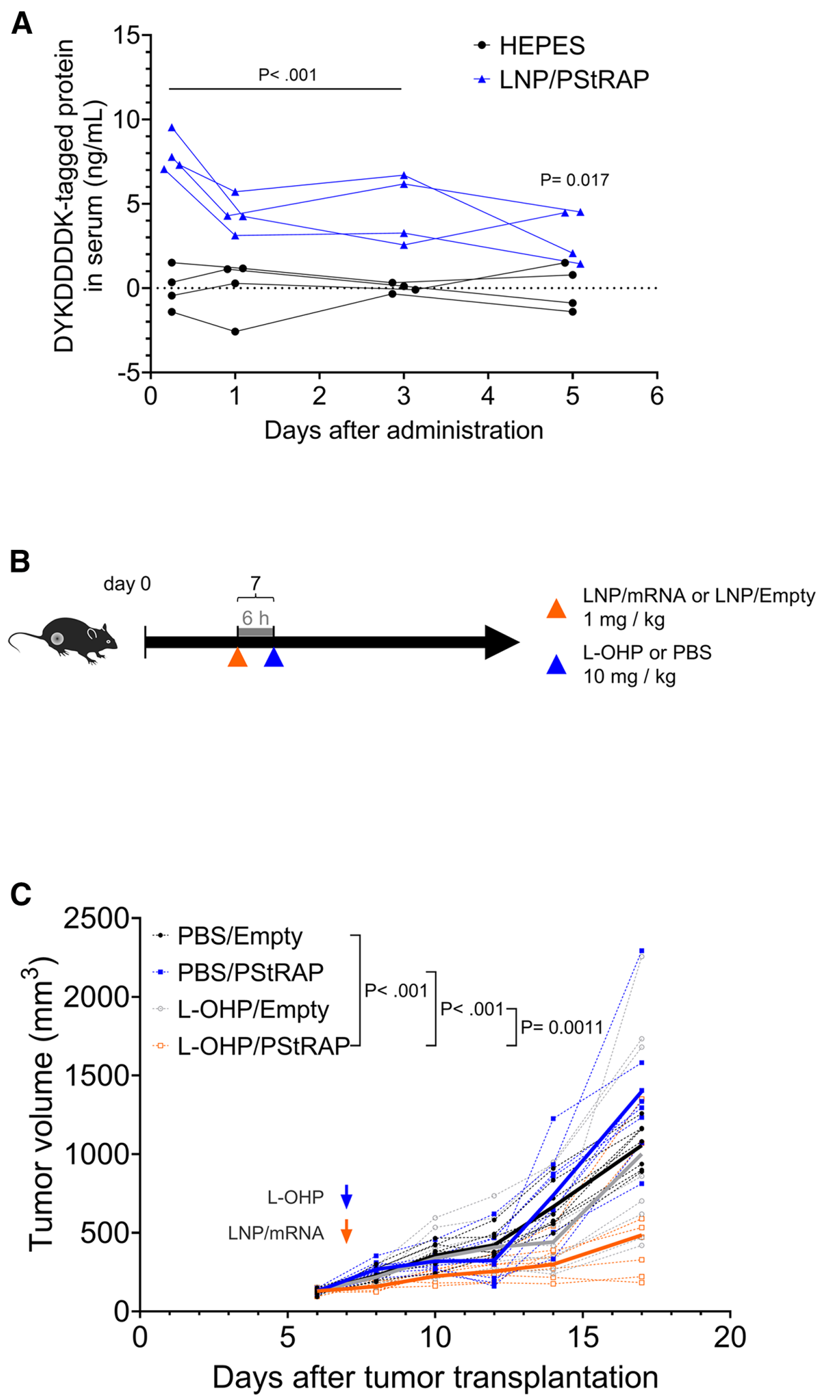

Figure 4. Anticancer therapeutic effects of PStRAP systemically expressed via LNP/mRNA inoculation

(A) Blood was collected from the mice at the indicated time points after intravenous LNP/mRNA (1 mg/kg) or HEPES injection. The amounts of PStRAP in serum were measured via detecting DYKDDDDK-tagged proteins. Significance differences were determined using an unpaired Welch's *t* test at each time point. (B and C) MCA205-bearing mice were intravenously injected on day 7 with LNPs containing mRNA encoding PStRAP (1 mg/kg) or empty LNP 6 h prior to intraperitoneally administrating L-OHP (10 mg/kg) or PBS on the same day. Significance differences were determined using two-way ANOVA followed by post hoc Tukey's test.

bearing day 7 tumors were treated with LNP/PStRAP-mRNA combined with L-OHP (Figure 4B). L-OHP induced apoptosis in MCA205, which was recognized by PStRAP *in vitro* (Figure 1D). Although administrating L-OHP alone or LNP/PStRAP-mRNA alone did not affect tumor growth compared with that in the vehicle-administrated group, combined L-OHP and LNP/PStRAP-mRNA treatment significantly suppressed tumor growth (Figure 4C). These results suggest that LNP/PStRAP-mRNA treatment can be used to enhance antitumor effects when combining with the treatment that induce apoptosis in established tumors.

DISCUSSION

It has been shown by us and other investigators that antitumor effects can be elicited by the treatment with antibodies against PS-bridging molecules including MFG-E8 or β2-glycoprotein 1.[8,28] Since these antibodies have been used to block the functions related to PS by targeting the PS-bridging molecules that spontaneously bind to PS, various other PS-bridging molecules and PS receptors can still bind to PS and modulate immune functions.[4,29] One of the strategies to improve this situation is to develop treatments using molecules with high binding affinities to PS to impede the binding of other molecules. In murine neuroblastoma model where viable tumor cells constitutively expose PS on their surface to suppress T-cell functions, the masking of PS with annexin V can induce antitumor immune responses.[30] However, annexin V requires a higher percentage of PS on the external plasma membrane for binding compared with MFG-E8.[31,32] As such, we have initiated the study using C1C2 domain of MFG-E8, instead of anti-MFG-E8 antibody, to directly access the PS of apoptotic cells. We designed PStRAP, a chimeric protein, to modulate the immunoregulatory PS-related pathway with the C1C2 domain of MFG-E8. Using PStRAP, we have successfully shown in two different syngeneic tumor cell line models (fibrosarcoma MCA205 and colon adenocarcinoma MC38) that the tumor growth in the mice inoculated with PStRAP-expressing dying tumor cells was significantly suppressed when compared with that in the mice inoculated with the dying tumor cells transfected with empty vector (Figures 2B and 2C). Since this tumor growth suppression was shown to be associated with the promotion of tumor-specific immunity (Figures 2D–2F), the concept of our strategy appears to be proven.

Interestingly, the tumors in the mice treated either with PStRAPΔPS or PStRAPΔCD91 showed more rapid growth compared with the tumors in the mice treated with cells without PStRAP expression (Figures 2B and 3A). The PStRAP lacking CD91 binding ability (PStRAPΔCD91) might play the immunoregulatory roles similar to the MFG-E8 mutant lacking integrin-binding capacity (e.g., C1C2 domain only, RGD motif replaced with RGE), which prevents the engulfment of apoptotic cells and suppresses subsequent antigen cross-presentation as shown in the study reported by the other group.[6] Regarding PStRAP lacking PS-binding ability (PStRAPΔPS), it might behave similarly to the RAP, which inhibited usual efferocytosis by RAW264.7 cells *in vitro* (Figure 1H) and could suppress CD91-dependent pathways and prevent the induction of adaptive immune responses.[33] These results support that it is necessary to express PStRAP in a complete form to induce positive antitumor immune responses.

It is conceivable that alternative PS-binding molecules could be used to construct PStRAP variants functionally similar to the C1C2 domain of MFG-E8. One candidate molecule is Gas6, which binds

PS via its Gla domain. Although full-length Gas6 also contains a sex hormone-binding globulin (SHBG)-like domain that engages three receptor tyrosine kinases of the TAM (Tyro3, Axl, and Mertk) family, our construct utilized only the Gla domain and therefore lacks TAM receptor-binding capacity (Figure 3D).[24,34] Gas6 binds to TAM receptors on APCs during efferocytosis to promote phagocytosis and transmit immunosuppressive signals that prevent autoimmune responses.[35,36] Another candidate molecule is Tim4, which acts as a PS receptor and cooperates with integrins to promote efferocytosis.[37] In cancer situations, Tim4 is reported to be expressed on non-small cell lung cancer and interacts with the $\alpha_v\beta_3$ integrin to promoted tumor growth.[38] Thus, we incorporated Tim4, a molecule with potent PS-binding capability, into PStRAP (PStRAP (Tim4)) (Figure S9A). Although Tim4 binds to PS similarly to MFG-E8 and Gas6, PStRAP (Tim4) was unable to induce significant antitumor effects (Figure S9B). Notably, the PS-binding region of Tim4 resides within an IgV-like domain that also contains an RGD motif implicated in integrin interactions (Figure S9A).[39] It is possible that integrin engagement or structural constraints within the Tim4 domain alter the orientation or signaling context of PS presentation, thereby limiting its capacity to promote immunogenic antigen processing. Therefore, PS-binding molecules with other functions but located in the same domain may not be suitable for our strategy.

The data shown in our current study suggest that PStRAP promotes the uptake of PS-exposing dying cells, leading to antigen processing and cross-presentation by recipient APCs. However, it is possible that extracellular vesicles (EVs) that express PS have roles in our system, since EVs have been shown to be involved in various types of immunological stimulation and regulation.[40–43] DC-derived EVs have been reported to express peptide-MHC complexes together with co-stimulatory molecules and can directly activate T-cells.[44] In contrast, tumor-derived EVs have been reported to induce T cell dysfunction by modulating the tumor microenvironment.[45,46] Furthermore, they may also serve as a source of antigen for uptake and processing by recipient APCs.[47] Although we cannot exclude a contribution of EV-associated antigen to the results of the current study, it appears unlikely that EVs alone account for the observed effects, since similar responses were not observed in our experimental setting using the viable cells (Figure 4C). Further studies might lead to a better understanding of the roles of EVs in our system.

CD91 is a highly endocytic and rapidly recycling scavenger receptor that constitutively undergoes ligand-induced internalization followed by efficient return to the plasma membrane.[48,49] Although RAP is a well-characterized CD91 ligand that can induce transient receptor internalization,[50] due to the rapid recycling kinetics of CD91, such internalization does not necessarily lead to sustained surface depletion.[49,51] In the present study, PStRAP may exist in both soluble and PS-bound forms depending on the availability of exposed PS. In the context of apoptotic tumor cells, PS exposure likely promotes local enrichment of PStRAP at the dying cell surface, thereby facilitating spatially restricted CD91 engagement. Under these conditions, PStRAP is less likely to function as a systemic excess ligand causing global receptor downregulation. Rather than quantitatively altering CD91 surface levels, our data support a model in which PStRAP qualitatively redirects CD91-mediated processing of dying tumor cells toward an immunogenic outcome. Importantly, we did not observe phenotypic evidence of impaired apoptotic cell clearance *in vivo* under the experimental conditions used. Together, these findings support a mechanism in which PStRAP enhances the immunogenic context of CD91 engagement without disrupting physiological receptor availability. Future studies examining the kinetics of CD91 trafficking in response to PStRAP will further refine this mechanistic framework.

In this report, we developed a chimeric protein termed PStRAP to modulate not only the immunoregulatory PS-related pathway but also the immunostimulatory CD91-related pathway.[52] CD91 is expressed on professional APCs, including DCs and macrophages, where it mediates endocytic uptake of extracellular ligands and contributes to antigen cross-presentation.[10,53] CD91-expressing DCs have been identified as a subset of professional APCs capable of enhancing adaptive immune responses to CD91-targeted antigens.[54] In the context of tumor immunity, CD91-dependent interactions have been shown to be required for the establishment of effective antitumor immune responses.[33] Additionally, CD91-gp96 signaling has been demonstrated to confer cross-priming capacity to multiple APC subsets during immune responses to emerging tumors.[55] Within this established framework, our findings support a model in which PStRAP enhances the immunogenic processing of apoptotic tumor cells by promoting CD91-mediated antigen acquisition at the dying cell-APC interface. Since the results of cytokine profile analysis for dying tumor cells did not reveal apparent or consistent changes in inflammatory mediator production in the presence or absence of PStRAP (Figure S5B), it would be difficult to speculate the mechanism of the PStRAP-related antitumor effects would be primarily driven by secondary soluble factors. In addition to the examination of enhanced apoptotic cell uptake, we evaluated cross-presentation function using antigen-specific $CD8^+$ T-cells. The presence of PStRAP significantly increased $CD8^+$ T-cell proliferation, indicating enhanced cross-presentation efficiency (Figures 1H and 3C). These data indicate that PStRAP does not primarily amplify adaptive immunity through nonspecific inflammatory activation but rather augments receptor-mediated antigen acquisition. Given that apoptotic cells alone induce a baseline adaptive response (Figures 2B and 2C), the enhanced uptake observed in the presence of PStRAP supports a model in which PStRAP qualitatively redirects or amplifies CD91-dependent antigen processing, thereby significantly strengthening antitumor immunity without broadly reshaping the inflammatory milieu of dying tumor cells.

The mode of tumor cell death that causes subsequent adaptive antitumor immune responses has been defined as immunogenic cell death (ICD), and several treatments for cancer have been shown to induce ICD.[56] For example, cell death involving cell surface exposure of CRT is considered a hallmark of ICD. However, multiple types of

cancer do not respond to chemotherapeutic agents that induce ICD. In contrast, PStRAP was able to promote potent adaptive antitumor immune responses even when cell death was induced by MMC, which is a representative non-ICD inducer (Figures 2B and 2C). PStRAP also was able to enhance the adaptive immune responses resulting from tumor cell death caused by CTLs occurring *in vivo* (Figure 2G). This suggests that modulation of CD91-mediated uptake represents a strategy to amplify antitumor immunity independent of the intrinsic immunogenicity of tumor cell death. In addition, recent reports showed that PS exposure to the outer membrane leaflet of cell surface also occurs in programmed necrosis, such as necroptosis and pyroptosis.[57,58] PStRAP can induce the antitumor immune responses independent of the mode of cell death and may be useful when combined with various therapies. They include not only conventional chemotherapy and radiotherapy but also other types of treatments, such as CAR-T therapy and oncolytic virus therapy.

In current study, when mRNA of PStRAP encapsulated in lipid nanoparticle (LNP/PStRAP-mRNA) was intravenously administered, mice bearing day 7 established tumors were successfully treated with LNP/PStRAP-mRNA combined with the chemotherapy (Figure 4C). These results suggest that also systemically administrating PStRAP protein can be an effective therapy when combined with other types of treatments that induce tumor apoptosis including existing immune checkpoint inhibitors.

Collectively, our findings demonstrate that manipulating the efferocytosis pathway may lead to the activation of potent adaptive antitumor immune responses, and PStRAP may serve as a potentiator for existing various types of cancer therapy.

MATERIALS AND METHODS

Mice

The C57BL/6NJ and CAnN.Cg-*Foxn1*$^{nu/nu}$/CrlCrlj (nude) mice were obtained from Charles River Laboratories Japan (Kanagawa, Japan). The C57BL/6-Tg (TcraTcrb) 1100Mjb (OT-I) mice were provided by Dr. W.R. Heath (Walter & Eliza Hall Institute of Medical Research, Melbourne, Australia).[22] All animal experiments were approved by the Animal Care and Use Committee of the Osaka International Cancer Institute (approval numbers: 25030325, 24021909, and 23022116). The experiments were conducted under specific pathogen-free conditions, reproduced more than twice, and representative data were depicted.

Cells and transfectants

MCA205 (murine fibrosarcoma) and MC38 (murine colon adenocarcinoma) cell lines were provided by Dr. S.A. Rosenberg (National Cancer Institute, Bethesda, MD). These cell lines were authenticated via short tandem repeat profiling using an FTA Sample Collection Kit for Mouse Cell Authentication Service (137-XV, American Type Culture Collection: ATCC, Manassas, VA). RAW264.7 (murine leukemia macrophage) cell lines were purchased from ATCC (TIB-71). Tumor cell lines and primary hepatocytes were cultured in Dulbecco's modified Eagle's medium (DMEM), high-glucose, GlutaMAX Supplement (10566, Thermo Fisher Scientific, Waltham, MA) supplemented with 1% penicillin-streptomycin (15140, Thermo Fisher Scientific), and 10% fetal bovine serum (35-079-CV, Corning Inc., Corning, NY) (complete DMEM) at 37°C in a 5% CO_2 atmosphere.

Primary cells from thymus, spleen, and lymph nodes were cultured in Roswell Park Memorial Institute (RPMI) 1640, GlutaMAX Supplement (61870, Thermo Fisher Scientific) supplemented with 1% penicillin-streptomycin, 50 μM of 2-Mercaptoethanol (21985023, Thermo Fisher Scientific), and 10% fetal bovine serum (complete RPMI) at 37°C in a 5% CO_2 atmosphere.

OVA-expressing MCA205 cells (MCA205-OVA) were established following a previously reported method.[7] PStRAP, its mutants, and PA-tagged RAP at the C terminus were expressed via pMXs retroviral transfection (RTV-012, Cell Biolabs, San Diego, CA). Using the information on MFG-E8 and RAP on binding to PS and CD91, respectively, we constructed PStRAP mutants that were functionally defective in binding to PS or CD91. PStRAPΔPS was produced by replacing W333, N334, L335, F388, and G389 in the MFG-E8 region with alanine and was unable to bind to PS.[59] PStRAPΔCD91 was produced by replacing K293 and K307 in the RAP region with alanine and was unable to bind to CD91 (Figure S1A).[18]

For knockdown of CD91 (GenBank: NM_008512.2), short hairpin RNA (shRNA) expressing lentivirus made from pLV vector was used. Three *Cd91*-targeted shRNAs (#1: AGGCGCCTGTGTGGTCA ATAA, #2: CGACAACGACTGCGGAGATAA, or #3: CGGAGTCA CTTACATCAATAA) and one scramble shRNA control (CCTAA GGTTAAGTCGCCCTCG) containing lentivirus particles were purchased from VectorBuilder (Chicago, IL), which were infected to RAW264.7 cells. To detect NF-κB activation, pNiFty2-N-Lucia-Blasti (pnf2b-lc, InvivoGen, San Diego, CA) was transfected into shRNA-expressing RAW264.7 cell sublines.

mRNA synthesis and LNP formulation

DNA synthesis, codon optimization (using GenSmart Optimization version Beta 1.0), cloning, and plasmid preparation were provided by GenScript (Piscataway NJ). The plasmid DNA was linearized by the restriction enzyme, BspQI (#R0712, New England Biolabs, Ipswich, MA). mRNA was synthesized *in vitro* on linearized plasmid, which consisted of a T7 promoter, a 5′-untranslated region (UTR) containing a Kozak sequence, a coding region, a 3′-UTR, and a 120-nt poly(A) tail, using a MEGAscript T7 Transcription Kit (#AM1330, Thermo Fisher Scientific) following the instructions provided by the manufacturer (Figure S1B). Uridine 5′-triphosphate was fully substituted with 5-methoxyuridine-5′-triphosphate (5moU; #*N*-1093, TriLink Biotechnologies, San Diego, CA), and the 5′ capping of the IVT mRNA was cotranscriptionally incorporated using the CleanCap Reagent AG (#*N*-7113-5, TriLink Biotechnologies). IVT mRNA was purified using high-performance liquid chromatography (Akta explorer 100, cytiva, Wilmington, DE) with a CIMmultus Oligo dT18 1 mL Monolithic Column (C12 Linker) (2-μm channels)

(#311.1219–2, Sartorius, Göttingen, Germany). The collected fractions were concentrated using an Amicon Ultra-15 centrifugal filter unit (30 kDa) (UFC9030, Merck KGaA, Darmstadt, Germany). The integrity of the IVT mRNAs was analyzed using capillary electrophoresis.

For formulation of mRNA-encapsulated LNPs (LNP/mRNA), NanoAssemblr Benchtop Systems (Precision NanoSystems, Vancouver, Canada) was used as previously described.[60,61] Briefly, the lipid mixture with mRNA (or without mRNA for control) was rapidly mixed at an N/P ratio of 6 in citrate buffer (50 mM, pH 3.5). These LNP solutions were ultrafiltered using Amicon Ultra-15 centrifugal filter units (100 kDa) (UFC9100, Merck KGaA) for buffer exchange with 20 mM HEPES (4-(2-hydroxyethyl)piperazine-1-ethanesulfonic acid) 9% sucrose buffer (pH 7.45). LNPs containing ALC-0315, an ionizable cationic lipid also used in the approved SARS-CoV-2 mRNA vaccine,[62] were employed to deliver mRNA to the liver.

Hepatocyte purification and transfection

Single-cell hepatocytes were dissociated from fresh mouse liver using a Liver Perfusion Kit (130-128-030, Miltenyi Biotec, Bergisch Gladbach, Germany) and gentleMACS Octo Dissociator (130-134-029, Miltenyi Biotec) according to the instructions provided by the manufacturer. After the hepatocytes were purified, the primary hepatocytes were transfected with mRNA using Lipofectamine MessengerMAX Transfection Reagent (LMRNA, Thermo Fisher Scientific). Culture supernatants were collected daily for 4 days after transfection and cryopreserved until use.

Western blotting

Whole-cell lysates were prepared using RIPA Lysis Buffer containing a Halt Protease Inhibitor Cocktail (89900 and 87786, Thermo Fisher Scientific). Each cell lysate (1×10^5 cells per lane) was loaded on mPAGE 4–20% Bis-Tris Precast Gels (MP42G15, Merck KGaA) and separated using SDS-PAGE. The proteins in the gel were transferred to an Immobilon-P transfer membrane (IPVH07850, Merck KGaA), and the membrane was stained with LRP1 Recombinant Rabbit Monoclonal Antibody (clone SA0290, 1/1,000 dilution; MA5-31959, Thermo Fisher Scientific), Anti PA tag Rat Monoclonal Antibody (clone NZ-1, 1/1,000 dilution; 012–25863, FUJIFILM Wako, Osaka, Japan), or Anti β-Actin Monoclonal Antibody (clone 6D1, 1/10,000 dilution; 010–27841, FUJIFILM Wako). The membrane was then stained with Peroxidase-conjugated AffiniPureR Alpaca Anti-Rabbit IgG (H + L), Anti-Mouse IgG (H + L), or Donkey Anti-Rat IgG (H + L) (polyclonal, 1/10,000 dilution; 611-035-215, 615-035-214, and 712-035-153, respectively, Jackson ImmunoResearch, Philadelphia, PA). The enzymatic reaction was induced using a Clarity Western ECL Substrate (1705060, BIO-RAD Laboratories, Hercules, CA) and detected by a ChemiDoc Touch (BIO-RAD Laboratories).

The culture supernatants from PStRAP mRNA-transfected hepatocytes were analyzed using a Jess Simple Western system (ProteinSimple, San Jose, CA) according to the instructions provided by the manufacturer. The DYKDDDDK Tag Rabbit mAb (clone D6W5B, 1/50 dilution; #14793 Cell Signaling Technology, Danvers, MA) was used for detection.

Lipid-protein interaction assay

To assess the direct lipid-binding ability and specificity of PStRAP, a lipid-protein interaction assay was performed using Membrane Lipid Strips (P-6002, Echelon Biosciences, Salt Lake City, UT), according to the instructions provided by the manufacturer. Briefly, membranes were blocked in 3% bovine serum albumin (BSA) in PBS and then incubated for 1 h with the culture supernatants from the PStRAP mRNA-transfected hepatocytes. After washing the membranes, they were incubated for 1 h with 1/1,000 diluted DYKDDDDK Tag Rabbit mAb (clone D6W5B; #14793, Cell Signaling Technology) followed by incubation with 1/10,000 diluted Peroxidase-conjugated AffiniPureR Alpaca Anti-Rabbit IgG (H + L). The enzymatic reaction was induced using a Clarity Western ECL Substrate and detected by a ChemiDoc Touch.

Detecting PS with flow cytometry

MCA205 cells were induced to undergo apoptosis via treatment with 500 μM of L-OHP (O0372, Tokyo Chemical Industry: TCI, Tokyo, Japan) for 24 h. To confirm the state of MMC-pulsed cells, MCA205 was precultured with 500 μM of MMC (M2320, TCI) or vehicle (dimethyl sulfoxide) for 3 h. The cells were washed three times and then continued to be cultured, and they were harvested 3, 24, and 48 h after MMC treatment. The thymocytes from the C57BL/6NJ mice were cultivated with 10 μM of dexamethasone (Dex; D1961, TCI) in complete DMEM for 4 h.[5] The PS on the cell surface was detected using Brilliant Violet 421 (BV421) annexin V (640923, BioLegend, San Diego, CA), and dead cells were costained with 7-AAD (420403, BioLegend) or Helix NP NIR (425301, BioLegend). As a method to detect PS instead of annexin V, the apoptosis-induced cells were incubated with the culture supernatants from PStRAP- or mock-mRNA-transfected hepatocytes followed by staining with a BV421 anti-DYKDDDDK Tag antibody (clone L5; 637322, BioLegend). For the competitive assay, either PStRAP- or mock-supernatant was added during annexin V staining. The stained cells were detected using LSRFortessa X-20 (Becton, Dickinson and Company: BD, Franklin Lakes, NJ), and the results were analyzed using FlowJo v10.8.1 (BD).

NF-κB reporter assay

pNiFty2-N-Lucia-Blasti vector-transfected RAW264.7 cell sublines were seeded on a 96-well flat-bottom plate (353072, Corning Inc.) at a density of 1×10^5 cells per well. Cells were treated with the culture supernatants from PStRAP- or mock-mRNA-transfected hepatocytes, 40 μg/mL of recombinant mouse RAP protein (4480-LR-050, R&D Systems, Minneapolis, MN), or with no treatment in complete DMEM for 1 h. After treatment, 20 μL of the supernatant was transferred to a 96-well white plate (3912, Corning Inc.), and 50 μL of QUANTI-Luc 4 Lucia/Gaussia Flash solution (rep-qlc4lg, InvivoGen) was added. The luciferase activity was measured using

Infinite M Plex (Tecan, Männedorf, Switzerland). NF-κB activity was indicated as a fold change with respect to the activity in the untreated group as a reference.

Phagocytosis assay

The apoptosis-induced thymocytes from the C57BL/6NJ mice were cultivated with 10 μM of Dex in complete DMEM for 4 h and then labeled with 5 μM CypHer5E (PA15401, cytiva) in HBSS (Hanks' Balanced Salt Solution; 14175, Thermo Fisher Scientific) for 30 min at 37°C. The CypHer5E is a pH-sensitive dye that exhibits maximally fluorescent in the acidic conditions of late phagosomes and demonstrates resistance to fluorescence quenching in this environment.[63] A total of 50,000 RAW264.7 cells, which were primed by 200 U/mL of IFNγ (575306, BioLegend) and labeled with CFSE (Carboxyfluorescein Succinimidyl Ester; C34554, Thermo Fisher Scientific) one day before the assay, was cocultured with 100,000 apoptosis-induced cells for 2 h in conditioning medium containing PStRAP- or mock-supernatant. For the competitive assay, either 20 μg/mL of purified annexin V protein (640901, BioLegend) or 40 μg/mL of mouse RAP protein was added to the medium during co-culturing. After the residual apoptotic cells were removed, the phagocytes were analyzed using flow cytometry. The percentage of CypHer5E-positive RAW264.7 cells was considered the rate of phagocytosis.

In vivo tumor challenges and treatments

Seven- to nine-week-old female mice were subcutaneously transplanted into the right flank with the desired number of tumor cells (MCA205 and its sublines: 5×10^5 cells; MC38: 3×10^5 cells) suspended in 100 μL of HBSS on day 0. Tumor volumes were calculated as (long diameter × square short diameter) × π/6, which were measured using calipers every other day until reaching 10% of the body weight. All animal studies are indicated in figures as individual values (dotted lines) and means (bold lines). To assess the therapeutic effect of PStRAP, MCA205-bearing mice on day 7 were treated with LNP/mRNA (1 mg/kg) or empty LNP as a control intravenously 6 h prior to intraperitoneally administering 10 mg/kg of L-OHP on the same day.

When the studies aimed at evaluating the induction of adaptive immune responses, tumor cells were precultured with 500 μM of MMC in complete DMEM for 3 h, and then the left flank of the mice was subcutaneously inoculated with the dying tumor cells (5×10^5 cells) suspended in 100 μL of PBS. To evaluate whether cell death induced by the endogenous CTL response induces the adaptive immune response, eight-week-old male OT-I mice were subcutaneously transplanted into the left flank with the viable OVA-expressing tumor cells (5×10^5 cells) suspended in 100 μL of HBSS. Viable parental cells were subcutaneously transplanted into the right flank seven days later.

In vitro restimulation with neoepitopes

The splenocytes were harvested from the tumor-bearing mice 26 days after MC38 transplantation (Figure 2C) and seeded in 48-well plate (3338, Corning Inc.) at 1 million cells per well in complete RPMI. The following three known neoepitope peptides of MC38 were synthesized by GeneScript: H-2D^b-restricted ADPGK (ADP-dependent glucokinase); ASMTN***M***ELM, H-2D^b-restricted REPS1 (RalBP1-associated Eps domain-containing protein 1); AQL***A***NDVVL, H-2K^b-restricted RPL18 (Ribosomal Protein L18); KILTFD***R***L.[20,21] The splenocytes cultured with 10 μM of individual peptide or no peptide for three days, the culture supernatants were then collected, and the amounts of IFNγ and TNFα were measured using ELISA.

Ex vivo cross-presentation

MMC-pulsed parental MCA205, MCA205-OVA, or MCA205-OVA-PStRAP cells (2×10^6 cells) were subcutaneously inoculated into the right flank. The tumor-draining subiliac lymph nodes were harvested one day later, and the APCs were prepared by removing T-, B-, and NK cells.[7] The effector CTLs were prepared from the spleens of the OT-I mice using an EasySep Mouse CD8a Positive Selection Kit II (#18953, STEMCELL Technologies, Vancouver, Canada). A total of 1 million effector cells were cocultured with 250,000 APCs in complete RPMI on a 24-well plate (3337, Corning Inc.) for three days. The amounts of IFNγ in the culture supernatants were measured using ELISA.

In vivo T-cell proliferation assay

CD8$^+$ T-cells were enriched by EasySep Mouse CD8a Positive Selection Kit II (18953, STEMCELL Technologies, Vancouver, Canada) from splenocytes of OT-I mice and were labeled with CFSE. CFSE-labeled OT-I cells were adoptive transferred into wild-type C57BL/6NJ mice via tail vein injection (1.2×10^6 cells per mouse). One day later, recipient mice were subcutaneously inoculated with MMC-pulsed MCA205-OVA, MCA205-OVA-PStRAP, or MCA205-OVA-PStRAP-RAP cells (5×10^5 cells) into the right flank. The tumor-draining subiliac lymph node was harvested three days later, and the lymphocyte were stained with BV421 anti-mouse CD8a Antibody (clone 53-6.7; 100753, BioLegend) and PE anti-mouse TCR Vβ5.1, 5.2 Antibody (clone MR9-4; 139504, BioLegend) for distinguishing the donor cells. The stained cells were analyzed using flow cytometry.

Sandwich ELISA

The amounts of mouse IFNγ and TNFα in the culture supernatants were detected using Mouse IFN-gamma DuoSet ELISA and Mouse TNF-alpha DuoSet ELISA (DY485 and DY410, respectively, R&D systems) according to the instructions provided by the manufacturer.

Cytokine array

MCA205-PstRAP or MCA205-empty cells (3×10^6 cells) were cultured in a φ60 dish (VTC-D60, AS ONE, Osaka, Japan). In the event of cell death induction, these cells were precultured with 500 μM of MMC. 3 h later, the cells were washed three times, and the medium was exchanged. After cultivation for 24 h, culture supernatants were collected. To detect 111 mouse cytokines, chemokines, and growth factors, 1 mL of culture supernatants was loaded onto membrane of Proteome Profiler Mouse XL Cytokine Array

(ARY208, R&D Systems) and processed according to the manufacturer's protocol. Densitometric analysis was performed using the Protein Array Analyzer plugin (https://rsb.info.nih.gov/ij/macros/toolsets/Protein Array Analyzer.txt) on ImageJ.[64]

Quantification of luciferase bioluminescence intensity

To validate the delivery of the LNPs, Fluc mRNA-encapsulated LNPs were used. Seven-week-old mice were intravenously injected with these LNPs, and the desired organs were harvested 6 and 24 h later, accompanying an intravenously injected D-Luciferin (potassium salt) (14681, Cayman Chemical, Ann Arbor, MI) immediately before. The luminescence from the harvested organs *ex vivo* was measured using an IVIS Imaging System (PerkinElmer, Waltham, MA).

Detecting protein in serum

Blood was collected from the superficial temporal vein of the mice at the desired time points using Goldenrod Animal Lancets 4 mm (18310300, MEDIpoint, Long Island, NY). Blood samples were allowed to stand for 1 h followed by centrifugation (2,000 $\times$ g, 10 min), and then the resulting serum was collected. An AlphaScreen FLAG Detection Kit (6760613, Revvity, Waltham, MA) was used to detect DYKDDDK-tagged protein in the serum. The ALT and AST levels were measured using a Transaminase CII-test (431–30901, FUJIFILM Wako). Each kit was performed according to the instructions provided by the manufacturer.

Statistical analysis

All statistical analyses were performed using the GraphPad Prism 8 (GraphPad Software, San Diego, CA). For comparison of the means between groups, an unpaired Welch's t test was applied. Comparisons of the means among two or more groups were performed using one- or two-way analysis of variance (ANOVA) followed by post hoc analysis with Tukey's test in some cases. Data are presented as the mean ± standard deviation (SD). The p values <.05 were considered statistically significant.

DATA AND CODE AVAILABILITY

All data supporting the findings of the study are available in the article and its supplemental information. All raw data are available from Y.M. upon reasonable request.

ACKNOWLEDGMENTS

We would like to thank Mr. Takeshi Ueda (Nitto Denko Corporation) for supporting us throughout this study. We thank Drs. Takashi Akazawa and Tomoya Ekawa (Osaka International Cancer Institute) for fruitful discussions as well as Mr. Hiroya Komaki and Mses. Yachiyo Kumamoto, Arisa Tsuda, Kazuko Shizuma, and Mitsuki Yamaguchi (Osaka International Cancer Institute) for supporting the experiments. We thank Dr. William R. Heath for providing the OT-I mice and Dr. Steven. A. Rosenberg for providing the murine cell lines. We would like to thank Editage (www.editage.jp) for the English language editing. This study was funded by the JSPS KAKENHI under grant number JP22K07268 (Y.M.).

AUTHOR CONTRIBUTIONS

Y.M.: conceptualization, data curation, formal analysis, funding acquisition, investigation, project administration, resources, visualization, writing – original draft, writing – review and editing. H.N.: data curation, investigation, resources, validation, visualization, writing – original draft, writing – review and editing. K.H.: resources, writing – review and editing. C.O.: resources, writing – review and editing. S.A.: writing – review and editing. K.M.: writing – review and editing. H.T.: conceptualization, funding acquisition, supervision, writing – original draft, writing – review and editing.

DECLARATION OF INTERESTS

Y.M. and H.T. are the inventors of patents application (PCT/JP2024/041545 and JP2023-199462) associated with this publication. H.N., K.H., C.O., S.A., and K.M. were employees of the Nitto Denko Corporation (Osaka, Japan) at the time of this study.

SUPPLEMENTAL INFORMATION

Supplemental information can be found online at https://doi.org/10.1016/j.ymthe.2026.05.013.

REFERENCES

1. Caspi, R.R. (2008). Immunotherapy of autoimmunity and cancer: the penalty for success. Nat. Rev. Immunol. *8*, 970–976. https://doi.org/10.1038/nri2438.

2. Allen, J.E., and Rückerl, D. (2017). The Silent Undertakers: Macrophages Programmed for Efferocytosis. Immunity *47*, 810–812. https://doi.org/10.1016/j.immuni.2017.10.010.

3. Fadok, V.A., Voelker, D.R., Campbell, P.A., Cohen, J.J., Bratton, D.L., and Henson, P.M. (1992). Exposure of phosphatidylserine on the surface of apoptotic lymphocytes triggers specific recognition and removal by macrophages. J. Immunol. *148*, 2207–2216.

4. Birge, R.B., Boeltz, S., Kumar, S., Carlson, J., Wanderley, J., Calianese, D., Barcinski, M., Brekken, R.A., Huang, X., Hutchins, J.T., et al. (2016). Phosphatidylserine is a global immunosuppressive signal in efferocytosis, infectious disease, and cancer. Cell Death Differ. *23*, 962–978. https://doi.org/10.1038/cdd.2016.11.

5. Hanayama, R., Tanaka, M., Miwa, K., Shinohara, A., Iwamatsu, A., and Nagata, S. (2002). Identification of a factor that links apoptotic cells to phagocytes. Nature *417*, 182–187. https://doi.org/10.1038/417182a.

6. Asano, K., Miwa, M., Miwa, K., Hanayama, R., Nagase, H., Nagata, S., and Tanaka, M. (2004). Masking of phosphatidylserine inhibits apoptotic cell engulfment and induces autoantibody production in mice. J. Exp. Med. *200*, 459–467. https://doi.org/10.1084/jem.20040342.

7. Mizote, Y., Inoue, T., Akazawa, T., Kunimasa, K., Tamiya, M., Kumamoto, Y., Tsuda, A., Yoshida, S., Tatsumi, K., Ekawa, T., et al. (2024). Potent CTLs can be induced against tumor cells in an environment of lower levels of systemic MFG-E8. Cancer Sci. *115*, 1114–1128. https://doi.org/10.1111/cas.16099.

8. Jinushi, M., Sato, M., Kanamoto, A., Itoh, A., Nagai, S., Koyasu, S., Dranoff, G., and Tahara, H. (2009). Milk fat globule epidermal growth factor-8 blockade triggers tumor destruction through coordinated cell-autonomous and immune-mediated mechanisms. J. Exp. Med. *206*, 1317–1326. https://doi.org/10.1084/jem.20082614.

9. Zhou, Y.J., and Binder, R.J. (2014). The heat shock protein-CD91 pathway mediates tumor immunosurveillance. Oncoimmunology *3*, e28222. https://doi.org/10.4161/onci.28222.

10. Binder, R.J., Han, D.K., and Srivastava, P.K. (2000). CD91: a receptor for heat shock protein gp96. Nat. Immunol. *1*, 151–155. https://doi.org/10.1038/77835.

11. Basu, S., Binder, R.J., Ramalingam, T., and Srivastava, P.K. (2001). CD91 is a common receptor for heat shock proteins gp96, hsp90, hsp70, and calreticulin. Immunity *14*, 303–313. https://doi.org/10.1016/s1074-7613(01)00111-x.

12. Gardai, S.J., McPhillips, K.A., Frasch, S.C., Janssen, W.J., Starefeldt, A., Murphy-Ullrich, J.E., Bratton, D.L., Oldenborg, P.A., Michalak, M., and Henson, P.M. (2005). Cell-surface calreticulin initiates clearance of viable or apoptotic cells through trans-activation of LRP on the phagocyte. Cell *123*, 321–334. https://doi.org/10.1016/j.cell.2005.08.032.

13. Obeid, M., Tesniere, A., Ghiringhelli, F., Fimia, G.M., Apetoh, L., Perfettini, J.L., Castedo, M., Mignot, G., Panaretakis, T., Casares, N., et al. (2007). Calreticulin exposure dictates the immunogenicity of cancer cell death. Nat. Med. *13*, 54–61. https://doi.org/10.1038/nm1523.

14. Galvez-Cancino, F., Simpson, A.P., Costoya, C., Matos, I., Qian, D., Peggs, K.S., Litchfield, K., and Quezada, S.A. (2024). Fcgamma receptors and

immunomodulatory antibodies in cancer. Nat. Rev. Cancer *24*, 51–71. https://doi.org/10.1038/s41568-023-00637-8.

15. Yeung, T., Gilbert, G.E., Shi, J., Silvius, J., Kapus, A., and Grinstein, S. (2008). Membrane phosphatidylserine regulates surface charge and protein localization. Science *319*, 210–213. https://doi.org/10.1126/science.1152066.
16. Leventis, P.A., and Grinstein, S. (2010). The distribution and function of phosphatidylserine in cellular membranes. Annu. Rev. Biophys. *39*, 407–427. https://doi.org/10.1146/annurev.biophys.093008.131234.
17. Bu, G., Geuze, H.J., Strous, G.J., and Schwartz, A.L. (1995). 39 kDa receptor-associated protein is an ER resident protein and molecular chaperone for LDL receptor-related protein. EMBO J. *14*, 2269–2280. https://doi.org/10.1002/j.1460-2075.1995.tb07221.x.
18. Migliorini, M.M., Behre, E.H., Brew, S., Ingham, K.C., and Strickland, D.K. (2003). Allosteric modulation of ligand binding to low density lipoprotein receptor-related protein by the receptor-associated protein requires critical lysine residues within its carboxyl-terminal domain. J. Biol. Chem. *278*, 17986–17992. https://doi.org/10.1074/jbc.M212592200.
19. Pawaria, S., and Binder, R.J. (2011). CD91-dependent programming of T-helper cell responses following heat shock protein immunization. Nat. Commun. *2*, 521. https://doi.org/10.1038/ncomms1524.
20. Yadav, M., Jhunjhunwala, S., Phung, Q.T., Lupardus, P., Tanguay, J., Bumbaca, S., Franci, C., Cheung, T.K., Fritsche, J., Weinschenk, T., et al. (2014). Predicting immunogenic tumour mutations by combining mass spectrometry and exome sequencing. Nature *515*, 572–576. https://doi.org/10.1038/nature14001.
21. Hos, B.J., Camps, M.G.M., Bulk, J.v.d., Tondini, E., Ende, T.C.v.d., Ruano, D., Franken, K., Janssen, G.M.C., Ru, A., Filippov, D.V., et al. (2019). Identification of a neo-epitope dominating endogenous CD8 T cell responses to MC-38 colorectal cancer. Oncoimmunology *9*, 1673125. https://doi.org/10.1080/2162402X.2019.1673125.
22. Kurts, C., Heath, W.R., Carbone, F.R., Allison, J., Miller, J.F., and Kosaka, H. (1996). Constitutive class I-restricted exogenous presentation of self antigens in vivo. J. Exp. Med. *184*, 923–930. https://doi.org/10.1084/jem.184.3.923.
23. Wu, Y., Tibrewal, N., and Birge, R.B. (2006). Phosphatidylserine recognition by phagocytes: a view to a kill. Trends Cell Biol. *16*, 189–197. https://doi.org/10.1016/j.tcb.2006.02.003.
24. Wu, G., Ma, Z., Hu, W., Wang, D., Gong, B., Fan, C., Jiang, S., Li, T., Gao, J., and Yang, Y. (2017). Molecular insights of Gas6/TAM in cancer development and therapy. Cell Death Dis. *8*, e2700. https://doi.org/10.1038/cddis.2017.113.
25. Kobayashi, N., Karisola, P., Peña-Cruz, V., Dorfman, D.M., Jinushi, M., Umetsu, S.E., Butte, M.J., Nagumo, H., Chernova, I., Zhu, B., et al. (2007). TIM-1 and TIM-4 glycoproteins bind phosphatidylserine and mediate uptake of apoptotic cells. Immunity *27*, 927–940. https://doi.org/10.1016/j.immuni.2007.11.011.
26. Miyanishi, M., Tada, K., Koike, M., Uchiyama, Y., Kitamura, T., and Nagata, S. (2007). Identification of Tim4 as a phosphatidylserine receptor. Nature *450*, 435–439. https://doi.org/10.1038/nature06307.
27. Miyanishi, M., Segawa, K., and Nagata, S. (2012). Synergistic effect of Tim4 and MFG-E8 null mutations on the development of autoimmunity. Int. Immunol. *24*, 551–559. https://doi.org/10.1093/intimm/dxs064.
28. Ran, S., He, J., Huang, X., Soares, M., Scothorn, D., and Thorpe, P.E. (2005). Antitumor effects of a monoclonal antibody that binds anionic phospholipids on the surface of tumor blood vessels in mice. Clin. Cancer Res. *11*, 1551–1562. https://doi.org/10.1158/1078-0432.CCR-04-1645.
29. Lauber, K., Blumenthal, S.G., Waibel, M., and Wesselborg, S. (2004). Clearance of apoptotic cells: getting rid of the corpses. Mol. Cell *14*, 277–287. https://doi.org/10.1016/s1097-2765(04)00237-0.
30. Yan, X., Doffek, K., Yin, C., Krein, M., Phillips, M., Sugg, S.L., Johnson, B., and Shilyansky, J. (2012). Annexin-V promotes anti-tumor immunity and inhibits neuroblastoma growth in vivo. Cancer Immunol. Immunother. *61*, 1917–1927. https://doi.org/10.1007/s00262-012-1250-4.
31. Dasgupta, S.K., Guchhait, P., and Thiagarajan, P. (2006). Lactadherin binding and phosphatidylserine expression on cell surface-comparison with annexin A5. Transl. Res. *148*, 19–25. https://doi.org/10.1016/j.lab.2006.03.006.
32. Shi, J., Shi, Y., Waehrens, L.N., Rasmussen, J.T., Heegaard, C.W., and Gilbert, G.E. (2006). Lactadherin detects early phosphatidylserine exposure on immortalized leukemia cells undergoing programmed cell death. Cytometry A *69A*, 1193–1201. https://doi.org/10.1002/cyto.a.20345.
33. Zhou, Y.J., Messmer, M.N., and Binder, R.J. (2014). Establishment of tumor-associated immunity requires interaction of heat shock proteins with CD91. Cancer Immunol. Res. *2*, 217–228. https://doi.org/10.1158/2326-6066.CIR-13-0132.
34. Mark, M.R., Chen, J., Hammonds, R.G., Sadick, M., and Godowsk, P.J. (1996). Characterization of Gas6, a member of the superfamily of G domain-containing proteins, as a ligand for Rse and Axl. J. Biol. Chem. *271*, 9785–9789. https://doi.org/10.1074/jbc.271.16.9785.
35. Lu, Q., and Lemke, G. (2001). Homeostatic regulation of the immune system by receptor tyrosine kinases of the Tyro 3 family. Science *293*, 306–311. https://doi.org/10.1126/science.1061663.
36. Peeters, M.J.W., Rahbech, A., and Thor Straten, P. (2020). TAM-ing T cells in the tumor microenvironment: implications for TAM receptor targeting. Cancer Immunol. Immunother. *69*, 237–244. https://doi.org/10.1007/s00262-019-02421-w.
37. Flannagan, R.S., Canton, J., Furuya, W., Glogauer, M., and Grinstein, S. (2014). The phosphatidylserine receptor TIM4 utilizes integrins as coreceptors to effect phagocytosis. Mol. Biol. Cell *25*, 1511–1522. https://doi.org/10.1091/mbc.E13-04-0212.
38. Zhang, Q., Wang, H., Wu, X., Liu, B., Liu, W., Wang, R., Liang, X., Ma, C., and Gao, L. (2015). TIM-4 promotes the growth of non-small-cell lung cancer in a RGD motif-dependent manner. Br. J. Cancer *113*, 1484–1492. https://doi.org/10.1038/bjc.2015.323.
39. Santiago, C., Ballesteros, A., Martínez-Muñoz, L., Mellado, M., Kaplan, G.G., Freeman, G.J., and Casasnovas, J.M. (2007). Structures of T cell immunoglobulin mucin protein 4 show a metal-Ion-dependent ligand binding site where phosphatidylserine binds. Immunity *27*, 941–951. https://doi.org/10.1016/j.immuni.2007.11.008.
40. Théry, C., Zitvogel, L., and Amigorena, S. (2002). Exosomes: composition, biogenesis and function. Nat. Rev. Immunol. *2*, 569–579. https://doi.org/10.1038/nri855.
41. Sharma, R., Huang, X., Brekken, R.A., and Schroit, A.J. (2017). Detection of phosphatidylserine-positive exosomes for the diagnosis of early-stage malignancies. Br. J. Cancer *117*, 545–552. https://doi.org/10.1038/bjc.2017.183.
42. Lindenbergh, M.F.S., and Stoorvogel, W. (2018). Antigen Presentation by Extracellular Vesicles from Professional Antigen-Presenting Cells. Annu. Rev. Immunol. *36*, 435–459. https://doi.org/10.1146/annurev-immunol-041015-055700.
43. Groß, R., Reßin, H., von Maltitz, P., Albers, D., Schneider, L., Bley, H., Hoffmann, M., Cortese, M., Gupta, D., Deniz, M., et al. (2024). Phosphatidylserine-exposing extracellular vesicles in body fluids are an innate defence against apoptotic mimicry viral pathogens. Nat. Microbiol. *9*, 905–921. https://doi.org/10.1038/s41564-024-01637-6.
44. Zitvogel, L., Regnault, A., Lozier, A., Wolfers, J., Flament, C., Tenza, D., Ricciardi-Castagnoli, P., Raposo, G., and Amigorena, S. (1998). Eradication of established murine tumors using a novel cell-free vaccine: dendritic cell-derived exosomes. Nat. Med. *4*, 594–600. https://doi.org/10.1038/nm0598-594.
45. Ma, F., Vayalil, J., Lee, G., Wang, Y., and Peng, G. (2021). Emerging role of tumor-derived extracellular vesicles in T cell suppression and dysfunction in the tumor microenvironment. J. Immunother. Cancer *9*, e003217. https://doi.org/10.1136/jitc-2021-003217.
46. Kuang, L., Wu, L., and Li, Y. (2025). Extracellular vesicles in tumor immunity: mechanisms and novel insights. Mol. Cancer *24*, 45. https://doi.org/10.1186/s12943-025-02233-w.
47. Greening, D.W., Xu, R., Rai, A., Suwakulsiri, W., Chen, M., and Simpson, R.J. (2025). Clinical relevance of extracellular vesicles in cancer - therapeutic and diagnostic potential. Nat. Rev. Clin. Oncol. *22*, 924–952. https://doi.org/10.1038/s41571-025-01074-2.
48. Herz, J., and Strickland, D.K. (2001). LRP: a multifunctional scavenger and signaling receptor. J. Clin. Invest. *108*, 779–784. https://doi.org/10.1172/JCI13992.
49. Lillis, A.P., Van Duyn, L.B., Murphy-Ullrich, J.E., and Strickland, D.K. (2008). LDL receptor-related protein 1: unique tissue-specific functions revealed by selective gene knockout studies. Physiol. Rev. *88*, 887–918. https://doi.org/10.1152/physrev.00033.2007.

50. Laatsch, A., Panteli, M., Sornsakrin, M., Hoffzimmer, B., Grewal, T., and Heeren, J. (2012). Low density lipoprotein receptor-related protein 1 dependent endosomal trapping and recycling of apolipoprotein E. PLoS One *7*, e29385. https://doi.org/10.1371/journal.pone.0029385.

51. Gordts, P.L.S.M., Bartelt, A., Nilsson, S.K., Annaert, W., Christoffersen, C., Nielsen, L.B., Heeren, J., and Roebroek, A.J.M. (2012). Impaired LDL receptor-related protein 1 translocation correlates with improved dyslipidemia and atherosclerosis in apoE-deficient mice. PLoS One *7*, e38330. https://doi.org/10.1371/journal.pone.0038330.

52. Garg, A.D., Romano, E., Rufo, N., and Agostinis, P. (2016). Immunogenic versus tolerogenic phagocytosis during anticancer therapy: mechanisms and clinical translation. Cell Death Differ. *23*, 938–951. https://doi.org/10.1038/cdd.2016.5.

53. Salimu, J., Spary, L.K., Al-Taei, S., Clayton, A., Mason, M.D., Staffurth, J., and Tabi, Z. (2015). Cross-Presentation of the Oncofetal Tumor Antigen 5T4 from Irradiated Prostate Cancer Cells–A Key Role for Heat-Shock Protein 70 and Receptor CD91. Cancer Immunol. Res. *3*, 678–688. https://doi.org/10.1158/2326-6066.CIR-14-0079.

54. Hart, J.P., Gunn, M.D., and Pizzo, S.V. (2004). A CD91-positive subset of CD11c+ blood dendritic cells: characterization of the APC that functions to enhance adaptive immune responses against CD91-targeted antigens. J. Immunol. *172*, 70–78. https://doi.org/10.4049/jimmunol.172.1.70.

55. Nayak, D.A., Sedlacek, A.L., Cillo, A.R., Watkins, S.C., and Binder, R.J. (2024). CD91 and Its Ligand gp96 Confer Cross-Priming Capabilities to Multiple APCs during Immune Responses to Nascent, Emerging Tumors. Cancer Immunol. Res. *12*, 1663–1676. https://doi.org/10.1158/2326-6066.CIR-24-0326.

56. Kroemer, G., Galluzzi, L., Kepp, O., and Zitvogel, L. (2013). Immunogenic cell death in cancer therapy. Annu. Rev. Immunol. *31*, 51–72. https://doi.org/10.1146/annurev-immunol-032712-100008.

57. Nagata, S., Suzuki, J., Segawa, K., and Fujii, T. (2016). Exposure of phosphatidylserine on the cell surface. Cell Death Differ. *23*, 952–961. https://doi.org/10.1038/cdd.2016.7.

58. Shlomovitz, I., Speir, M., and Gerlic, M. (2019). Flipping the dogma - phosphatidylserine in non-apoptotic cell death. Cell Commun. Signal. *17*, 139. https://doi.org/10.1186/s12964-019-0437-0.

59. Ooishi, T., Nadano, D., Matsuda, T., and Oshima, K. (2017). Extracellular vesicle-mediated MFG-E8 localization in the extracellular matrix is required for its integrin-dependent function in mouse mammary epithelial cells. Genes Cells. *22*, 885–899. https://doi.org/10.1111/gtc.12521.

60. Hashiba, K., Taguchi, M., Sakamoto, S., Otsu, A., Maeda, Y., Ebe, H., Okazaki, A., Harashima, H., and Sato, Y. (2024). Overcoming thermostability challenges in mRNA-lipid nanoparticle systems with piperidine-based ionizable lipids. Commun. Biol. *7*, 556. https://doi.org/10.1038/s42003-024-06235-0.

61. Hashiba, K., Taguchi, M., Sakamoto, S., Otsu, A., Maeda, Y., Suzuki, Y., Ebe, H., Okazaki, A., Harashima, H., and Sato, Y. (2024). Impact of Lipid Tail Length on the Organ Selectivity of mRNA-Lipid Nanoparticles. Nano Lett. *24*, 12758–12767. https://doi.org/10.1021/acs.nanolett.4c02566.

62. Thorn, C.R., Sharma, D., Combs, R., Bhujbal, S., Romine, J., Zheng, X., Sunasara, K., and Badkar, A. (2022). The journey of a lifetime - development of Pfizer's COVID-19 vaccine. Curr. Opin. Biotechnol. *78*, 102803. https://doi.org/10.1016/j.copbio.2022.102803.

63. Beletskii, A., Cooper, M., Sriraman, P., Chiriac, C., Zhao, L., Abbot, S., and Yu, L. (2005). High-throughput phagocytosis assay utilizing a pH-sensitive fluorescent dye. Biotechniques *39*, 894–897. https://doi.org/10.2144/000112001.

64. Schneider, C.A., Rasband, W.S., and Eliceiri, K.W. (2012). NIH Image to ImageJ: 25 years of image analysis. Nat. Methods *9*, 671–675. https://doi.org/10.1038/nmeth.2089.

YMTHE, Volume *34*

Supplemental Information

Restricting efferocytosis pathway to CD91 via phosphatidylserine-targeting chimeric protein augments antitumor immune responses

Yu Mizote, Hiroki Nishita, Kazuki Hashiba, Chisa Okuma, Sachiko Akane, Kenjiro Minomi, and Hideaki Tahara

A

Original 1 ATG CAG GTC TCC CGT GTG CTG GCC GCG CTG TGC GGC ATG CTA CTC TGC GCC TCT GGC CTC TTC GCC GCG TGT GAA ACC GGT TGT TCT ACA CAG CTG GGC ATG GAA GGG GGC GCC ATT GCT 120
Optimization ATG CAG GTC TCA CGC GTC CTC GCT GCC CTG TGT GGT ATG CTG CTA TGC GCA TCT GGG CTC TTT GCA GCC TGT GAA ACA GGA TGC TCC ACG CAA CTT GGG ATG GAA GGC GGC GCT ATC GCT
Amino acid 1 M Q V S R V L A A L C G M L L C A S G L F A A C E T G C S T Q L G M E G G A I A 40
MFG-E8 signal peptide

Original 121 GAT TCA CAG ATT TCC GCC TCG TCT GTG TAT ATG GGT TTC ATG GGC TTG CAG CGC TGG GGC CCG GAG CTG GCT CGT CTG TAC CGC ACA GGG ATC GTC AAT GCC TGG ACA GCC AGC AAC TAT 240
Optimization GAC TCG CAG ATC TCA GCC AGT TCG GTG TAC ATG GGG TTC ATG GGT CTA CAG AGA TGG GGA CCA GAG TTG GCG CGG CTA TAC CGT ACG GGA ATT GTG AAT GCA TGG ACA GCG AGC AAC TAT
Amino acid 41 D S Q I S A S S V Y M G F M G L Q R W G P E L A R L Y R T G I V N A W T A S N Y 80

Original 241 GAT AGC AAG CCC TGG ATC CAG GTG AAC CTT CTG CGG AAG ATG CGG GTA TCA GGT GTG ATG ACG CAG GGT GCC AGC CGT GCC GGG AGG GCG GAG TAC CTG AAG ACC TTC AAG GTG GCT TAC 360
Optimization GAT TCA AAG CCC TGG ATC CAA GTG AAC CTT TTG CGG AAG ATG CGT GTC AGC GGT GTG ATG ACA CAG GGT GCT TCC AGG GCC GGA AGG GCA GAA TAC TTG AAA ACT TTC AAA GTC GCT TAC
Amino acid 81 D S K P W I Q V N L L R K M R V S G V M T Q G A S R A G R A E Y L K T F K V A Y 120
MFG-E8 C1

Original 361 AGC CTC GAC GGA CGC AAG TTT GAG TTC ATC CAG GAT GAA AGC GGT GGA GAC AAG GAG TTT TTG GGT AAC CTG GAC AAC AAC AGC CTG AAG GTT AAC ATG TTC AAC CCG ACT CTG GAG GCA 480
Optimization TCT CTC GAT GGC CGC AAG TTC GAG TTC ATC CAG GAT GAG AGT GGT GGA GAT AAG GAG TTC CTG GGA AAT TTA GAT AAC AAC TCC CTC AAA GTT AAC ATG TTT AAT CCT ACG CTC GAG GCA
Amino acid 121 S L D G R K F E F I Q D E S G G D K E F L G N L D N N S L K V N M F N P T L E A 160

Original 481 CAG TAC ATA AAG CTG TAC CCT GTT TCG TGC CAC CGC GGC TGC ACC CTC CGC TTC GAG CTC CTG GGC TGT GAG TTG CAC GGA TGT TCT GAG CCC CTG GGC CTG AAG AAT AAC ACA ATT CCT 600
Optimization CAG TAT ATC AAG CTG TAT CCC GTG AGC TGC CAC AGG GGC TGT ACA CTC AGA TTT GAA CTG GGT TGT GAG CTT CAT GGG TGC TCT GAG CCC CTG GGA CTG AAG AAT AAC ACC ATA CCT
Amino acid 161 Q Y I K L Y P V S C H R G C T L R F E L L G C E L H G C S E P L G L K N N T I P 200

Original 601 GAC AGC CAG ATG TCA GCC TCC AGC AGC TAC AAG ACA TGG AAC CTG CGT GCT TTT GGC TGG TAC CCC CAC TTG GGA AGG CTG GAT AAT CAG GGC AAG ATC AAT GCC TGG ACG GCT CAG AGC 720
Optimization GAC AGC CAG ATG TCT GCT TCC AGT TCC TAC AAG ACA TGG AAC TTG AGG GCT TTT GGC TGG TAC CCA CAT CTC GGG CGG CTT GAC AAC CAG GGC AAA ATT AAT GCC TGG ACA GCT CAA TCA
Amino acid 201 D S Q M S A S S S Y K T W N L R A F G W Y P H L G R L D N Q G K I N A W T A Q S 240

Original 721 AAC AGT GCC AAG GAA TGG CTG CAG GTT GAC CTG GGC ACT CAG AGG CAA GTG ACA GGA ATC ATC ACC CAG GGG GCC CGT GAC TTT GGC CAC ATC CAG TAT GTG GCG TCC TAC AAG GTA GCC 840
Optimization AAC AGC GCG AAG GAA TGG CTG CAA GTG GAC CTG GGC ACT CAG CGA CAA GTG ACC GGA ATT ATC ACC CAA GGT GCC AGA GAC TTC GGC CAC ATT CAG TAT GTC GCC TCC TAC AAG GTA GCG
Amino acid 241 N S A K E W GL Q V D L G T Q R Q V T G I I T Q G A R D F G H I Q Y V A S Y K V A 280
MFG-E8 C2

Original 841 CAC AGT GAT GAT GGT GTG CAG TGG ACT GTA TAT GAG GAG CAA GGA AGC AGC AAG GTC TTC CAG GGC AAC TTG GAC AAC AAC TCC CAC AAG AAG AAC ATC TTC GAG AAA CCC TTC ATG GCT 960
Optimization CAC TCT GAT GAC GGC GTT CAG TGG ACC GTG TAT GAG GAG CAG GGC AGC AGC AAG GTA TTC CAG GGC AAC CTG GAT AAT AAT AGT CAC AAG AAA AAC ATC TTT GAG AAG CCA TTT ATG GCC
Amino acid 281 H S D D G V Q W T V Y E E Q G S S K V F Q G N L D N N S H K K N I F E K P F M A 320

Original 961 CGC TAC GTG CGT GTC CTT CCA GTG TCC TGG CAT AAC CGC ATC ACC CTG CGC CTG GAG CTG CTG GGC TGT TAC TCG CGA GAG AAG AAC GAG CCG GAG ATG GCC GCC AAG CGC GAG TCC GGG 1080
Optimization CGC TAC GTC CGA GTG CTT CCT GTG TCC TGG CAT AAT AGA ATC ACT CTG CGG CTC GAA CTG CTG GGC TGC TAC TCT AGG GAA AAG AAC GAG CCC GAG ATG GCT GCC AAG CGG GAG TCG GGG
Amino acid 321 R Y V R V L P V S W H N R I T L R L E L L G C Y S R E K N E P E M A A K R E S G 360

Original 1081 GAG GAG TTC CGC ATG GAG AAG CTG AAC CAG CTA TGG GAG AAG GCC AAG CGG CTT CAT CTG TCT CCT GTG AGG CTG GCC GAG CTG CAT TCT GAC CTG AAG ATA CAA GAG AGG GAT GAA CTC 1200
Optimization GAG GAA TTT CGG ATG GAG AAG TTA AAT CAG TTA TGG GAA AAG GCT AAA AGA CTA CAT CTT TCT CCT GTT CGA CTG GCA GAG CTG CAC AGC GAT TTG AAA ATT CAG GAA CGT GAC GAG CTG
Amino acid 361 E E F R M E K L N Q L W E K A K R L H L S P V R L A E L H S D L K I Q E R D E L 400
RAP D1

Original 1201 AAC TGG AAA AAG CTG AAG GTG GAA GGC TTG GAT AAG GAT GGG GAG AAA GAA GCA AAA CTG ATC CAC AAC CTC AAC GTC ATC CTG GCC AGA TAC GGA CTG GAT GGG AGG AAG GAC GCC CAG 1320
Optimization AAC TGG AAA AAG TTA AAA GTG GAG GGT CTT GAC AAG GAT GGG GAA AAA GAG GCC AAG TTG ATT CAC AAC CTG AAT GTA ATC CTG GCA CGC TAT GGT CTG GAC GGA CGG AAA GAC GCC CAA
Amino acid 401 N W K K L K V E G L D K D G E K E A K L I H N L N V I L A R Y G L D G R K D A Q 440

BspQI site deletion
Original 1321 ATG GTG CAC AGC AAC GCC CTC AAT GAA GAC ACC CAG GAT GAG CTG GGG GAC CCC AGG CTG GAA AAG CTG TGG CAC AAG GCA AAG ACA TCA GGG AAA TTC TCC AGT GAG GAA CTG GAC AAG 1440
Optimization ATG GTA CAC AGC AAT GCC CTC AAT GAA GAC ACT CAA GAC GAA CTT GGA GAC CCG AGA TTA GAG AAG TTG TGG CAC AAA GCC AAA ACT AGT GGG AAG TTC AGT TCA GAG GAG CTG GAT AAA
Amino acid 441 M V H S N A L N E D T Q D E L G D P R L E K L W H K A K T S G K F S S E E L D K 480

Original 1441 CTG TGG AGA GAG TTT CTG CAT TAC AAA GAG AAG ATC CAG GAG TAC AAT GTG CTG CTA GAC ACA CTG AGC AGA GCT GAA GAA GGT TAT GAG AAC CTT CTC AGT CCC TCG GAC ATG GCC CAC 1560
Optimization CTG TGG AGA GAG TTC CTC CAT TAT AAA GAA AAA ATA CAG GAG TAC AAC GTT TTG TTA GAT ACC CTA AGC AGA GCT GAG GAG GGA TAT GAA AAT CTG CTG TCT CCA AGT GAC ATG GCA CAT
Amino acid 481 L W R E F L H Y K E K I Q E Y N V L L D T L S R A E E G Y E N L L S P S D M A H 520
RAP D2

Original 1561 ATC AAG AGC GAC ACC CTG ATC AGC AAG CAC AGT GAG CTG AAG GAC AGA CTG CGC AGT ATC AAC CAG GGC TTG GAC CGC CTG CGG AAG GTC AGC CAC CAG GGC TAT GGC TCC ACC ACT GAG 1680
Optimization ATA AAG TCC GAC ACC CTC ATA TCG AAA CAT TCA GAA CTT AAG GAC AGG CTC CGA TCA ATC AAC CAA GGC CTG GAT CGC CTC AGA AAA GTA AGC CAC CAG GGC TAC GGG TCC ACA ACT GAG
Amino acid 521 I K S D T L I S K H S E L K D R L R S I N Q G L D R L R K V S H Q G Y G S T T E 560

BspQI site deletion
Original 1681 TTT GAG GAA CCC CGG GTG ATA GAT CTG TGG GAC CTG GCT CAG TCT GCC AAC TTC ACT GAG AAG GAA CTG GAG TCA TTC AGG GAG GAG CTC AAG CAC TTT GAG GCC AAA ATT GAA AAG CAC 1800
Optimization TTT GAG GAA CCA CGT GTG ATT GAT CTC TGG GAC CTG GCC CAG AGC GCA AAC TTC ACC GAG AAG GAG CTG GAA TCT TTC CGA GAA GAA CTA AAG CAC TTT GAA GCC AAA ATC GAG AAA CAC
Amino acid 561 F E E P R V I D L W D L A Q S A N F T E K E L E S F R E E L K H F E A K I E K H 600
RAP D3

Original 1801 AAC CAC TAC CAG AAG CAG CTG GAG ATT TCC CAC CAG AAG CTG AAG CAC GTG GAG AGC ATC GGC GAC CCC GAG CAC ATC AGC CGC AAC AAG GAG AAA TAC GTG CTG CTG GAG GAG AAG ACC 1920
Optimization AAT CAC TAT CAG AAG CAG CTG GAA ATC AGC CAT CAG AAA CTG AAG CAT GTG GAG AGT ATT GGG GAT CCT GAG CAT ATA TCT AGG AAC AAG GAA AAG TAC GTG CTG CTC GAG GAG AAA ACC
Amino acid 601 N H Y Q K Q L E I S H Q K L K H V E S I G D P E H I S R N K E K Y V L L E E K T 640

Original 1921 AAG GAG CTG GGC TAC AAG GTG AAG AAG CAT CTA CAG GAC CTG TCT AGC AGG GTC TCA AGG GCT CGG GAC TAC AAG GAC GAC GAT GAC AAG TGA 2013
Optimization AAG GAG CTT GGG TAC AAG GTT AAA AAG CAC CTG CAG GAC CTA TCA TCC AGG GTC TCT CGG GCT CGC GAT TAT AAG GAC GAT GAC AAG TGA
Amino acid 641 K E L G Y K V K K H L Q D L S S R V S R A R D Y K D D D D K STOP
DYKDDDDK-tag

B

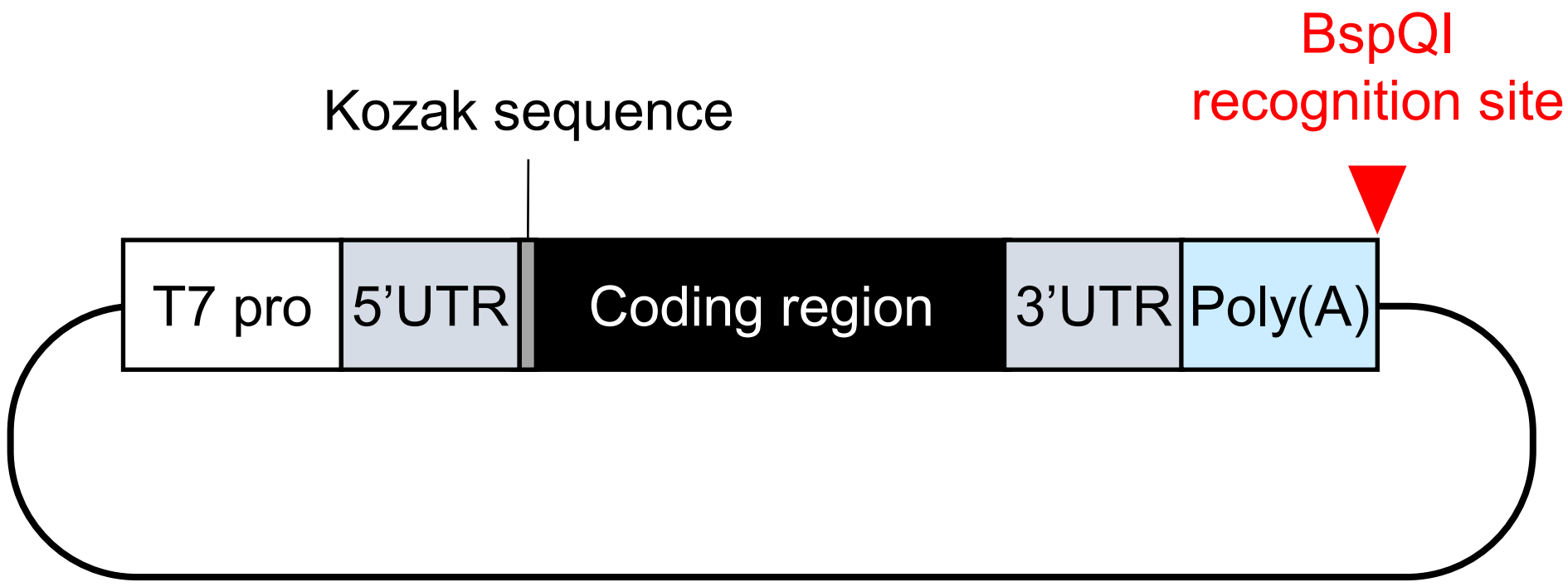

Figure S1. PStRAP sequence and DNA plasmid structure for synthesizing mRNA.

(A) The PStRAP original DNA, codon-optimized DNA, and amino acid sequences are depicted. The BspQI recognition site (GAAGAGC) was deleted from the spontaneous sequence to linearize the plasmid (green). The DNA replaced for optimizing the codon is shown red letters. Each amino acid (blue) was replaced with alanine in the PS binding activity deletion mutant (PStRAPΔPS). Each amino acid (purple) was replaced with alanine in the CD91 binding activity deletion mutant (PStRAPΔCD91). (B) The plasmid construct for synthesizing mRNA contains a T7 promoter, a 5'-untranslated region (UTR), a Kozak sequence, a coding region, a 3'-UTR, a 120 nt poly(A) tail, and a BspQI recognition site for linearization, in this order.

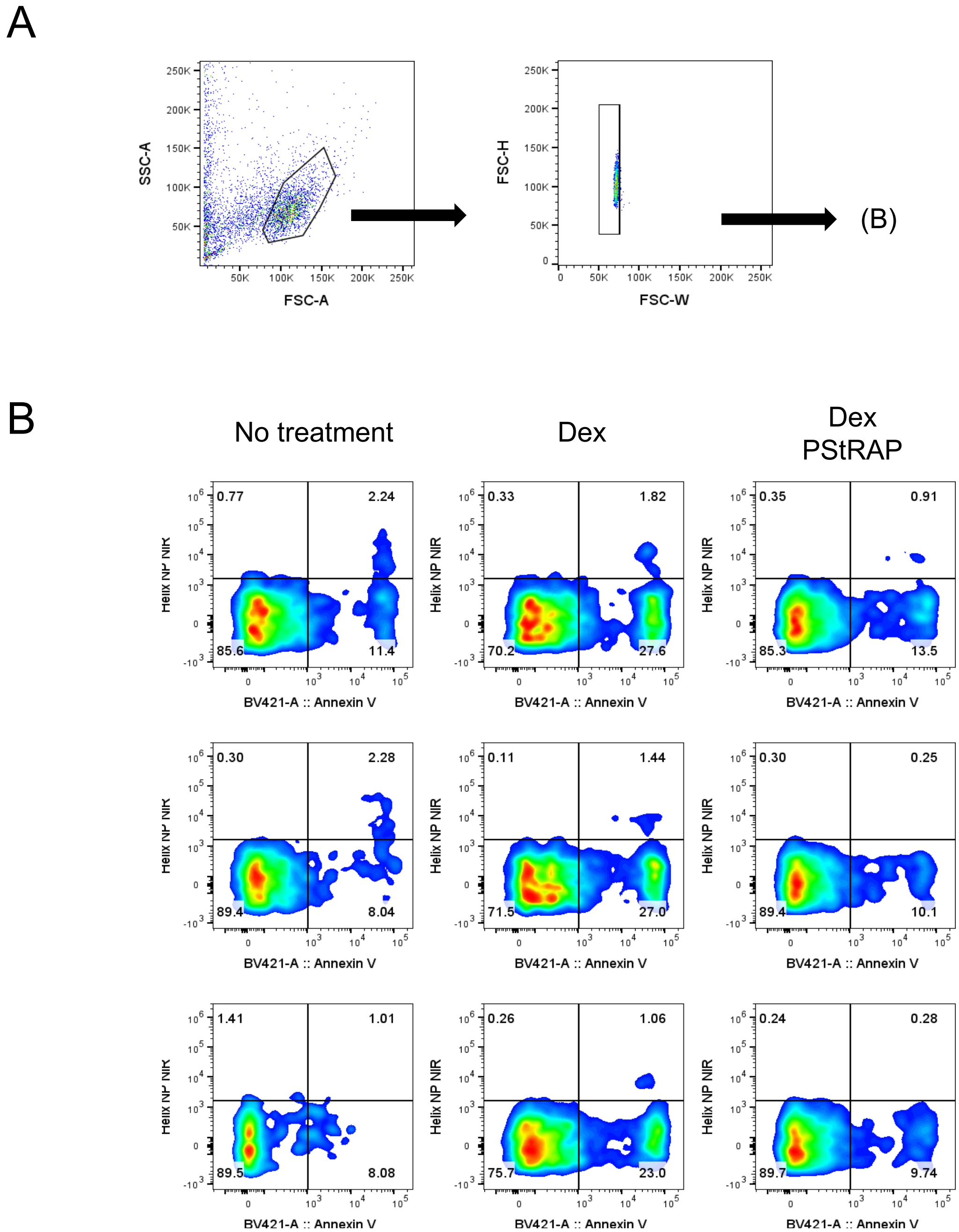

	No treatment	Dex	Dex PStRAP
Annexin V gMFI	197	650	225
	149	590	173
	154	454	73.1

Figure S2. Gating strategy of Annexin V staining and individual plots.
(A) The Annexin V$^+$ cells were defined by gating FSC/SSC and then single cell-populations, in that order. (B) Individual plots are depicted (*n* = 3/group). Individual geometric mean fluorescent intensity (gMFI) of annexin V is shown in a table. The relative gMFI is depicted in Figure 1E

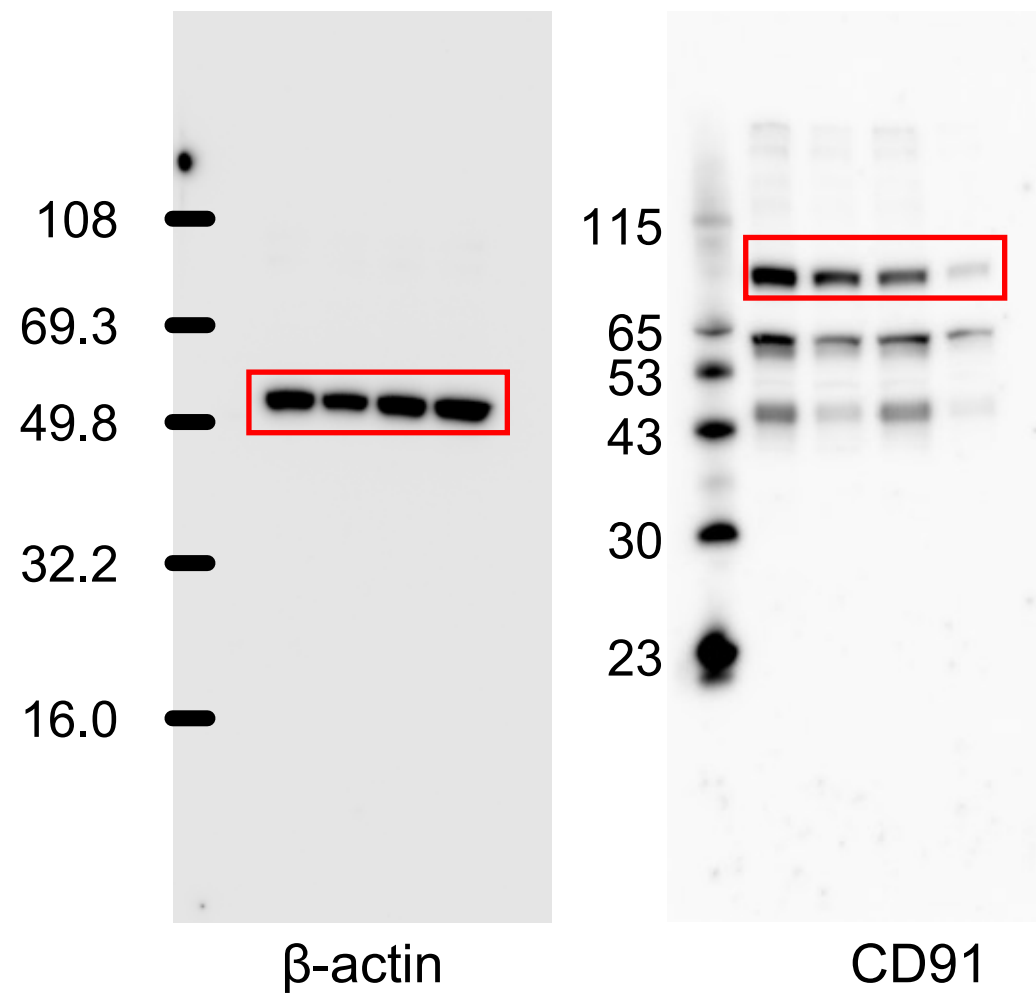

Figure S3. Expression levels of CD91 in RAW264.7 cell sublines. Original blot images depicting cropped regions for Figure 1F.

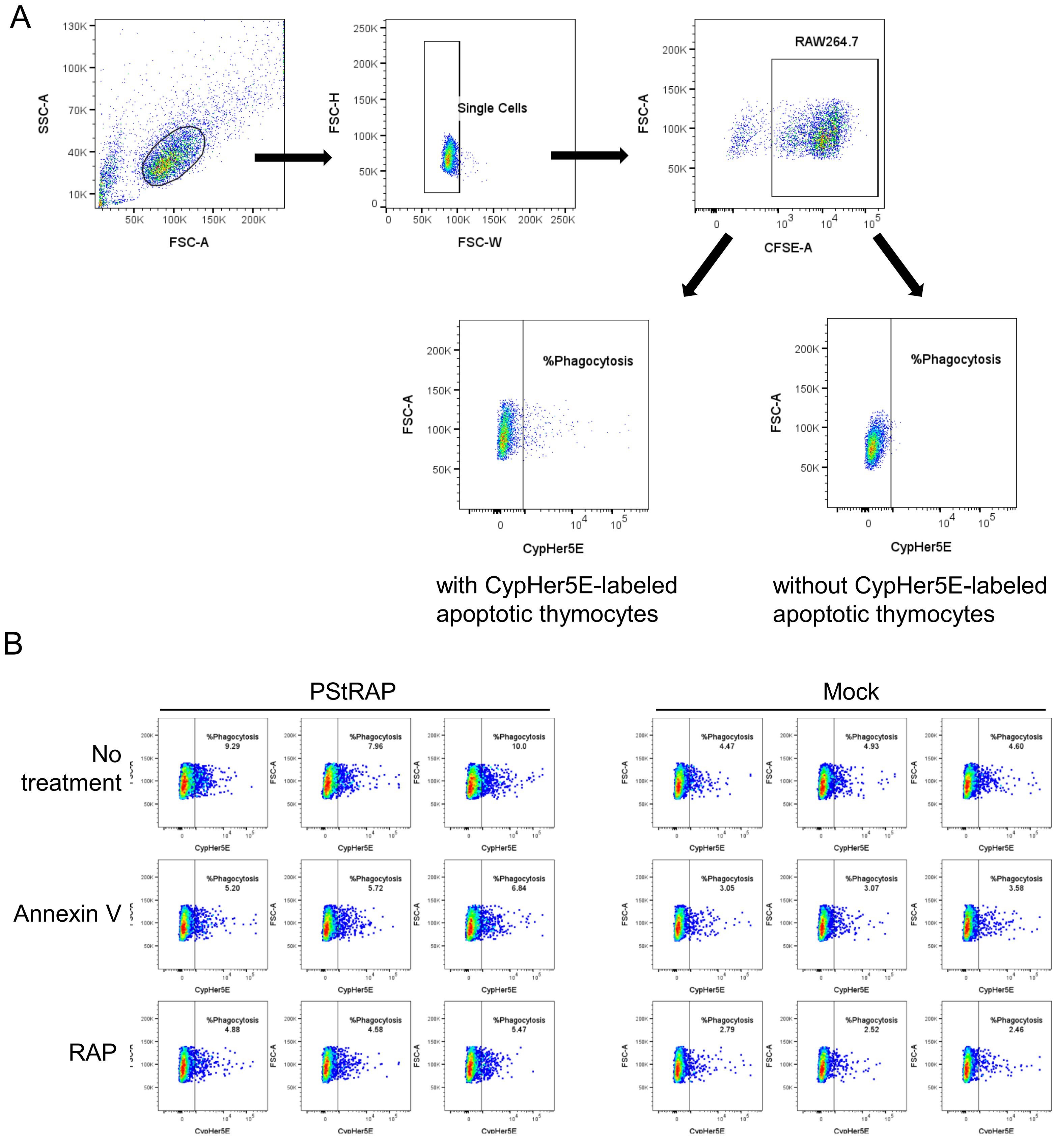

Figure S4. Gating strategy and phagocytosis assay.

(A) The CypHer5E$^+$ RAW264.7 cells were defined by gating single cell-populations and then CFSE$^+$, in that order. (B) The CypHer5E$^+$ cell rate is depicted as a percentage of phagocytosis in Figure 1H (n = 3/group).

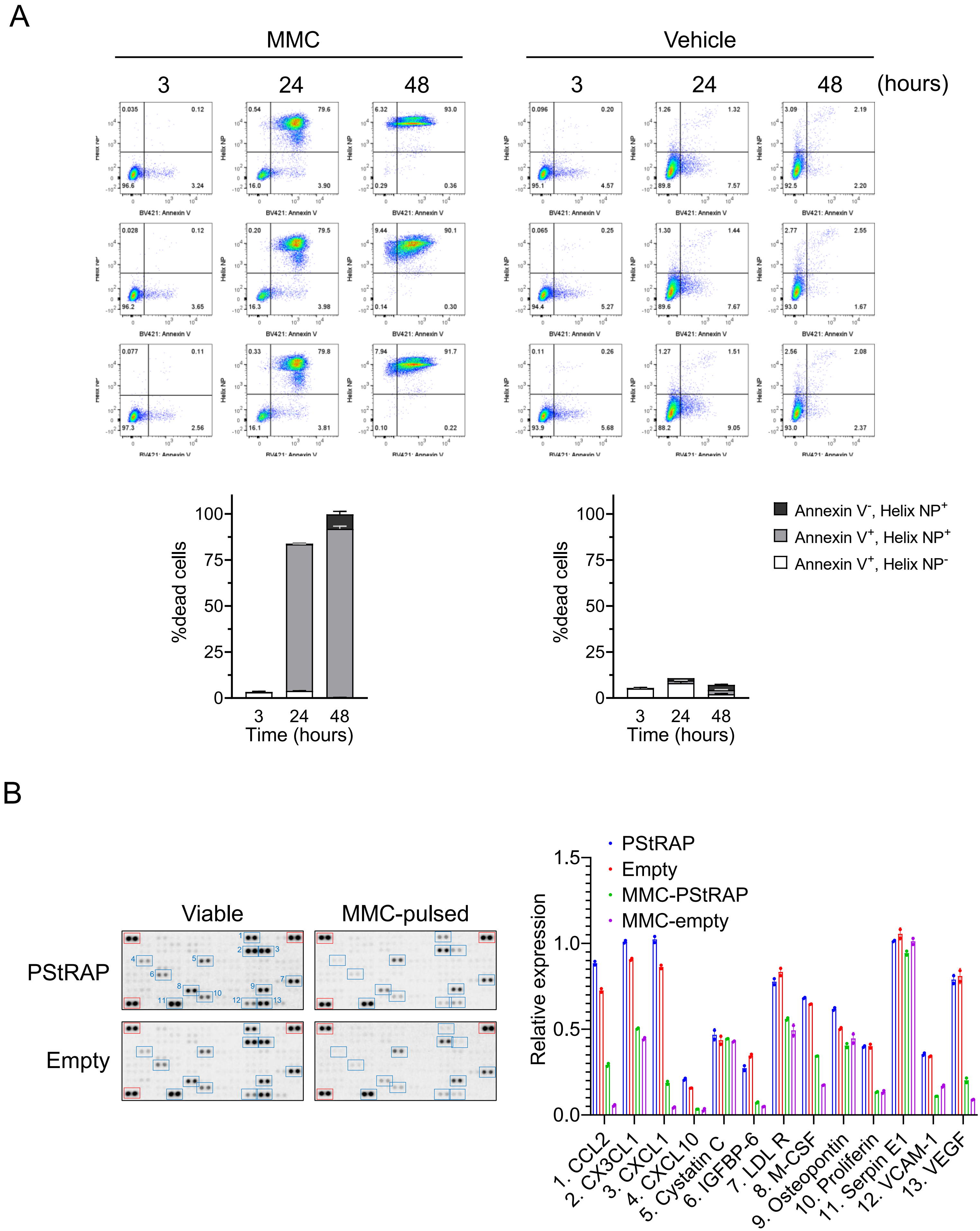
A
MMC
Vehicle
3
24
48
(hours)
BV421: Annexin V
Helix NP
%dead cells
100
75
50
25
0
3
24
48
Time (hours)
Annexin V-, Helix NP+
Annexin V+, Helix NP+
Annexin V+, Helix NP-
B
Viable
MMC-pulsed
PStRAP
Empty
PStRAP
Empty
MMC-PStRAP
MMC-empty
Relative expression
1.5
1.0
0.5
0.0
1. CCL2
2. CX3CL1
3. CXCL1
4. CXCL10
5. Cystatin C
6. IGFBP-6
7. LDL R
8. M-CSF
9. Osteopontin
10. Proliferin
11. Serpin E1
12. VCAM-1
13. VEGF

Figure S5. The characteristic of MMC-pulsed tumor cells.
(A) MMC (500 μM) or vehicle-pulsed MCA205 cells were co-stained with BV421-conjugated annexin V and Helix NP NIR at the desired time point (n = 3/group). Error bars show mean (SD). (B) The secreted factors in culture supernatant were detected using a membrane array. The culture supernatant was collected from viable or MMC-pulsed MCA205 transfectants (PStRAP or Empty), after culturing for 24 hours. The red boxes indicate the reference spots, while the blue box with a number indicates spots detected in the supernatant of viable cells (left). The density of the spots relative to the average value of the reference spots indicates the relative expression level of the secreted factor (right).

A

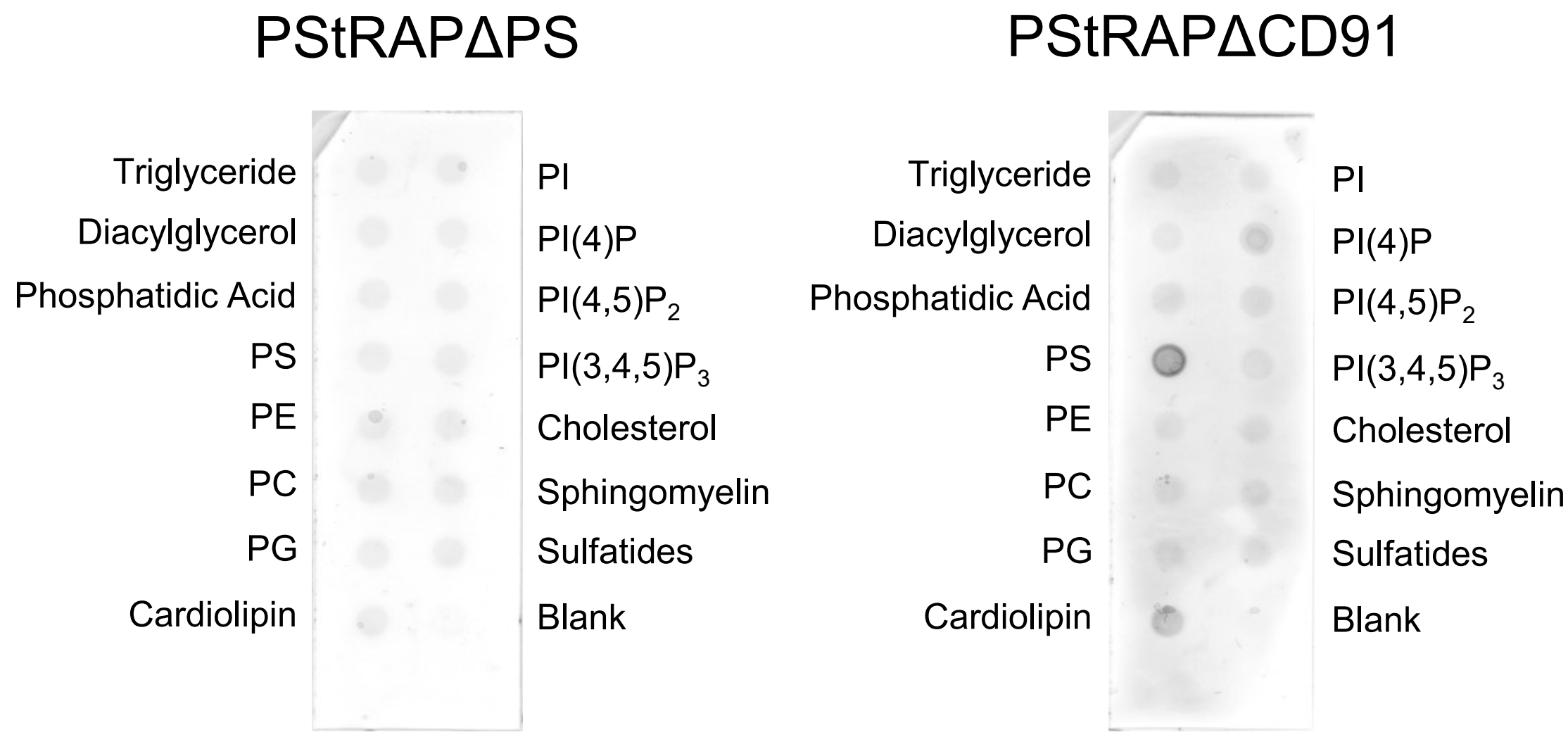

B

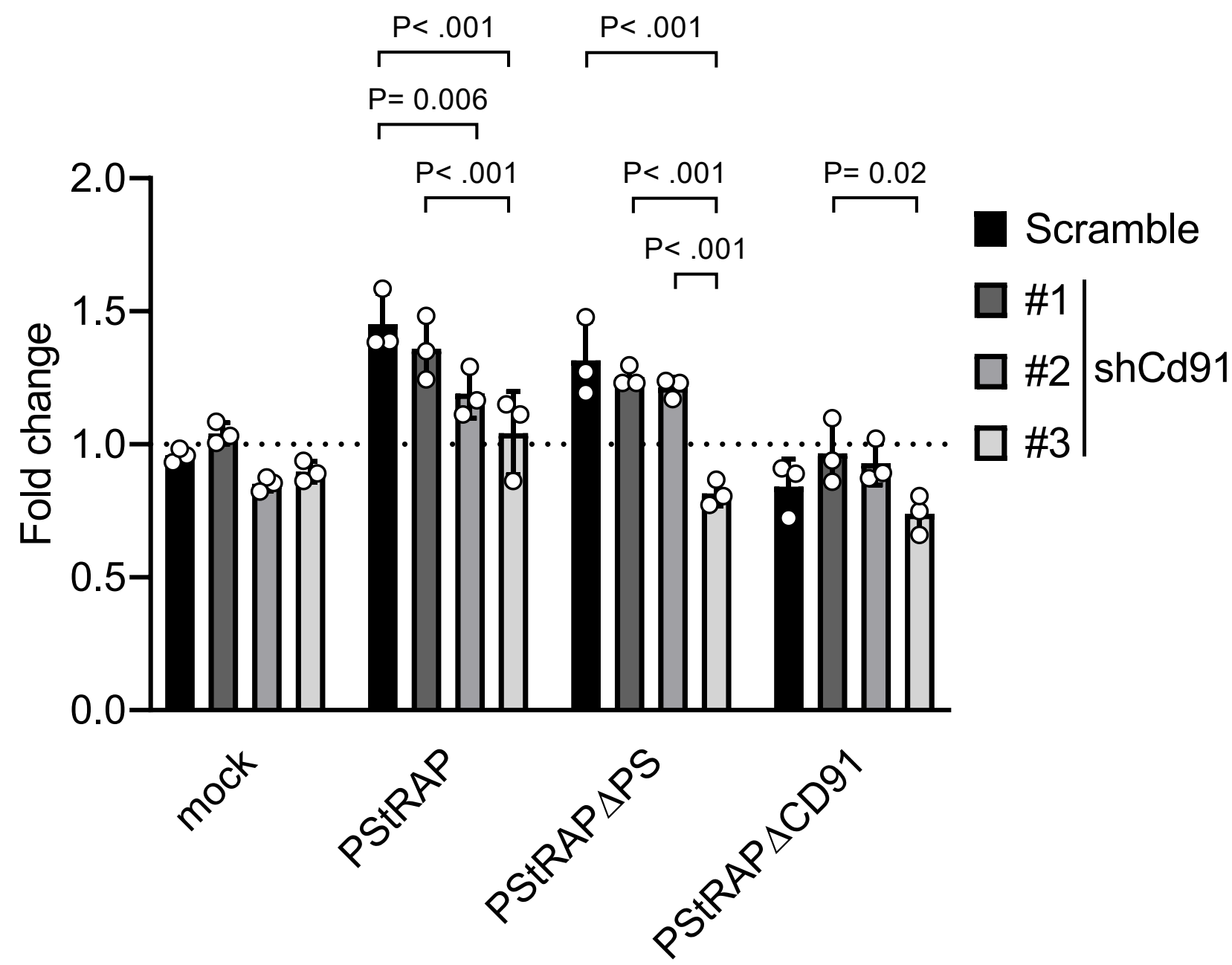

Figure S6. Loss-of-function mutants of PStRAP.

(A) A membrane lipid array was performed with the culture supernatants from MCA205 transfectants after two days of culturing. (B) The luciferase secreted upon NF-κB activation was measured in the culture supernatants from the RAW264.7 cell sublines (Figure 1E) one hour after stimulation with the culture supernatants of the transfectants (n = 3/group). The fold change was calculated using the luciferase level in the culture supernatants without stimulation as a reference. Error bars show mean (SD). Significance differences were determined using two-way ANOVA followed by post hoc Tukey's test.

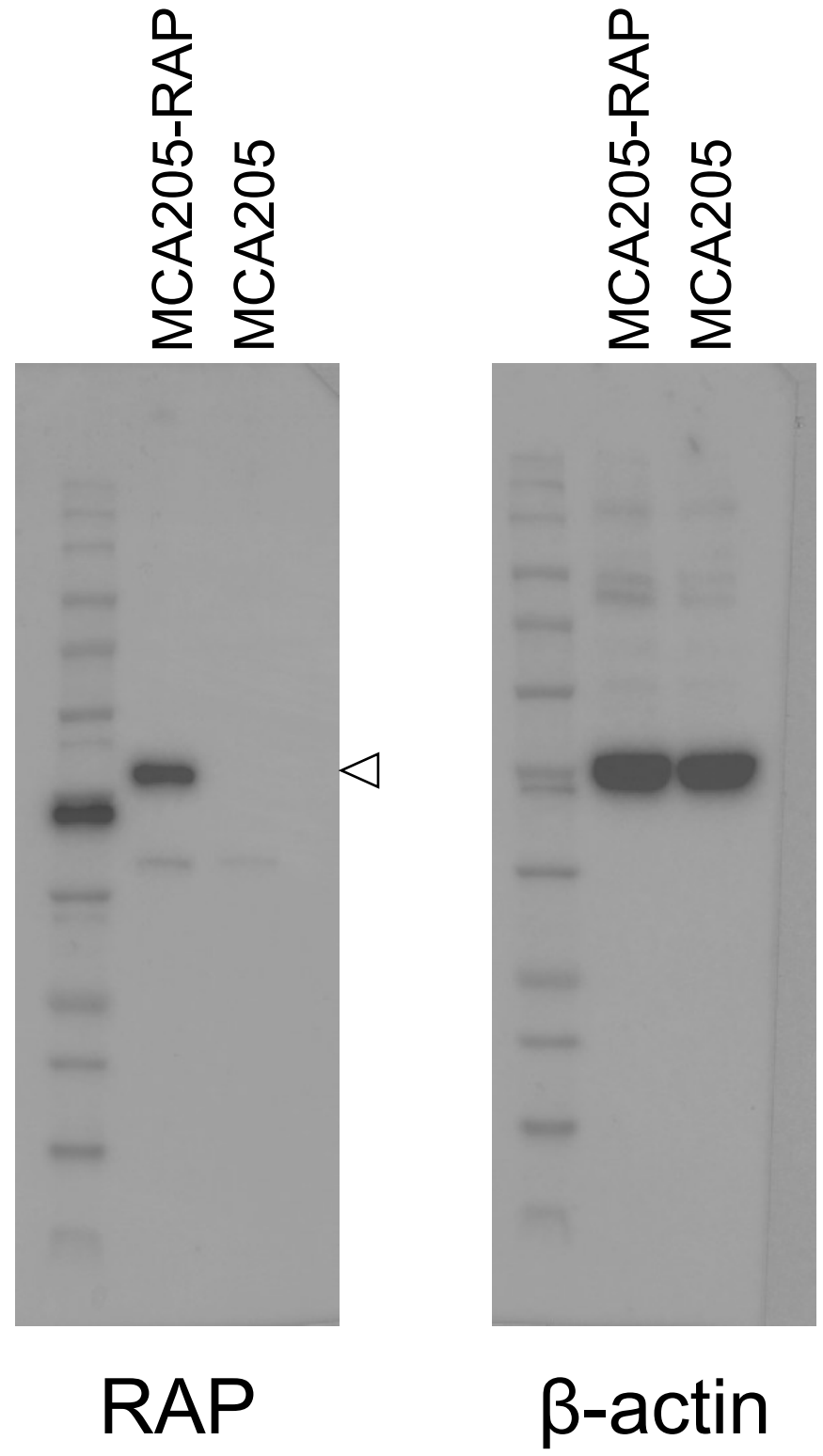

Figure S7. Expression of RAP in transfectant.
RAP expression in MCA205 transfectant used in Figures 3B and 3C was detected by immunoblotting. The RAP protein, which was tagged with PA at the C-terminus for differentiate its endogenous expression, was detected using an anti-PA tag antibody.

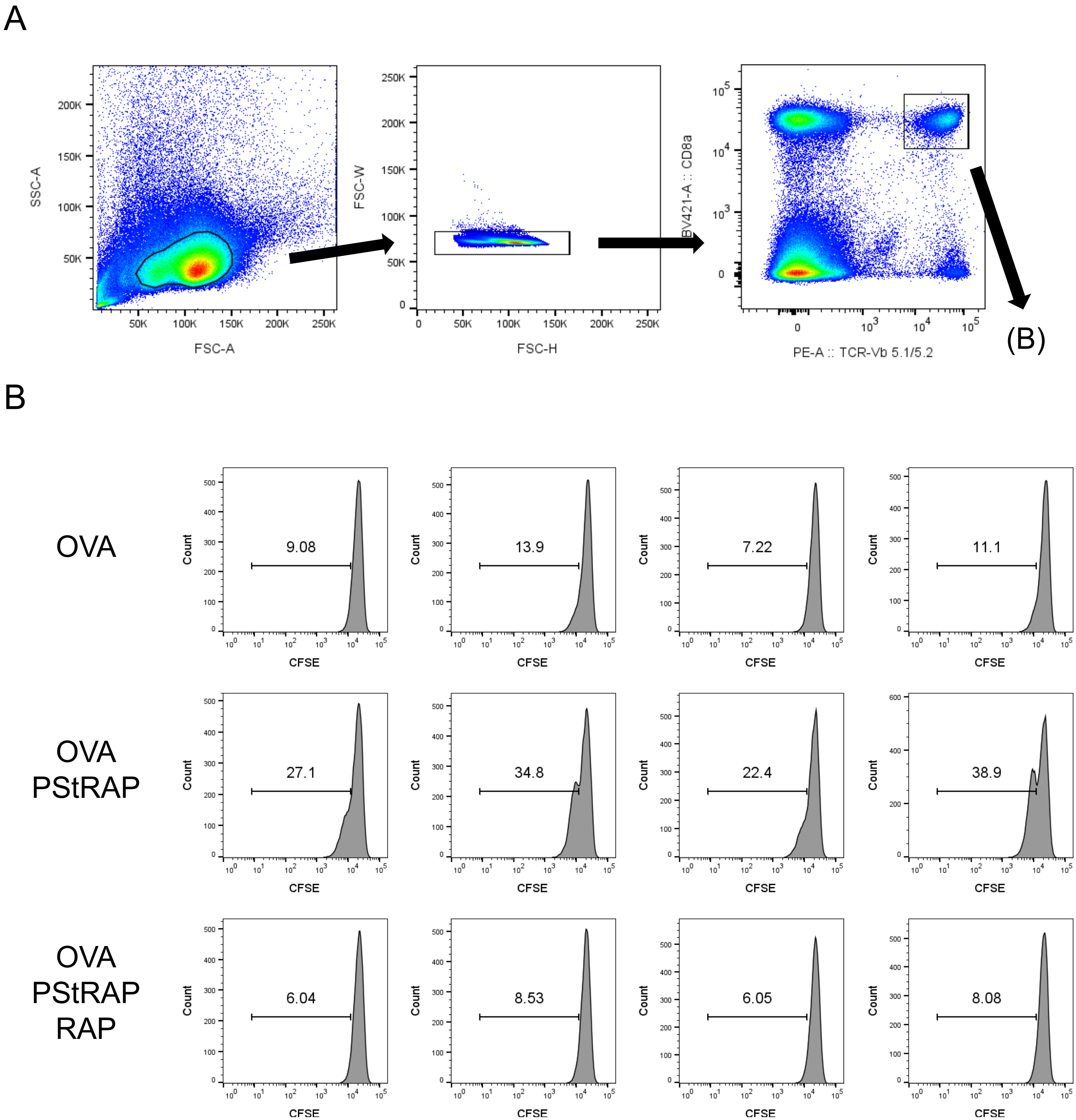

Figure S8. *In vivo* CD8⁺ T-cell proliferation assay.
(A) Gating strategy for the detection of adoptively transferred $CD8^+$ T-cells from the OT-I mouse. The both TCR-Vβ5.1/5.2- and CD8α-positive cells were defined by gating FSC/SSC and then single cell-populations, in that order. (B) Histograms from the flow cytometry analysis of gated TCR-$V\beta5^+CD8^+$ OT-I cells from representative mice of each group (n = 4). The dividing OT-I cell rate is depicted as a percentage in Figure 3C.

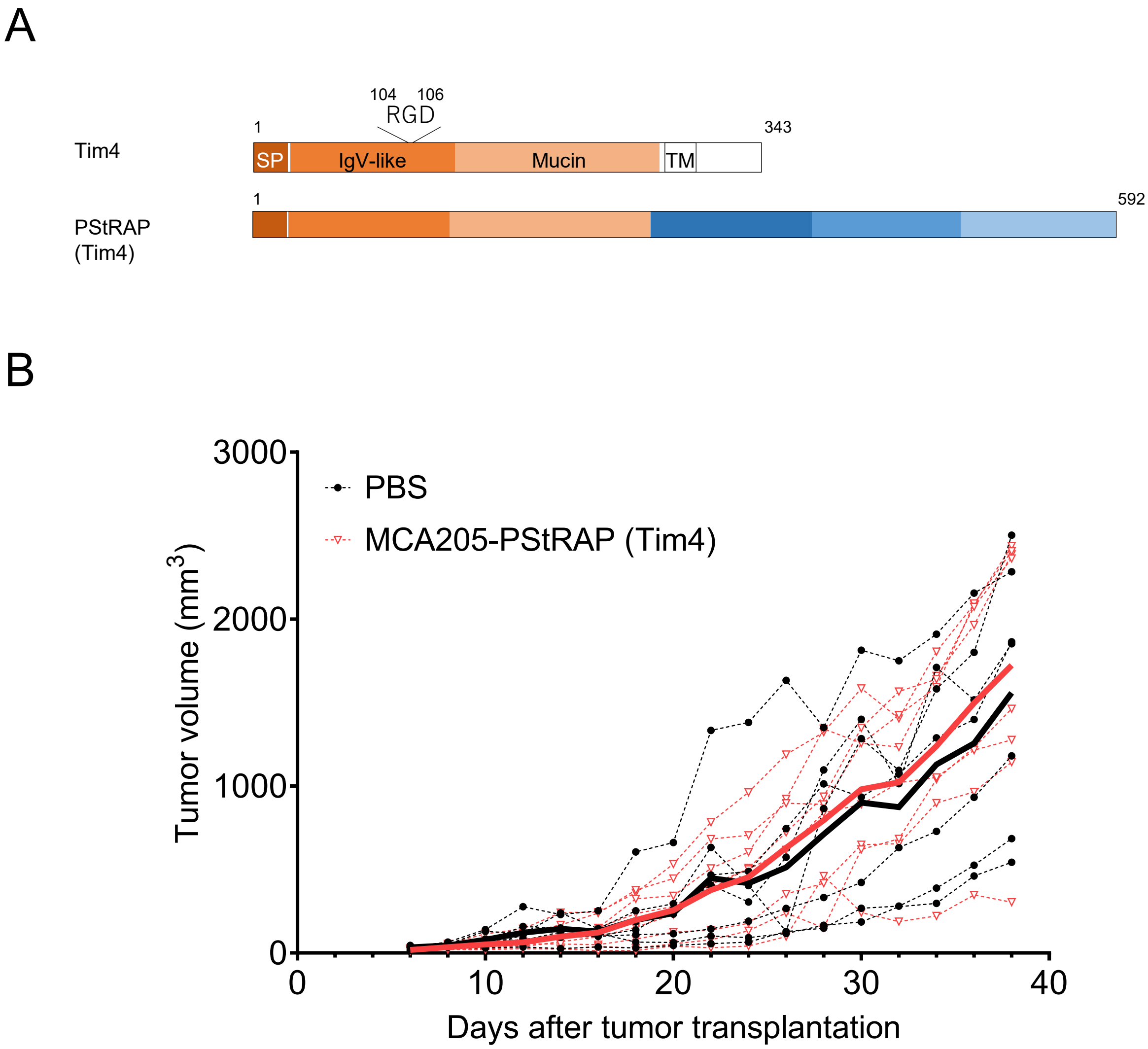

Figure S9. Structure and function of alternative PStRAP (Tim4).
(A) Structure of Tim4 and PStRAP (Tim4). SP; signal peptide, TM; transmembrane region. (B) MMC-pulsed MCA205-PStRAP (Tim4) (5×10^5 cells) cells were subcutaneously inoculated into the left frank of the mice. Viable MCA205 (5×10^5 cells) cells subcutaneously transplanted into the right frank seven days later, and then tumor volumes were measured every other day (*n* = 8/group). PBS group was same data in Figure 3E. Significance differences were determined using two-way ANOVA.

A

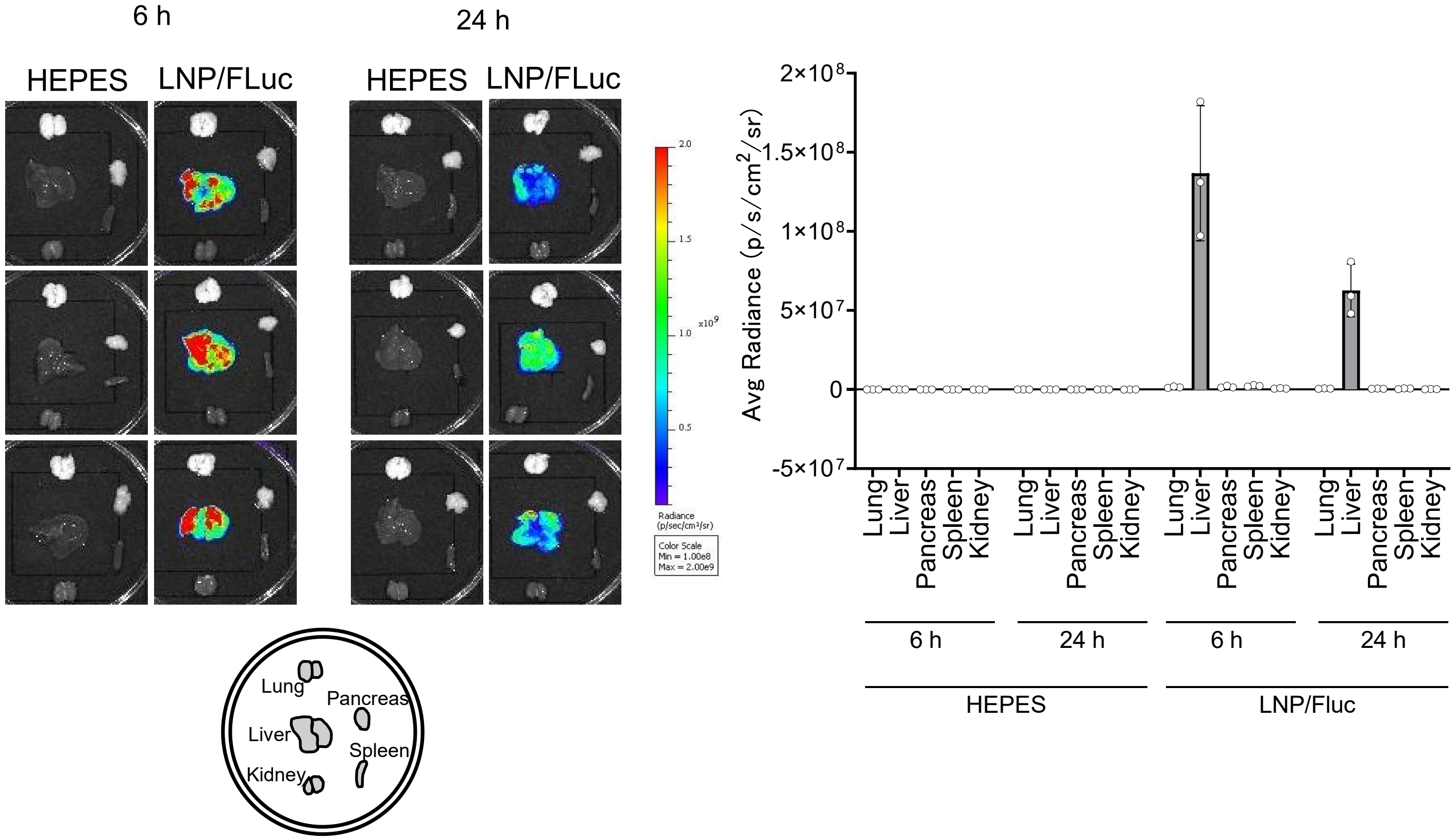
6 h
24 h
HEPES
LNP/FLuc
HEPES
LNP/FLuc
Radiance (p/sec/cm²/sr)
Color Scale Min = 1.00e8 Max = 2.00e9
Lung
Pancreas
Liver
Spleen
Kidney
Avg Radiance (p/s/cm²/sr)
2×10⁸
1.5×10⁸
1×10⁸
5×10⁷
0
-5×10⁷
Lung
Liver
Pancreas
Spleen
Kidney
6 h
24 h
HEPES
LNP/Fluc

B

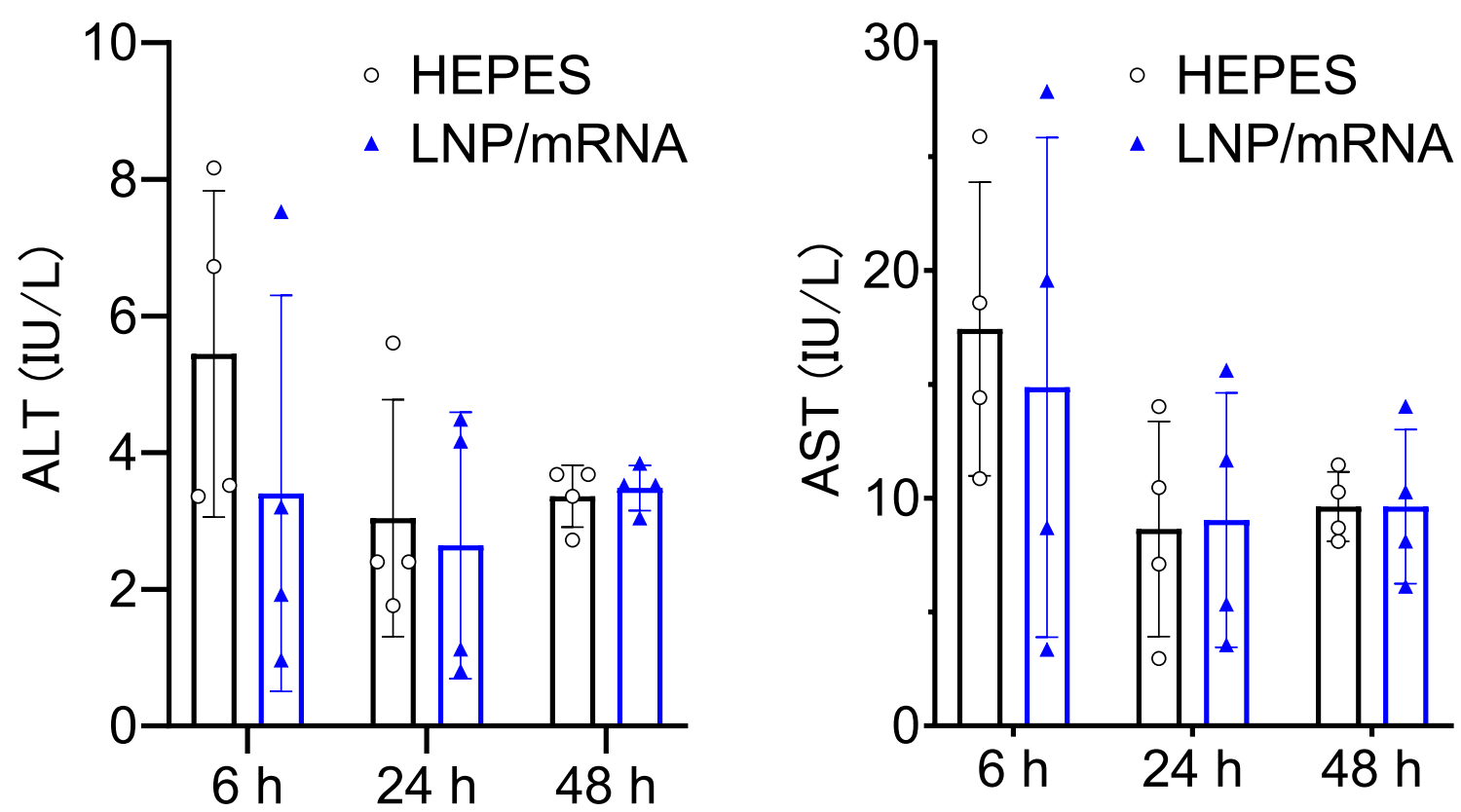
HEPES
LNP/mRNA
ALT (IU/L)
AST (IU/L)
6 h
24 h
48 h

C

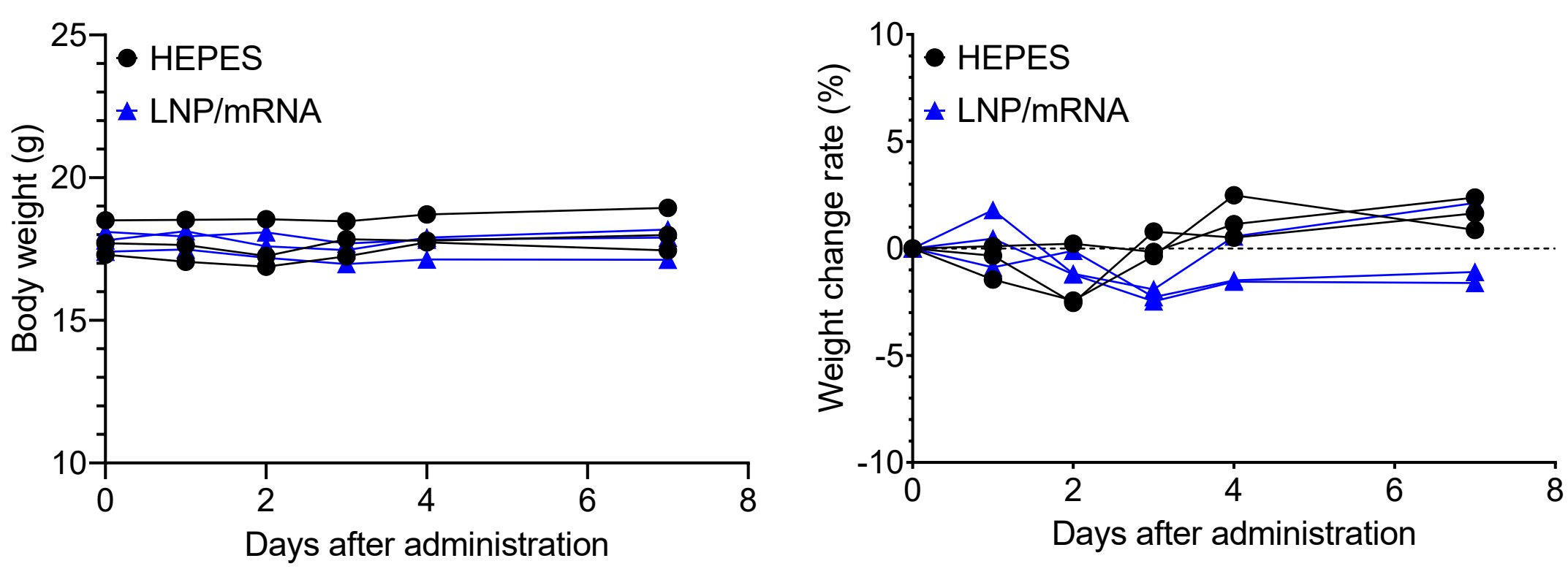
HEPES
LNP/mRNA
Body weight (g)
Weight change rate (%)
Days after administration

Figure S10. LNP/mRNA delivery and toxicity.
(A) The luminescence of the desired mouse organs (*n* = 3/group) at 6 and 24 hours (h) after intravenous LNP/Fluc or HEPES injection are shown. Error bars show mean (SD). (B) The amounts of ALT and AST in serum from mice (*n* = 4/group) were measured at the indicated time points after intravenous LNP/mRNA or HEPES injection. Error bars show mean (SD). Significance differences were determined using an unpaired Welch's *t*-test. (C) The body weights of the mice (*n* = 3/group) were measured for seven days after intravenous LNP/mRNA or HEPES injection, and fluctuations (left) and change rates (right) are indicated. Significance differences were determined using two-way ANOVA.
